# Activation of Vitamin D Receptor Pathway Enhances Differentiating Capacity in Acute Myeloid Leukemia with Isocitrate Dehydrogenase Mutations

**DOI:** 10.3390/cancers13205243

**Published:** 2021-10-19

**Authors:** Marie Sabatier, Emeline Boet, Sonia Zaghdoudi, Nathan Guiraud, Alexis Hucteau, Nathaniel Polley, Guillaume Cognet, Estelle Saland, Laura Lauture, Thomas Farge, Ambrine Sahal, Vera Pancaldi, Emeline Chu-Van, Florence Castelli, Sarah Bertoli, Pierre Bories, Christian Récher, Héléna Boutzen, Véronique Mansat-De Mas, Lucille Stuani, Jean-Emmanuel Sarry

**Affiliations:** 1 Centre de Recherches en Cancérologie de Toulouse, Université de Toulouse, Inserm, Centre National de Recherche Scientifique, CEDEX 1, 31037 Toulouse, France; marie.sabatier@inserm.fr (M.S.); emeline.boet@inserm.fr (E.B.); sonia.zaghdoudi@inserm.fr (S.Z.); nathan.guiraud@inserm.fr (N.G.); alexis.hucteau@inserm.fr (A.H.); Nathaniel.polley@inserm.fr (N.P.); guillaume.cognet@inserm.fr (G.C.); estelle.saland@inserm.fr (E.S.); laura.lauture@inserm.fr (L.L.); thomas.farge@inserm.fr (T.F.); ambrine.sahal@inserm.fr (A.S.); vera.pancaldi@inserm.fr (V.P.); bertoli.sarah@iuct-oncopole.fr (S.B.); recher.christian@iuct-oncopole.fr (C.R.); Helena.Boutzen@uhnresearch.ca (H.B.); demas.veronique@iuct-oncopole.fr (V.M.-D.M.); 2 LabEx Toucan, 31037 Toulouse, France; 3 Equipe Labellisée Ligue Nationale Contre le Cancer 2018, 31037 Toulouse, France; 4 CEA/DSV/iBiTec-S/SPI, Laboratoire d’Etude du Métabolisme des Médicaments, MetaboHUB-Paris, 91191 Gif-sur-Yvette, France; emeline.chu-van@cea.fr (E.C.-V.); Florence.castelli@cea.fr (F.C.); 5 Département d’Hématologie, University of Toulouse, CEDEX 6, 31013 Toulouse, France; 6 Service d’Hématologie, Institut Universitaire du Cancer de Toulouse-Oncopole, CHU de Toulouse, CEDEX 9, 31059 Toulouse, France; 7 Réseau Régional de Cancérologie Onco-Occitanie, CEDEX 9, 31059 Toulouse, France; bories.pierre@iuct-oncopole.fr

**Keywords:** AML, IDH, vitamin D, VDR, ATRA, CEBPα, differentiation

## Abstract

**Simple Summary:**

Around 15% of acute myeloid leukemia (AML) patients harbor mutations in isocitrate dehydrogenases (IDH), which lead to the production of the oncometabolite 2-hydroxyglutarate (2-HG). Inhibitors of mutant IDH enzymes and their 2-HG production have been approved by the FDA to be used in patients. However, 60% of IDH mutant AML patients do not respond to these inhibitors or develop mechanisms of resistance, leading to relapse. Among these mechanisms, some produce a 2-HG rebound. Alternative therapies exploiting the 2-HG-dependent molecular effects could therefore be of clinical interest. In this study, we demonstrate that 2-HG specifically activates vitamin D receptor (VDR) in IDH mutant AML cells leading to increased sensitivity to the combination of vitamin D (or VDR agonist) and all-*trans* retinoic acid and revealing a new therapeutic approach that can be readily applied to AML patients in this subgroup.

**Abstract:**

Relapses and resistance to therapeutic agents are major barriers in the treatment of acute myeloid leukemia (AML) patients. These unfavorable outcomes emphasize the need for new strategies targeting drug-resistant cells. As IDH mutations are present in the preleukemic stem cells and systematically conserved at relapse, targeting IDH mutant cells could be essential to achieve a long-term remission in the IDH mutant AML subgroup. Here, using a panel of human AML cell lines and primary AML patient specimens harboring IDH mutations, we showed that the production of an oncometabolite (R)-2-HG by IDH mutant enzymes induces vitamin D receptor-related transcriptional changes, priming these AML cells to differentiate with pharmacological doses of ATRA and/or VD. This activation occurs in a CEBPα-dependent manner. Accordingly, our findings illuminate potent and cooperative effects of IDH mutations and the vitamin D receptor pathway on differentiation in AML, revealing a novel therapeutic approach easily transferable/immediately applicable to this subgroup of AML patients.

## 1. Introduction

Recurrent mutations in isocitrate dehydrogenases IDH1 or IDH2 are detected in more than 15% of newly diagnosed AML patients and lead to the production of the oncometabolite (R)-2-hydroxyglutarate (2-HG) [1]. IDH mutation and 2-HG induce a profound remodeling of the epigenetic landscape of AML cells, disrupting hematopoietic differentiation [2,3]. In this context, we have previously shown that IDH1 mutation primes AML cells to the myeloid differentiation pathway through a 2-HG-mediated activation of the transcription factor CEBPα that sensitizes IDH1 mutant (IDH1^MUT^) AML cells to differentiation therapy using all-*trans* retinoic acid (ATRA) [4]. Consistent with this study, Mugoni et al. have further demonstrated that IDH2^MUT^ AML are sensitive to acute promyelocytic leukemia (APL)-selective combination therapy (ATRA+arsenic trioxide) [5]. Moreover, since IDH mutations are early events in leukemogenesis and are conserved at relapse [6], these enzymes represent attractive therapeutic targets. Therefore, pharmacological agents that specifically block 2-HG production have been developed and some of them recently approved by the FDA [7]. However, mechanisms of primary and secondary resistance to these agents such as FLT3/RAS mutation co-occurrence or second-site mutation/isoform switching have been already described and can lead to a rebound of 2-HG production [8,9,10]. This emphasizes the crucial role of this oncometabolite in IDH^MUT^-AML maintenance and highlights the need of alternative therapeutic solutions.

Here, we report that 2-HG and IDH mutation activated the vitamin D3 receptor (VDR) pathway in a CEBPα-dependent manner in AML. This favored IDH mutant AML cells to VD-induced myeloid differentiation and enhanced its synergistic effect with ATRA. This study provides a scientific rationale to treat IDH mutant AML subgroup, as an alternative to IDH-inhibitor treatment or for patients relapsing from it.

## 2. Materials and Methods

### 2.1. Primary AML Cells

De-identified primary AML patient specimens are from Toulouse University Hospital (TUH) (Toulouse, France). Frozen samples were obtained from patients diagnosed with AML at TUH after signed written informed consent for research use in accordance with the Declaration of Helsinki and stored at the HIMIP collection (BB-0033-00060). According to the French law, HIMIP biobank collection has been declared to the Ministry of Higher Education and Research (DC 2008-307, collection 1) and obtained a transfer agreement for research applications (AC 2008-129) after approbation by our institutional review board and ethics committee (Comité de Protection des Personnes Sud-Ouest et Outremer II). Clinical and biological annotations of the samples have been declared to the CNIL (Comité National Informatique et Libertés, i.e., Data Processing and Liberties National Committee). Patient characteristics are summarized in Figure S1A. Human primary AML cells were grown in H4230 methylcellulose medium (STEMCELL Technologies, Vancouver, BC, Canada) supplemented with 10% 5637-conditioned medium with or without ATRA (from 0.1 µM to 1 µM) and 1,25-Dihydroxyvitamin D3 (VD; from 10 nM to 100 nM) alone or in combination during 7 to 14 days at 37 °C with 5%CO_2_.

### 2.2. Mice and Patient-Derived Xenografted Model

De-identified animals were used in accordance with a protocol reviewed and approved by the Institutional Animal Care and Use Committee of Région Midi-Pyrénées (Toulouse, France). NSG mice were produced at the Génotoul-Anexplo platform at Toulouse, France, using breeders obtained from Charles River Laboratories. Mice were housed in sterile conditions using high-efficiency particulate arrestance filtered microisolators and fed with irradiated food and sterile water.

Mice (6–9 weeks old) were sub-lethally treated with busulfan 30 mg/kg 24 h before injection of leukemic cells. Leukemia sample of IDH1^R132H^ AML patient was thawed at room temperature, washed twice in PBS, and suspended in Hank’s balanced salt solution at a final concentration of 2–10 × 10^6^ cells for PDXs per 200 µL of Hank’s balanced salt solution per mouse for tail vein injection. Daily monitoring of mice for symptoms of disease (ruffled coat, hunched back, weakness, and reduced mobility) determined the time of killing for injected animals with signs of distress. No signs of distress were seen, and mice were initially analyzed for engraftment 10 weeks after injection.

### 2.3. AML Cell Lines

Human AML cell lines HL60, U937 were purchased at DSMZ (Braunschweig, Germany) and KG1a at ATCC (Manassas, VA, USA). THP1 expressing IDH1^WT^ were gifted by Prof. Steven Chan [11]. Clones from the HL60 cell line expressing either IDH1^WT^ (clone 2 and 7) or IDH1^R132H^ (clone 5 and 11) were generated by our team [4]. HL60 was engineered to express IDH2^WT^ or IDH2^R172K^ as described below. All the cells excluding THP1 were maintained in MEMα with Glutamax (Gibco, Life Technologies, Carlsbad, CA, USA) supplemented with 10% FBS (Invitrogen, Waltham, MA, USA) in the presence of 100 U/mL of penicillin and 100 µg/mL of streptomycin. THP1 were cultivated in RPMI with Glutamax (Gibco, Life Technologies, CA, USA) supplemented with 10% FBS (Invitrogen, MA, USA) in the presence of 100 U/mL of penicillin and 100 µg/mL of streptomycin. All cell lines have been routinely tested for mycoplasma contamination in the laboratory.

### 2.4. Reagents

Octyl-2HG (1-Octyl ester of (R)-2-HG) was synthesized at the Organic Synthesis Core (Memorial Sloane-Kettering Cancer Center, New York, NY, USA) as previously described [12] and dissolved in DMSO. Octyl-OH (1-Octanol, 8.20931, Sigma, St. Louis, MI, USA) solution in DMSO as the same concentration of octyl-2-HG was used as a control solution to assure that only the effect of (R)-2-HG-free acid on the cells was observed. ATRA (R2625, Sigma) was dissolved in DMSO. VD (1,25-Dihydroxyvitamin D3, D1530, Sigma) was dissolved in ethanol.

### 2.5. Lentiviral Infection

Lentiviral infection was carried out as previously described [13]. Briefly, each construct (6 µg) was co-transfected using lipofectamine 2000 with p8.1 (4 µg) and pVSV-G (2 µg) (providing packaging and envelope proteins) into 293T cells to produce lentiviral particles. At about 72 h post transfection, 293T culture supernatants containing lentiviral particles were harvested and filtered. On the same day, HL60 or clones from HL60 cell line were infected with the lentiviral particles and polybrene (8 µg/µL) by spinoculation (800 g, 60 min, 37 °C). After 4–6 h, AML cells were washed with PBS1X, then diluted in classical culture media. Three days after lentiviral infection, cells were selected with hygromycin B (300 µg/mL) for generation of HL60 IDH2^WT^ and IDH2^R172K^ or with puromycin (1 µg/mL) for CEBPα and CEBPβ-LIP knockdown. For CEBPα and CEBPβ-LIP invalidation, pLKO vectors containing the following shRNA sequences were used (5′ > 3′): shRNA control, purchased from Sigma (SHC002 MISSION pLKO.1-puro non-mammalian shRNA control; CCGGCAACAAGATGAAGAGCACCAACTC); shRNA CEBPA, purchased from Sigma (SHCLNG-NM_004364, TRCN0000007306; CCGGGCACGAGACGTCCATCGACATCTCGAGATGTCGATGGACGTCTCGTGCTTTTT); shRNA CEBPB, purchased from Sigma (SHCLNG-NM_005194, TRCN0000007440; CCCGTGGTGTTATTTAAAGAA). For the generation of IDH2^WT^ or IDH2^R72K^ HL60, the following pSLIK vectors were used: pSLIK-IDH2-FLAG (Addgene plasmid #66806); pSLIK-IDH2-R172K-FLAG (Addgene plasmid #66807).

### 2.6. RNA Extraction and RT-qPCR

One µg RNA was isolated using the TRI-reagent and then reverse transcribed with the iScript™ cDNA Synthesis kit. The resulting cDNAs were quantified by real-time qPCR using the SYBR Green Master mix on an Applied Biosystems StepOne™. The relative mRNA levels were calculated using the 2^(−ΔCT)^ method and normalized to PPIA (cyclophilin A) mRNA level as housekeeping gene *.

### 2.7. Western Blot

Proteins were analyzed by Western Blot as previously described [13]. List of antibodies used in this work: anti-CEBPα (2841, CST, 1:1000), anti-CEBPβ (sc-150, Santa Cruz, 1:1000), anti-GAPDH (5174, CST, 1:10,000), anti-HSP90 (4874, CST, 1:1000), anti-IDH2 (ab55271, Abcam, 1:1000), anti-IDH2-R172K (ab264052, Abcam, 1:1000), anti-RARα (gifted by Prof. Hugues de Thé), anti-RXRα (3085, CST), anti-Actinin (3134, CST, 1:10,000), anti-VDR (12550, CST).

### 2.8. Analysis of Myeloid Differentiation

#### 2.8.1. Flow Cytometry

We performed FACS analysis of CD11b, CD14 and CD15 expression using the following anti-human monoclonal antibodies: CD11b-PE (Beckman Coulter, IM2581U), CD14-AF700 (BD, 557923), CD15-APC (BD, 551376), CD45-BV510 (BD, 563204) or CD45-APCH7 (BD, 641417). Apoptotic cells were stained with AnnexinV-BV421 (BD, 563973) or Annexin-FITC (BD, 556420) according to the manufacturer’s protocol (BD).

#### 2.8.2. Morphological Characterization

For the morphological analysis of myeloid cell differentiation, we prepared cytospins by centrifugation of 200 µL of PBS1X containing 50,000 cells (800 rpm, 10 min for primary cells and 500 rpm, 4 min for clones from HL60 cell line) using SuperfrostPlus positively charged glass slides. Then, we stained cytospun slides at room temperature with May-Grünwald Giemsa. AxioCam camera (Carl Zeiss, Oberkochen, Germany) was used for image acquisition.

### 2.9. Data Exploration Mining

List of differentially expressed mRNA (FC > ±1.5 and *p*-value < 0.05) for each cohort (Table S1) were uploaded in the Genome Analyzer bioinformatics tool (Genomatix) for pharmacological substances gene signatures enrichment analyses based on the Genomatix literature mining (Tables S2 and S3). GSEA analyses was performed using GSEA version 3.0 (https://www.gsea-msigdb.org/gsea/index.jsp, accessed on 10 July 2021), Broad Institute, Boston, MA, USA). The “Late VD/VDR activation pathway” gene signature used in this study were obtained from Warwick et al. [14]. Transcriptome of HL60 IDH1^WT^, IDH1^WT^+2HG, IDH1^R132H^, IDH1^R132H^+ATRA were generated by our team [4]. Publicly accessible transcriptomic databases used in this study: GSE144684, TCGA.

### 2.10. Single Cell RNA Sequencing

Viable primary human AML cells from mice bone marrow of PDX were isolated using cell sorter cytometer. Viable AML cells were then diluted to 1500 cells/µL in PBS1X 0.05. To generate single-cell libraries the Chromium Single Cell 3’ Reagent Kits (v3 Chemistry, 10× Genomics, Pleasanton, CA, USA) was used. About 10,000 cells were added to the channel and sample was run according to manufacturer’s protocol with a targeted cell recovery estimate of 12,000 cells in total. After reverse transcription, single-cell droplets were broken and the single-strand cDNAs were isolated and cleaned. cDNAs were then amplified 12 thermal cycles. The sequencing-ready library was cleaned up prior to sequencing on a NextSeq550 instrument (Illumina, San Diego, CA, USA). The output bcl files were demultiplexed to FASTQ format by using bcl2fastq. FASTQ reads were aligned and converted into gene expression matrices with CellRanger-3.0.2. Expression matrices were loaded in an R session with the Seurat 4.0 package [15], which facilitated the normalization and variance stabilization of the data using the sctransform function [16]. For quality control purposes, only viable cells with a mitochondrial RNA expression ratio of 0.2 or below were maintained. Further filtering parameters include the exclusion of cells with unique molecular identifiers (UMIs) less than 500, number of genes less than 250, and log10 (genes per UMI) less than 0.80. To visualize and infer common gene expression with minimal batch effect, samples were integrated by status of treatment (diagnosis, MRD, and relapse). Cell types were categorized with the “Hay Bone Marrow” signature collection from the Human Cell Atlas. After applying the AddModuleScore function from Seurat to derive signature expression scores per each cell per signature, the expression values were normalized to z-scores. The greatest z-score pertained to the highest-likelihood cell type by which it was then categorized. Z-scores for cellular function signatures were ascertained using the same method and visualized using the FeaturePlot function. The plotting of the correlation among signature expressions was facilitated with the FeatureScatter Seurat function, and statistical analyses to validate these correlations were provided by the base-R function cor.test.

### 2.11. Metabolomic Analyses

#### 2.11.1. Extraction of Metabolites

Metabolite extraction was performed from frozen cell pellets (−80 °C). Briefly, pellets were mixed with 150 μL of water, and 5 μL of an external standard mixture containing 20 µg/mL of ^13^C_5_-2-HG, ^13^C_5_-α-KG were added. Samples were scrapped, extracted with 350 µL of ice-cold methanol and vortexed. Next, samples were centrifuged at 20,000× *g* for 15 min at 4 °C, and dried under a nitrogen stream using a Turbovap (Biotage) at 30 °C. Finally, samples were resuspended in 50 μL of mobile phase A (see below), vortexed and centrifuged at 4 °C for 5 min at 20,000× *g*. The resulting supernatants were then transferred into vials.

#### 2.11.2. Metabolite Quantification

Metabolites LC-HRMS quantification of 2-HG and α-KG was performed using an Ultimate 3000 chromatographic system coupled with an exactive mass spectrometer (Thermo Fisher Scientific, Courtaboeuf, France) fitted with an electrospray source. Chromatographic separation was performed on a Hypercarb column (3 μm, 2.1 × 100 mM; Thermo Fisher Scientific, Waltham, MA, USA) maintained at 30 °C. Mobile phase A consisted of an aqueous buffer of 5 mM of ammonium formate at pH 2.7, while mobile phase B was made of acetonitrile with 0.1% formic acid. Chromatographic elution was achieved at a flow rate of 300 μL/min. After injection of 10 μL of sample, elution started with an isocratic step of 0.5 min at 95% A, followed by a linear gradient from 95% to 5% of phase A in 4.5 min. The chromatographic system was then rinsed for 1 min at 95% B, and the run ended with an equilibration step of 2 min. The column effluent was directly introduced into the electrospray source of the mass spectrometer, and analyses were performed in the negative ion mode. Source parameters were as follows: capillary voltage, −2.5 kV; capillary temperature, 350 °C; sheath gas and auxiliary gas pressures set at 35 and 10 arbitrary units, respectively. The detection was performed from *m*/*z* 50 to 850 at a resolution of 50,000 (full width at half-maximum at *m*/*z* 200) using an AGC target of 3E6 and a maximum injection time of 500 ms. 2-HG and α-KG were detected as deprotonated [M–H]− species at *m*/*z* 147.0299 and 145.0142, respectively. Two-HG and α-KG, were quantified by isotope dilution using the corresponding ^13^C-labeled homologues as internal standards.

### 2.12. Statistical Analysis

GraphPad Prism Software version 9 (La Jolla, CA, USA) was used for the statistical analysis. The results were expressed as a mean ± SEM. Statistical analyses were performed using two-tailed (non-directional) Student’s t test with Welch’s correction The significance is represented by stars in which * is *p* < 0.05, ** is *p* < 0.01, *** is *p* < 0.005 and **** is *p* < 0.001. Combination indexes were calculated using Compusyn software (https://www.combosyn.com/, accessed on 6 October 2021).

## 3. Results

### 3.1. Vitamin D Receptor-Related Gene Signatures Are Enriched in Transcriptomes of IDH Mutant Cells

**Figure 1:**
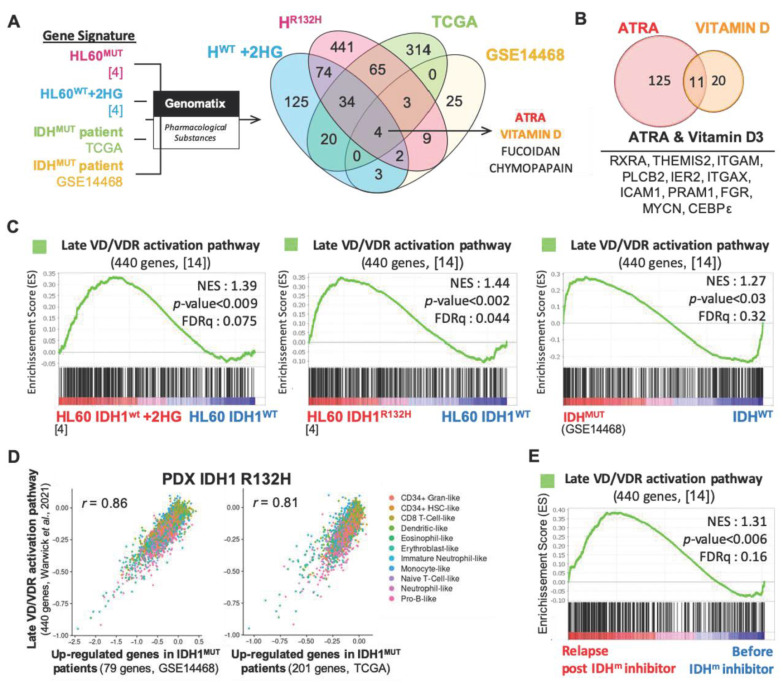
Vitamin D receptor-related gene signatures are enriched in transcriptomes of IDH mutant AML cells. (**A**) Gene signature of pharmacological substances were compared with gene signatures associated with IDH1^MUT^ HL60 cells, IDH1^WT^ HL60 cells+2HG and IDH^MUT^ patient cells from TCGA and GSE14468 transcriptomes using Genomatix software. VENN diagram represents common pharmacological substances associated with these four gene signatures. (**B**) VENN diagram of common genes from ATRA- and VD-responsive genes (Pharmacological substances, Genomatix) enriched in IDH^MUT^-AML and HL60 IDH1^WT^+2HG. (**C**) Gene set enrichment analysis (GSEA) of late VD/VDR activation pathway signature in HL60 IDH1^R132H^, HL60 IDH1^WT^ treated with 2HG and in IDH^MUT^ patients from GSE14468. (**D**) Correlation between late VD/VDR activation pathway gene signature and up-regulated genes in IDH1^MUT^ signatures (left, GSE14468; right, TCGA) in scRNA-seq of AML cells from the PDX IDH1^R132H^. (**E**) GSEA of late VD/VDR activation pathway gene signature in IDH^MUT^ patients relapsing after IDH inhibitor treatment compared to those patients before treatment (GSE153349).

To identify novel therapeutic agents for the treatment of AML harboring IDH mutation, we first performed transcriptomic analyses of two published independent cohorts of AML patients (GSE14468 [17], TCGA) and HL60 AML cell line expressing either IDH1^R132H^ or IDH1^WT^ (Table S1) [4]. We identified four pharmacological substance-associated gene signatures enriched in both IDH^MUT^ AML patients, HL60 IDH1^R132H^ and HL60 IDH1^WT^ treated with 2-HG including ATRA and VD (Figure 1A and Table S2). Both ATRA and VD are differentiating agents known to induce granulocytic and monocytic differentiation in AML, respectively [18]. Among the enriched genes in IDH^MUT^ and IDH1^WT^+2HG AML cells associated with these two small molecules, we identified common genes related to monocytic and granulocytic differentiation (RXRα, ITGAM or CD11b, ITGAX or CD11c, CEBPε) (Figure 1B and Table S3). Using gene set enrichment analysis (GSEA), we further confirmed the enhanced gene expression of VDR pathway in IDH1^R132H^ HL60 cells compared to wild-type isoforms (Figure 1C and Figure S1A). Particularly, the VDR-specific gene signature of late VR response (generated from Warwick et al. 2021 [14], Table S4) is further enriched in transcriptome of IDH1^WT^ AML cells treated with exogenous 2-HG and in patients harboring IDH mutations (Figure 1C and Figure S1B). Using scRNA-seq of human IDH1^R312H^ AML blasts from a patient-derived xenografted (PDX) model in NSG mice, we confirmed this phenotype in vivo. Of note, the Late VD/VDR activation pathway gene signature was strongly correlated to gene signatures of IDH1^MUT^ patients generated from two independent public cohorts in the PDX IDH1 R132H (Figure 1D). This correlation is independent from the cell types (Figure S1C). Finally, transcriptomes of relapsing patients post-IDH^MUT^ inhibitors (NCT01915498, NCT02074839; GSE153349 [19]) are also enriched in VDR gene signatures compared to those from patients before receiving IDH inhibitors therapies (Figure 1E).

Taken together, transcriptomic analysis of cell lines, primary patient cells and PDX with IDH mutations showed that VD/VDR pathway is activated in this subgroup of AML patients. Furthermore, this VD/VDR gene signature is enhanced in patients who are relapsing from IDH inhibitor treatments, suggesting that Vitamin D-based therapies could represent a promising alternative for these patients.

### 3.2. IDH Mutations Activate CEBPa-VDR-RXR Axis through 2HG Production

**Figure 2:**
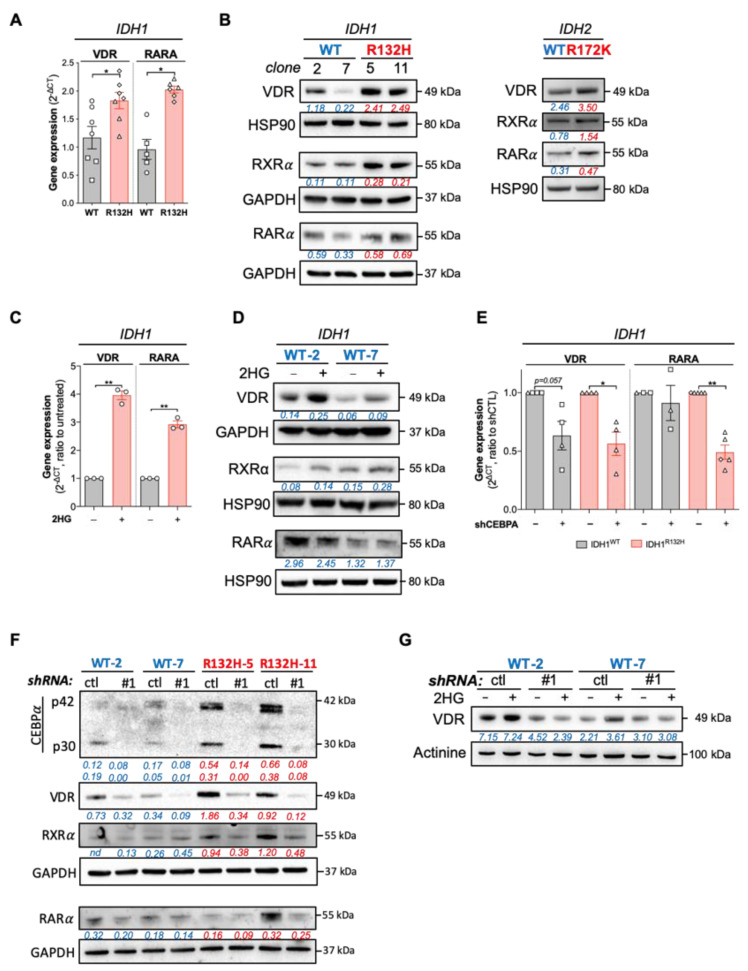
IDH mutations activate CEBPα-VDR-RXRα axis through 2HG production. (**A**) RT-qPCR showing gene expressions of VDR and RARA in HL60 IDH1^WT^ (clone 2: ○; clone 7: ☐) versus HL60 IDH1^R132H^ (clone 5: ◇; clone 11: △). (**B**) Western blot showing protein levels of VDR, RXRα and RARα in HL60 IDH1^WT^ (clones 2 and 7) versus HL60 IDH1^R132H^ (clones 5, 11 and 3) (left) and in HL60 IDH2^WT^ versus HL60 IDH2^R172K^ (right). (**C**) RT-qPCR showing gene expressions of VDR and RARA in 2HG-treated HL60 (200 µM, 1 week). (**D**) Western blot showing protein levels of VDR RXRα and RARα in 2HG-treated HL60 IDH1^WT^ (clones 2 and 7) (200 µM, 1 week). (**E**) RT-qPCR showing gene expression of and VDR and RARA in HL60 IDH1^WT^ (clone 2: ○, clone 7: ☐) and IDH1^R132H^ (clone 5: ◇, clone 11: △) after CEBPA-KD by shRNA (**F**) Western blot showing protein levels of CEBPα, VDR, RARα and VDR in HL60 IDH1^WT^ (clones 2 and 7) and IDH1^R132H^ (clone 5 and 11) after CEBPA-KD by shRNA. (**G**) Western blot showing protein levels of VDR in 2HG-treated HL60 IDH1^WT^ (clone 2 and 7) with CEBPA-KD by shRNA (200 µM, 1 week). The uncropped western blot figures are presented in Figure S6. * *p* < 0.05; ** *p* < 0.01.

Next, we analyzed the expression of VDR in HL60 expressing IDH1^WT^ or IDH1^R132H^. Consistently, we observed an increased expression in VDR, RARα (ATRA receptor/transcription factor) and RXRα (VDR and RARα co-receptor/transcription factor) in IDH1^R132H^ (Figure 2A,B, Table 1). Then, we asked if this phenotype was the same upon IDH2 mutation. To assess this question, we generated HL60 cell line expressing IDH2^WT^ or IDH2^R172K^ (Figure S2A,B). As for IDH1 mutation [4], IDH2 mutation leads to an increase in CEBPα protein expression (Figure S2A). Importantly, both mutations enhanced VDR, RXRα and RARα protein expressions compared to the WT enzymes (Figure 2B). This increase in VDR, RARα and RXRα was also induced when IDH1^WT^ HL60 cells were treated with 2-HG (Figure 2C,D).

We have previously shown that 2-HG production in IDH^MUT^ AML cells leads to CEBPα activation through a histone methylation-dependent mechanism [4]. Moreover, CEBPα modulates vitamin D signaling in hematopoietic, AML and breast cancer cells [14,20,21]. Furthermore, recent studies have demonstrated that demethylating agents induce VDR expression in AML and in high CEBPα breast cancer cells [14,22,23,24]. Thus, we determined whether VDR and RXRα might be downstream of CEBPα. Knockdown of CEBPα led to a decrease in VDR and RARα expression in both IDH^WT^ and IDH1^R132H^ cells, although the effect on VDR protein expression was markedly enhanced in IDH1^MUT^ cells and RXRα decrease following CEBPα silencing was only observed in IDH1^MUT^ cells compared to IDH^WT^ cells (Figure 2E,F and Figure S3A). Notably, 2-HG failed to increase VDR expression in IDH1^WT^ cells invalidated for CEBPα, confirming the crucial role of the 2-HG/CEBPα/VDR axis in IDH^MUT^ AML (Figure 2G). Next, we investigated if other transcription factors known to regulate myeloid/monocytic maturation such as CEBPβ might be enhanced by IDH mutation. Importantly, CEBPα and CEBPβ are also direct targets of VDR-dependent transcriptional regulation [21]. However, CEBPβ expression was not modified either by IDH1 mutation or 2-HG-treatment (Supplementary Figure S3A–C), suggesting that VDR pathway enhancement is regulated through CEBPα rather than CEBPβ in IDH^MUT^ AML. Accordingly, CEBPβ invalidation by shRNA failed to decrease VDR and RXR protein expression (Figure S3A). Overall, these results showed that 2-HG production and IDH mutations activate CEBPα, leading to an increase in VDR/RXRα pathway.

### 3.3. Combine Treatment with VD and ATRA Produces a Synergistic Induction of Differentiation in a CEBPα-Dependent Manner

**Figure 3:**
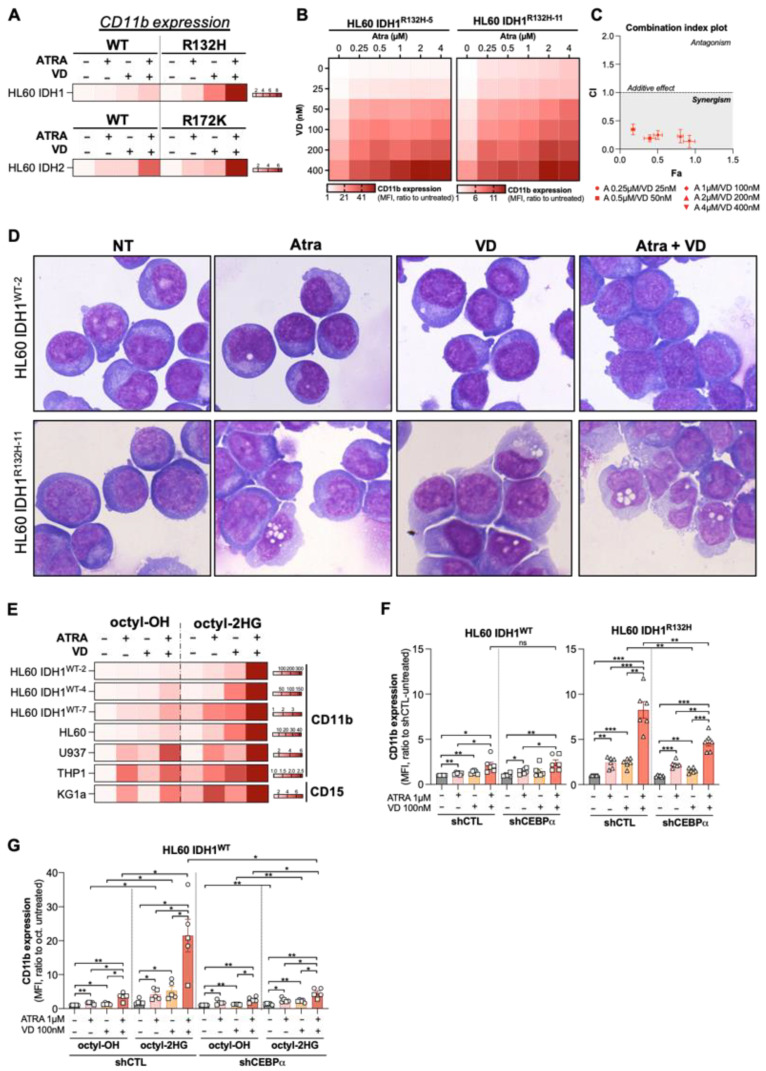
Combine treatment with VD and ATRA produces a synergistic induction of differentiation in a CEBPα-dependent manner. (**A**) HeatMap of CD11b expression (MFI, ratio to untreated) measured by flow cytometry in HL60 IDH1^WT^ versus HL60 IDH1^R132H^ and HL60 IDH2^WT^ versus HL60 IDH2^R172K^ treated for 3 days with ATRA (1 µM) and VD (100 nM) alone or in combination. (**B**) Synergy Map of CD11b expression (MFI, ratio to untreated) measured by flow cytometry in HL60 IDH1^R132H-5^ (left) and in HL60 IDH1^R132H-11^ (right) treated for 3 days with 0.25–4 µM of ATRA and 25–400 nM of VD alone or in combination. (**C**) Combination index of differentiation (CD11b expression induction) in HL60 IDH1^R132H-5^ and in HL60 IDH1^R132H-11^ treated for 3 days with ATRA and VD in combination with constant ratio of each drug. (**D**) Morphological characterization by MGG staining of HL60 IDH1^WT-2^ versus HL60 IDH1^R132H-11^ treated for 5 days with ATRA 1 µM and VD 100 nM alone or in combination. (**E**) HeatMap of CD11b expression and CD15 expression (MFI, ratio to untreated) measured by flow cytometry in 2HG-treated (100(U937)-200 µM for 1wk) HL60 IDH1^WT-2^, HL60 IDH1^WT-4^, HL60 IDH1^WT-7^, HL60, U937, THP1 and KG1a treated for 3 days with ATRA (0.1 µM for U937, 1 µM for others) or VD (100 nM) alone or in combination. (**F**) CD11b expression (MFI) measured by flow cytometry in HL60 IDH1^WT^ (clone 2: ○, clone 7: ☐) shCTL vs. shCEBPα (left panel) and in HL60 IDH1^R132H^ (clone 5: ◇, clone 11: △) shCTL vs. shCEBPα (right panel) treated for 3 days with ATRA (1 µM) and VD (100 nM) alone or in combination. (**G**) CD11b expression (MFI) measured by flow cytometry in 2HG-treated HL60 IDH1^WT^ (clone 2: ○, clone 7: ☐) shCTL vs. shCEBPα (2HG 200 µM for 3 days) treated for 3 days with ATRA (1 µM) and VD (100 nM) alone or in combination. * *p* < 0.05; ** *p* < 0.01, *** *p* < 0.005.

Lastly, transcriptomes of IDH1^R132H^ HL60 treated with ATRA were also enriched in genes related to VD (Supplementary Figure S4A and Table S5), suggesting a putative synergistic effect of these two differentiating agents in this cellular context. To further explore the therapeutic benefit of these agents for AML patients with IDH mutation, we treated primary IDH^MUT^ AML cells with ATRA or VD alone or in combination. We analyzed mono-granulocytic marker expression by flow cytometry in AML cell lines (CD11b) and primary patient cells (CD11b, CD15, CD14) with IDH^WT^ or IDH^MUT^. We first observed that the combination therapy induced a higher differentiation potentiation as shown with an increased CD11b expression in IDH1^R132H^ and IDH2^R172K^ HL60 cells compared to IDH^WT^ HL60 cells (Figure 3A and Figure S4B). Combination index analysis showed that VD and ATRA acted synergistically to induce CD11b differentiation markers expression in IDH1^R132H^ HL60 cells (Figure 3B,C). Then, morphological analysis confirmed the enhancement of myeloid differentiation characterized by an increase in number of monocyte-like cells in IDH1^R132H^ AML cells upon doublet therapy (Figure 3D).

Consistent with the enhanced activation of the VDR and ATRA pathways in HL60 IDH1^WT^+2HG (Figure 2C,D), 2-HG pre-treatment of IDH1^WT^ and four parental AML cell lines elicited a higher sensitivity to differentiation induction by ATRA/VD combination (Figure 3E and Figure S4C,D). Furthermore, CEBPα knockdown reduced significantly the response to this combination ATRA/VD in IDH1^R132H^ cells while no difference was observed in IDH1^WT^ cells (Figure 3F). Interestingly, CEBPβ knockdown also reduced the efficiency of the combination on the differentiation of IDH1^R132H^ AML cells but at a significant lower extent (Figure S4E). Finally, we showed that, in the absence of CEBPα, 2-HG no longer enhanced sensitivity to ATRA/VD combination-induced differentiation of IDH1^WT^ cells (Figure 3G).

**Figure 4:**
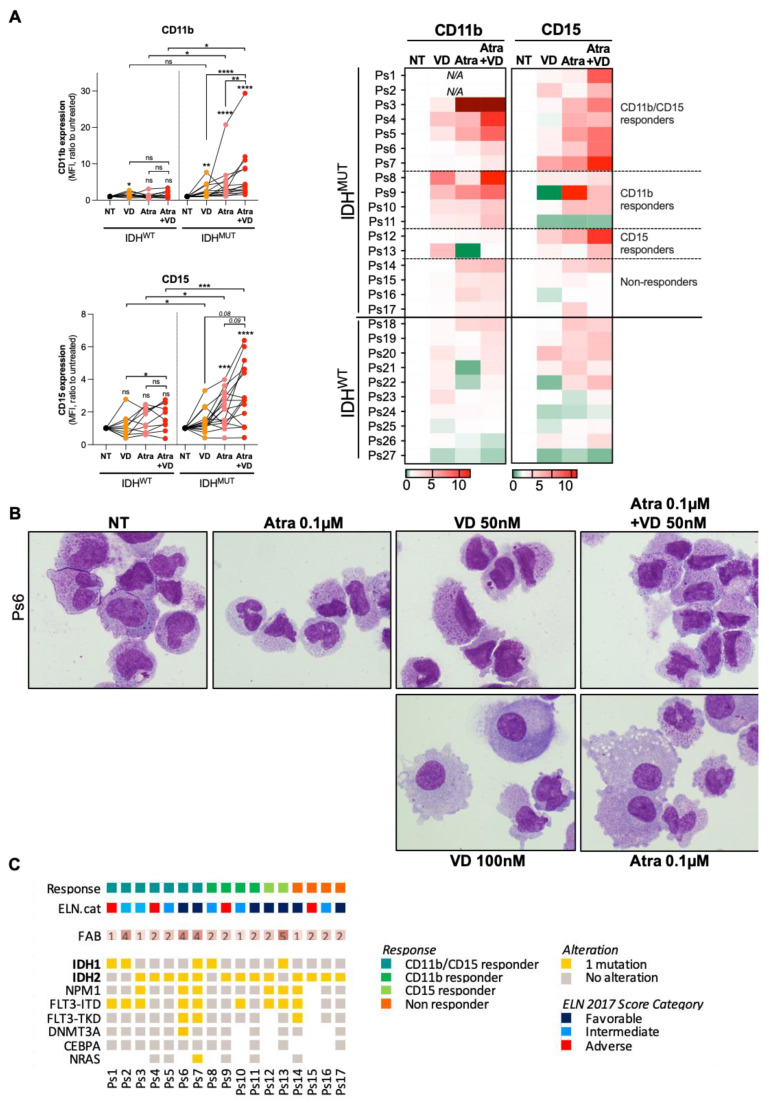
Targeting vitamin D receptor pathway enhances anti-AML effect with ATRA in IDH^MUT^ AML patients. (**A**) CD11b and CD15 expressions (mean fluorescence intensity, MFI) measured by flow cytometry in IDH^MUT^ versus IDH^WT^ patients treated with ATRA and VD alone or in combination (left panel). Results for each patient are represented individually (right panel). (**B**) Morphological characterization by MGG staining of Ps6 treated for 14 days with ATRA 0.1 µM and VD 50–100 nM alone or in combination. (**C**) Clinical characteristics of IDH^MUT^ patients in function of their response to ATRA/VD-duplet therapy. * *p* < 0.05; ** *p* < 0.01, *** *p* < 0.005, **** *p* < 0.001.

Despite phenotypical heterogeneity between patients, we also observed a marked enhancement of differentiation induction when ATRA was used in combination with VD in 13 out of 17 primary IDH^MUT^ AML cells compared to 10 primary IDH^WT^ AML cells (Figure 4A and Figure S5A,B; patient characteristics are summarized in Table S5). Morphological characterization of AML blasts from Ps6 using MGG staining confirmed the analysis of myeloid differentiation markers with an increased number of granulocytic/monocytic-like cells upon ATRA/VD (Figure 4B). Then, we classified IDH^MUT^ patients according to the efficiency of ATRA and VD co-treatments to enhance differentiation when they were associated. We clustered the patients in four following groups: CD11b/CD15 responders (*n* = 7), CD11b responders (*n* = 4), CD15 responders (*n* = 2) and the non-responders (*n* = 4) (Figure 4A). Next, we compared these groups with the clinical characteristics of these IDH^MUT^ patients. We did not observe any correlation between the differentiation response and other mutations or FAB status. However, we noted that, among responders to ATRA/VD, we had a large proportion of patients with an adverse or intermediate risks according to ELN2017 stratification [25] (Figure 4C). This result suggests that ATRA/VD combination therapy represents a promising therapeutic approach for IDH^MUT^ AML patients with a poor prognosis.

## 4. Discussion

The past few years have witnessed major advances in the therapeutic landscape of AML treatment [7]. Translational research has led to a better understanding of the impact of multiple recurrent mutations, leading to the successful development and approval of targeted therapies in several subgroups of patients, including the ones harboring IDH mutations [26]. While small-molecule IDH^MUT^ inhibitors allow promising partial or complete responses rates in AML patients, about 60% of them do not respond to the therapy or relapse [27,28,29]. Very importantly, 2-HG level is reduced in nearly all patients treated with IDH^MUT^ inhibitors, including non-responders, revealing that 2-HG reduction is not sufficient to predict clinical response to these inhibitors [8,30]. This observation may also indicate that the ability of primary patient cells to elicit alternative resistance mechanisms will dictate the clinical benefit of the IDH^MUT^ inhibitors. Some of these mechanisms notably induce a 2-HG rebound through second-site mutation or isoform-switching [8,9]. Exploiting the 2-HG specific effects in IDH^MUT^ AML cells could therefore be an alternative therapeutic approach for patients who are non-responding or relapsing from IDH^MUT^ inhibitors treatments. In this study, we demonstrated that 2-HG mediates the activation of VDR pathway through CEBPα in IDH^MUT^ AML, sensitizing IDH^MUT^ AML cells to the differentiation-based ATRA/VD doublet therapy. In this context, we proposed the association of VD with ATRA—both already used in clinic—as a promising approach to induce terminal differentiation of leukemic cells in IDH^MUT^ AML patients.

Numerous studies have investigated the potential benefit of VD-induced differentiation through interaction with VDR in different cancers including AML, without encouraging results [31]. Indeed, regulation of VDR is complex and highly dependent on the cellular context. AML cell line KG1 has been shown to be resistant to VD-induced monocytic differentiation but sensitive to ATRA-induced granulocytic differentiation, while HL60 displayed inverse responses to these two agents [32]. ATRA treatment enhances VDR expression in KG1 cells, resulting in increased differentiation using the ATRA+VD combination. As opposed, ATRA treatment reduces VDR gene expression in HL60 cells, leading to no better response to the ATRA+VD combination [32]. In our study, we showed that IDH^MUT^ through production of 2-HG increases VDR expression, which could therefore explain why VD addition to ATRA induced even more differentiation in all the different cell lines tested, including notably HL60. Interestingly, VD induced CEBPα in breast cancer MCF-7 cells and CEBPα knockdown suppressed VD anti-proliferative effects. As opposed, in MDA-MB-231 breast cancer cells non-responsive to VD treatment, CEBPα was not detected and CEBPα over-expression restored VD sensitivity [21]. Additional studies demonstrated that VD rather induced CEBPβ in kidney and osteoblasts cells [33] and in HL60 AML cell line [34], for which CEBPβ silencing inhibited VD-induced differentiation [35]. Of note, exogenous 2-HG can inhibit proliferation and viability in some leukemia cell lines [36]. In these sensitive IDH^WT^ AML cells, 2-HG targets the fat mass and obesity-associated protein (FTO), that leads to MYC/CEBPα inhibition. However, most IDH^MUT^ AML cells upregulate MYC which counteracts the effect of 2-HG on proliferation, CEBPα expression and metabolic consequences [36,37]. Indeed, for AML cells harboring IDH mutation, myeloid lineage-TF CEBPα is one of the key downstream pathways that are critical for IDH^MUT^ AML biology [4,13]. In these cells, the activation of CEBPα induces the expression of both RXRα and VDR, that primes to VD+ATRA combination therapy. In particular, 2-HG-induced epigenetic changes (e.g., histone methylation) might affect the chromatin accessibility status and structure, favoring VDR DNA binding with pioneer factors such as PU.1 or/and CEBPα [20,38]. Noteworthy, Warwick et al. (2021) showed that the regulatory network of VD response is associated with 47 transcription factors and that its transcriptional hierarchy is specifically and epigenetically dependent on VDR and CEBPα in monocytic AML THP1 cells [14]. CEBPα silencing downregulates and inactivates more than half of VD target genes [14]. Therefore, further similar molecular studies should be performed to identify new transcriptional partners and VD targets specific to IDH^MUT^ context. Markedly, Glass et al. showed a differential CpG methylation in RXR promoter specifically in IDH2^MUT^ AML cells [39]. Overall, IDH mutations seems to profoundly disrupt the epigenetic landscape of AML cells leading to the acquisition of a specific transcription factor regulation network responsible of their sensitivity to differentiating agents. Here, we demonstrated that CEBPα is crucial to ATRA and VD-induced differentiation response as knockdown of CEBPα, and CEBβ to significant lower extent, decreased IDH^MUT^ differentiation and almost completely abrogated differentiation in IDH^WT^ treated with exogenous 2-HG. Of note, high serum 2-HG and low serum VD levels are associated with shorter overall survival [40,41,42], they therefore might represent potential biomarkers to predict response to ATRA/VD doublet therapy for IDH^MUT^ AML patients. Although randomized clinical trials are still lacking, epidemiological and preclinical data suggest that VD-based combinations could be a promising therapeutic strategy by activating VD signaling pathway. In this context, better designs of combinations would circumvent potential limitations of VD alone such as high risk of hypercalcemia.

Finally, hypomethylating agents such as 5-azacytidine (AZA) or decitabine induce an increase in expression of VDR and its target genes in breast cancer cells (Marik et al. [22]) and AML cells [23]. In vivo treatment of leukemic mice with VDR agonist INEC, AZA alone, and in combination led to no difference in mice survival for AZA alone and to a significant but similar increase in mice survival for INEC alone and INEC+AZA [23]. As 2-HG increases DNA methylation in IDH^MUT^ cells, the use of a hypomethylating agent would be relevant in this subgroup of patients. Surprisingly, as opposed to first studies (DiNardo et al., Leuk Lymphoma, 2014; [43]), AML and myelodysplastic patients with IDH mutations did not display a better response to AZA monotherapy in more recent reports [44,45,46]. Moreover, AZA reduced serum and urine 2-HG levels of 2 responding patients in a first study [47]. In a second cohort of 26 patients including 7 responders to AZA, AZA tends to decrease serum 2-HG level in the 7 responders but not in the 19 non-responders [46]. We showed that VD efficiency is increased by 2-HG and, while still needing confirmation on larger cohorts, these clinical results would argue that AZA+VD could be a good combination for AML patients with IDH^MUT^ non-responding to AZA. A current recruiting phase 2 clinical trial combining INEC and decitabine in AML patients unfit for standard chemotherapy (NCT02802267) could help determine if patients with IDH mutations are more likely to respond. If so, further studies measuring the expression of VDR and VDR target genes, as well as 2-HG level, differentiation and impact on the stem cell compartment specifically in IDH^MUT^ AML cells following these combinations would be of particular interest and help determine potential biomarkers of response.

There are several limitations to our study. Before clinical application of ATRA/VD combination therapy, these results first need to be confirmed in preclinical models. In this context, the efficacy of this doublet therapy to enhance differentiation and anti-leukemic activity should be evaluated in vivo using mice xenografted with IDH^MUT^ primary cells. This experiment could also enable to consider the potential toxicity of ATRA/VD association on normal hematopoietic stem cells. Indeed, both ATRA and VD are known to induce side effect including differentiation syndrome and hypercalcemia, respectively [48,49]. Today, differentiation syndrome is well managed in APL, as well as in patient receiving IDH^MUT^ inhibitors and treatment interruption is rarely considered [49,50]. Moreover, there is a large number of VD analogs already used in clinic which prevent VD-related hypercalcemia [48,51]. However, combination of treatments could still increase undesirable effects and toxicity. Consequently, ATRA and VD dosing needs to be precisely defined. Second, correlation analyses between 2-HG serum level in IDH^MUT^ patients and their response to ATRA/VD combination therapy are needed to consider 2-HG as a biomarker of response to this treatment. Response to IDH^MUT^ inhibitors is not associated with 2-HG levels since non-responding patients display a reduction in 2-HG amount after treatment [28,30]. In this case, 2-HG could not serve as biomarker for the use of ATRA/VD combination therapy. However, 2-HG rebound is frequently observed in relapsing patients developing resistance mechanisms to IDH^MUT^ inhibitors [8,9,10]. Therefore, measuring 2-HG represents a promising approach to stratify relapsing patients according to their potential sensitivity to ATRA/VD combination therapy if these parameters are correlated.

## 5. Conclusions

In conclusion, these data demonstrate that the VDR pathway is increased in IDH^MUT^ subgroup, especially at relapse post-IDHm inhibitor. Moreover, VD could enhance ATRA effect in AML patients with IDH mutation through a 2-HG/CEBPα/VDR axis. Thus, our study describes an alternative approach with approved differentiation drugs ATRA and VD with a highly efficient and safety profile, already in the clinics. This strategy could be specifically used for non-eligible AML patients to IDH mutant inhibitor therapies or AML patients developing resistance to newly FDA-approved IDH mutant-specific inhibitors. This is timely and clinically relevant and forms the basis of novel doublet and triplet therapies in IDH-mutated AML patients.

## Figures and Tables

**Table 1:** Sequences of primers used in this study.

Genes	Forward	Reverse
*PPIA*	5′ CTCGAATAAGTTTGACTTGTGTTT 3′	5′ CTAGGCATGGGAGGGAACA 3′
*RARA*	5′ GTCTTTGCCTTCGCCAACCA 3′	5′ GCCCTCTGAGTTCTCCAACA 3′
*VDR*	5′ CCTTCACCATGGACGACATG 3′	5′ CTTTGGTCACGTCACT 3′

## Data Availability

GEO (Gene expression Omnibus, https://www.ncbi.nlm.nih.gov/geo/, accessed on 21 November 2020).

## Supplementary Materials

The following are available online at https://www.mdpi.com/article/10.3390/cancers13205243/s1, Figure S1: Vitamin D receptor-related gene signatures are enriched in transcriptomes of IDH mutant cells, Figure S2: Generation of HL60 expressing IDH2^WT^ or IDH2^R172K^, Figure S3: IDH mutations activate CEBPα-VDR-RXRα axis through 2HG production, Figure S4: Targeting vitamin D receptor pathway enhances anti-AML effect with ATRA in a CEBPα-dependent manner, Figure S5: Targeting vitamin D receptor pathway enhances anti-AML effect with ATRA in IDH^MUT^ AML patients, Figure S6: The uncropped western blot images for Figure 2, Figures S2 and S3. Table S1: Gene signatures related to Figure 1A, Table S2: List of pharmacological substances from VENN diagram related to Figure 1A, Table S3: ATRA- and VD-responsive genes enriched in IDH^MUT^-AML and HL60^WT^+2HG, Table S4: Gene signatures used for GSEA analysis, Table S5: Clinical characteristics of AML patients used in this study and related to Figure 4.
