# Supplementary material for: Activation of Vitamin D Receptor Pathway Enhances Differentiating Capacity in Acute Myeloid Leukemia with Isocitrate Dehydrogenase Mutations
Source: Cancers (Basel). 2021 Oct 19;13(20):5243. doi: 10.3390/cancers13205243 (PMC8533831; doi:10.3390/cancers13205243)
Supplement: Supplementary file 1 [file cancers-13-05243-s001.zip › cancers-1374382-supplementary figures.pdf]

Supplementary Materials: Activation of Vitamin D Receptor Pathway Enhances Differentiating Capacity in Acute Myeloid Leukemia with Isocitrate Dehydrogenase Mutations

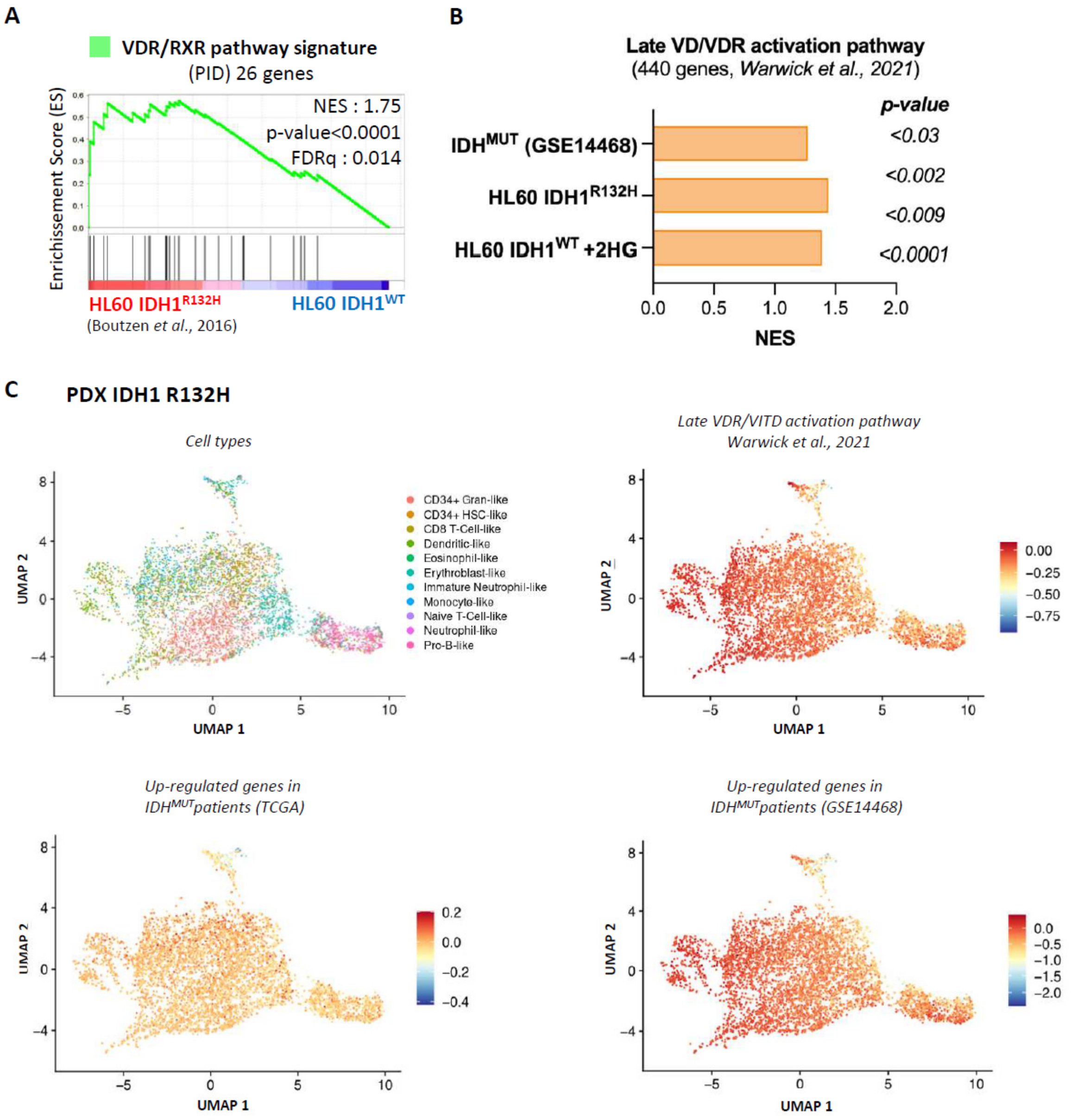

Figure S1. Vitamin D receptor-related gene signatures are enriched in transcriptomes of IDH mutant cells. (**A**) GSEA of VDR/RXR pathway signature in HL60 IDH1^{R132H} *versus* HL60 IDH1^{WT}. (**B**) Gene Set Enrichment Analysis (GSEA) of Late VD/VDR activation pathway signature in HL60 IDH1^{R132H}, HL60 IDH1^{WT} treated with 2HG and in IDH^{MUT} patients from GSE14468 related to Figure 1C (**C**) UMAP of scRNA-seq analysis of PDX IDH1^{R132H} according to cell types, Late VD/VDR activation pathway signature and IDH^{MUT} signatures generated from two independent public cohorts (TCGA and GSE14468).

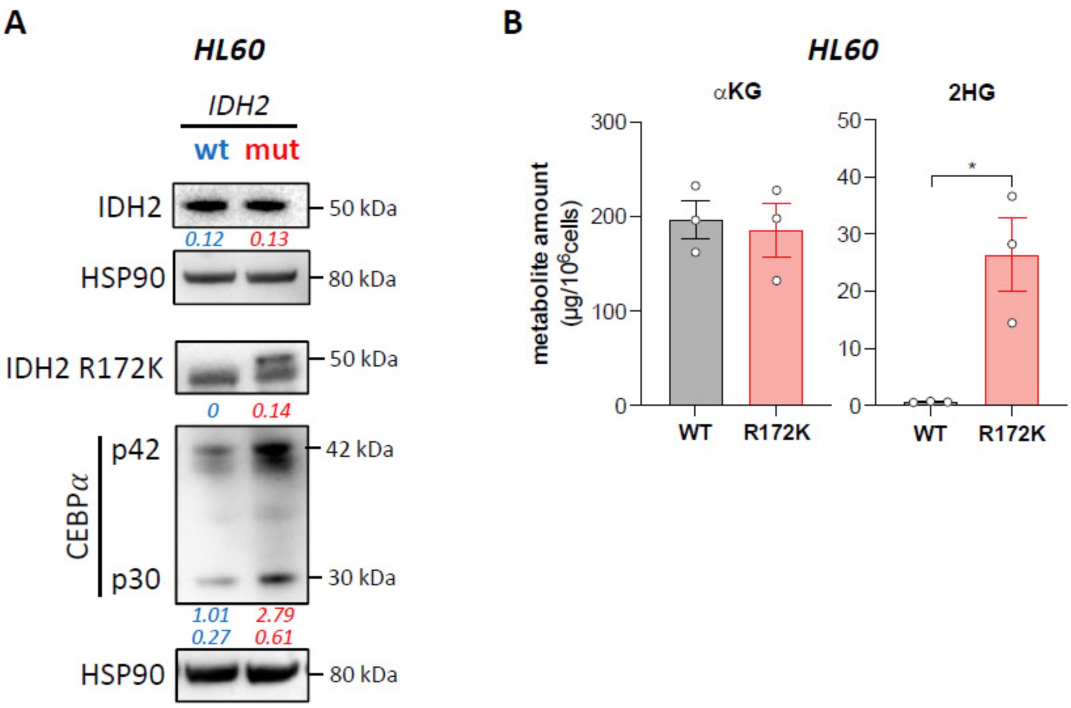

Figure S2. Generation of HL60 expressing IDH2^{WT} or IDH2^{R172K}. (**A**) Western blot showing protein levels of IDH2, IDH2 R172K and CEBPα in HL60 IDH2^{WT} versus HL60 IDH2^{R172K}. (**B**) Quantification of αKG and 2HG in HL60 IDH2^{WT} versus IDH2^{R172K}. The uncropped western blot figures were presented in Figure S6.

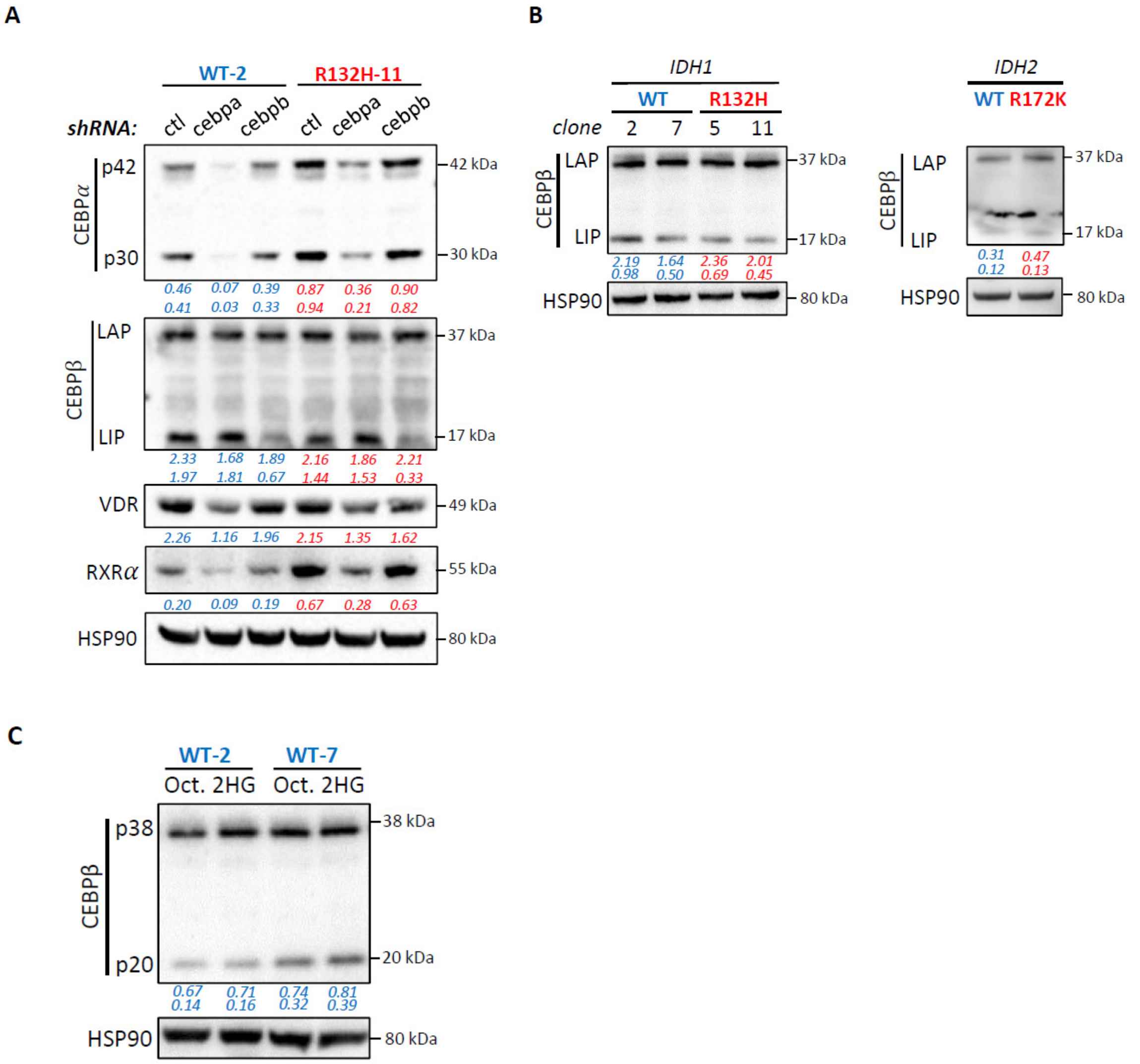

Figure S3. IDH mutations activate CEBP*α*-VDR-RXR*α* axis through 2HG production. (**A**) Western blot showing levels of CEBP*α*, CEBPβ,VDR and RXR *α* in HL60 IDH1WT-2 versus HL60 IDH1R132H-11 after CEBPA-KD and CEBPB-KD. (**B**) Western blot showing levels of CEBPβ in HL60 IDH1^{WT} versus HL60 IDH1^{R132H} and in HL60 IDH2^{WT} versus HL60 IDH2^{R172K}. (**C**) Western blot showing levels of CEBPβ in HL60 IDH1^{WT} treated for 1 week with 2HG (200 μM). The uncropped western blot figures were presented in Figure S6.

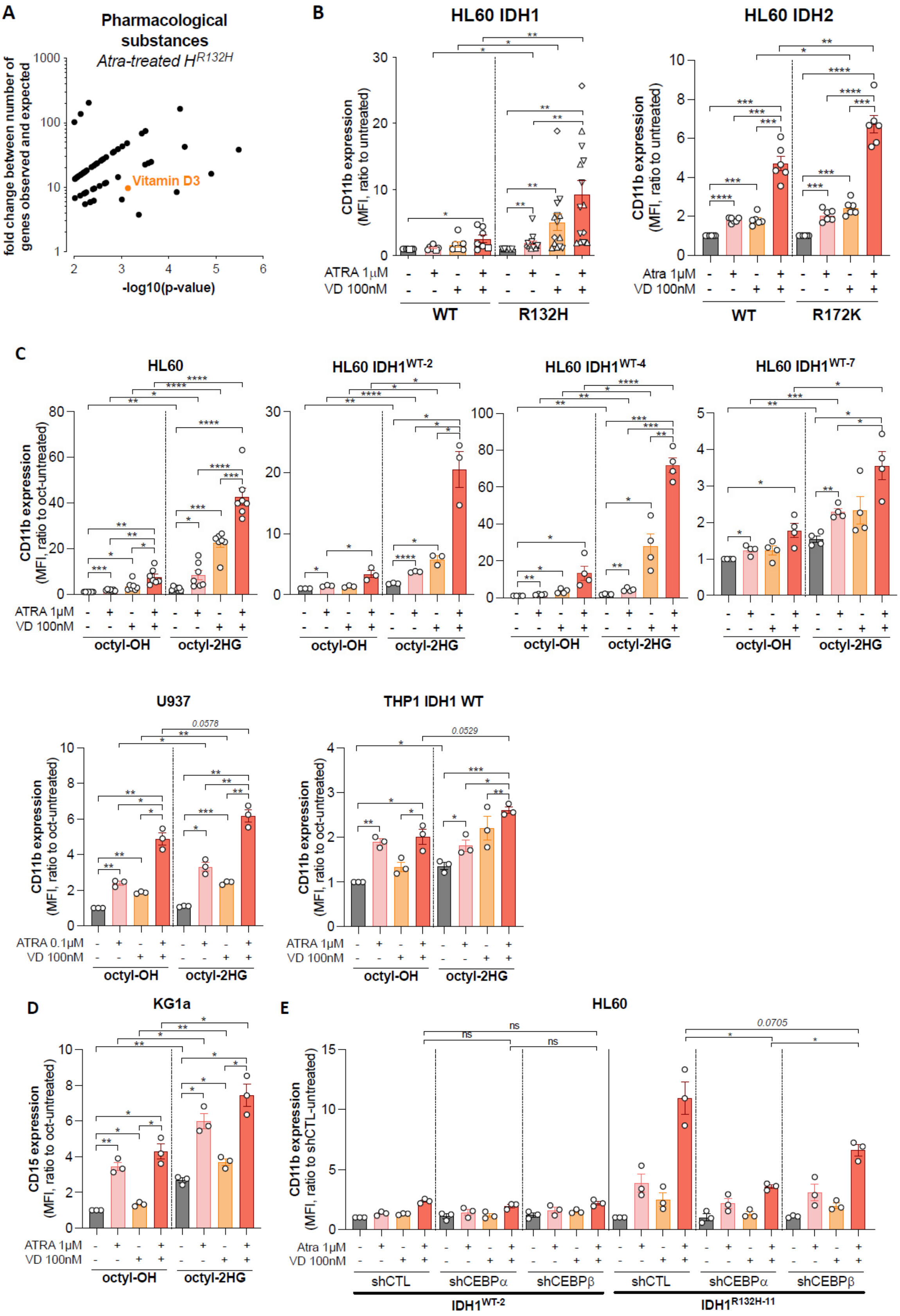

Figure S4. Targeting vitamin D receptor pathway enhances anti-AML effect with ATRA in a CEBPα-dependent manner. (**A**) Pharmacological substances (Genomatix) enriched in gene signature of ATRA-treated HL60 IDH1^{R132H} (H^{R132H}) (**B**) CD11b expression (MFI) measured by flow cytometry in

HL60 IDH1^{WT} (clone 2: ○, clone 7: □) versus HL60 IDH1R132H (clone 5: ◇, clone 11 GFPLOW: △, clone 11 GFPHIGH: ▽) and in HL60 IDH2^{WT} versus HL60 IDH2^{R172K} treated for 3 days with ATRA (1 μM) and VD (100 nM) alone or in combination. (**C**) CD11b expression (MFI, ratio to untreated) measured by flow cytometry in 2HG-treated (100(U937)-200μM for 1week) HL60 IDH1WT-2, HL60 IDH1WT-4, HL60 IDH1WT-7, HL60, U937 and THP1 treated for 3 days with ATRA (0.1μM for U937, 1μM for others) or VD (100nM) alone or in combination. (**D**) CD15 expression (MFI, ratio to untreated) measured by flow cytometry in 2HG-treated (200μM for 1week) KG1a treated for 3 days with ATRA (0.1μM for U937, 1 μM for others) or VD (100nM) alone or in combination. (**E**) CD11b expression (MFI) measured by flow cytometry in HL60 IDH1^{WT-2} shCTL vs. shCEBPα vs. shCEBPβ and in HL60 IDH1R132H-11 shCTL vs. shCEBPα vs. shCEBPβ treated for 3 days with ATRA (1 μM) and VD (100 nM) alone or in combination. *: <0.05; **: <0.01, ***: <0.005; ****: <0.001.

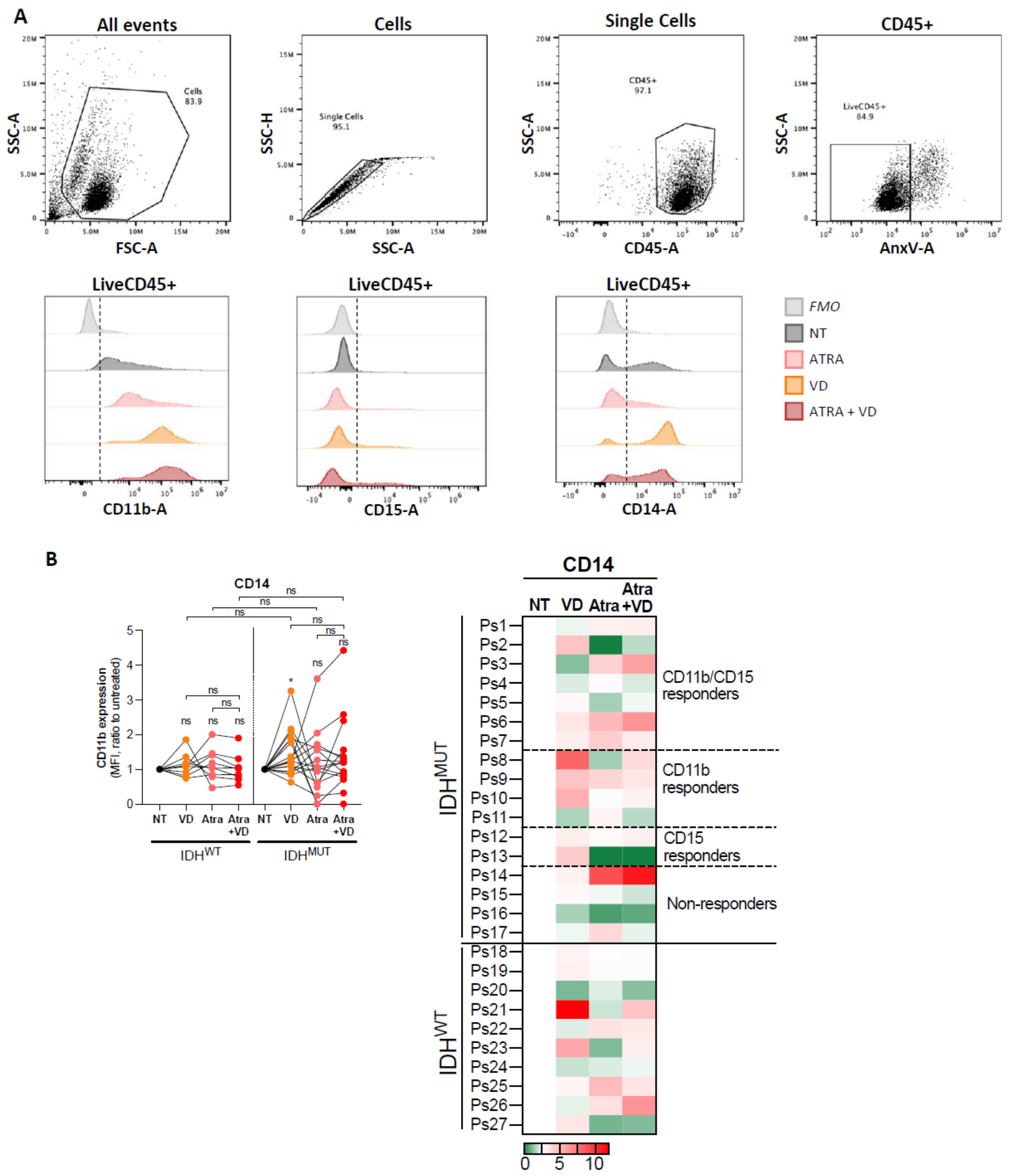

Figure S5. Targeting vitamin D receptor pathway enhances anti-AML effect with ATRA in IDH^{MUT} AML patients. (**A**) Representative gating strategy to measure CD11b, CD15 and CD14 expressions by flow cytometry in Ps8 treated with ATRA and VD alone or in combination. (**B**) CD14 expression (mean fluorescence intensity, MFI) measured by flow cytometry in IDH^{MUT} versus IDH^{WT} patients treated with ATRA and VD alone or in combination (left panel). Results for each patient are represented individually (right panel).

Figure S2A

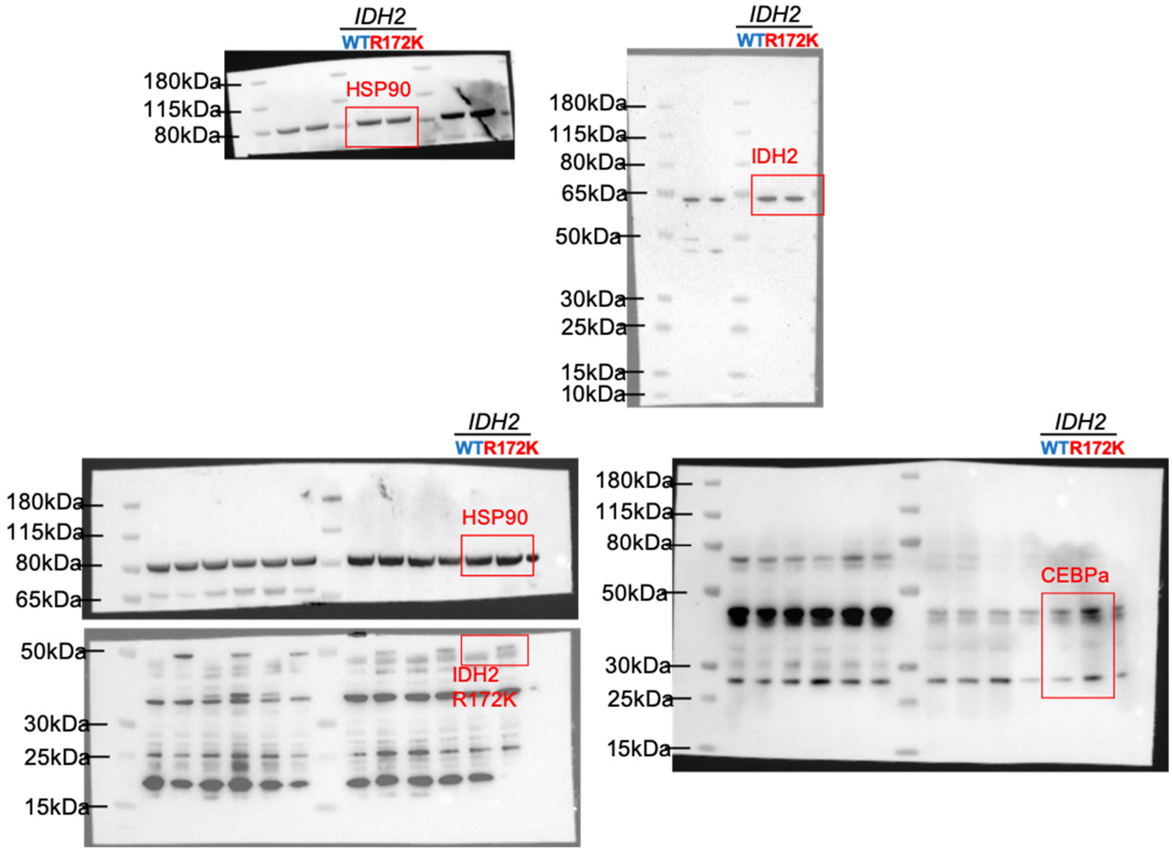
IDH2
WTR172K
180kDa
115kDa
80kDa
HSP90
IDH2
WTR172K
180kDa
115kDa
80kDa
65kDa
50kDa
30kDa
25kDa
15kDa
10kDa
IDH2
IDH2
WTR172K
180kDa
115kDa
80kDa
65kDa
HSP90
50kDa
IDH2
R172K
30kDa
25kDa
15kDa
IDH2
WTR172K
180kDa
115kDa
80kDa
50kDa
CEBPa
30kDa
25kDa
15kDa

Figure 2B

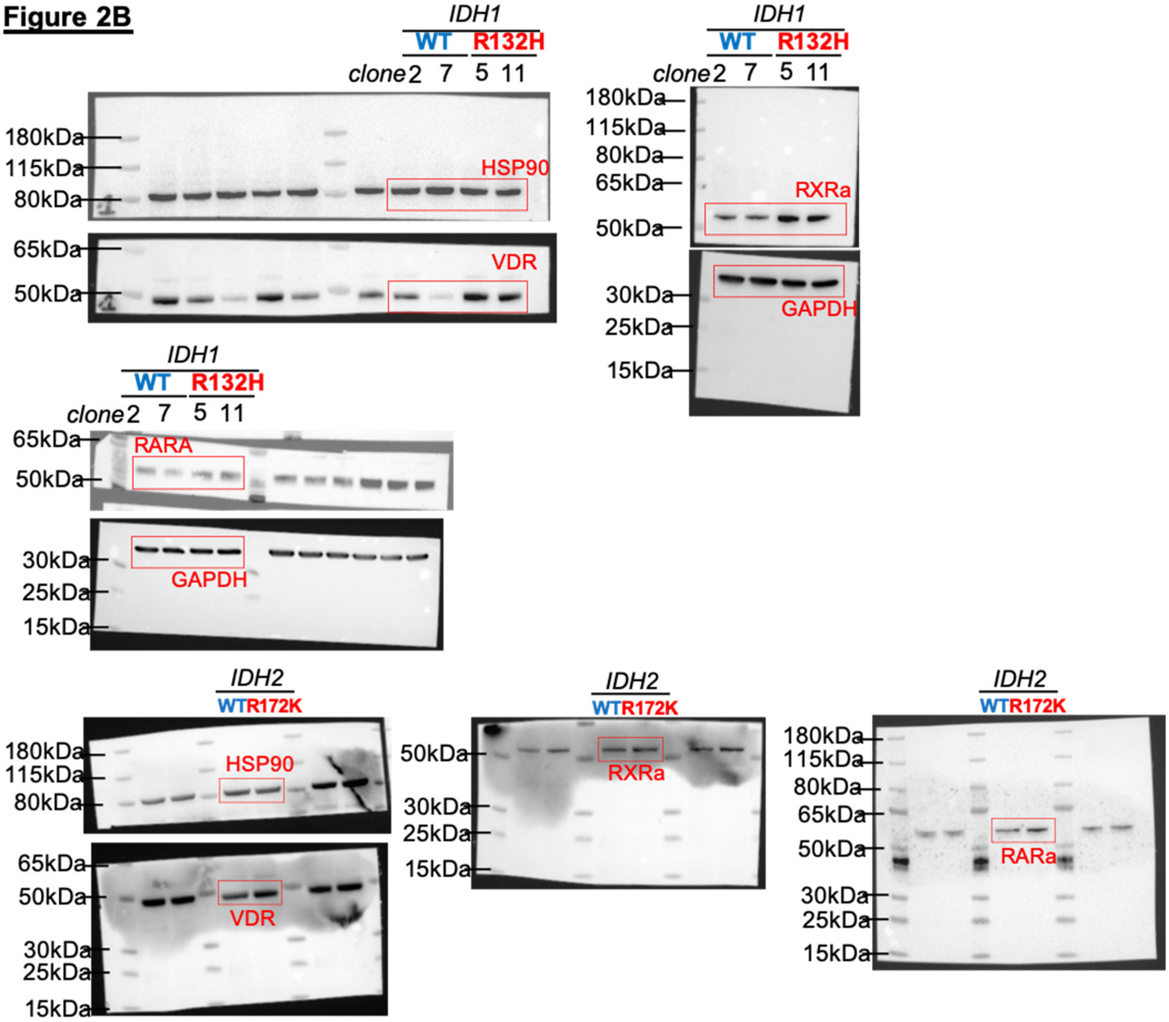

Figure 2D

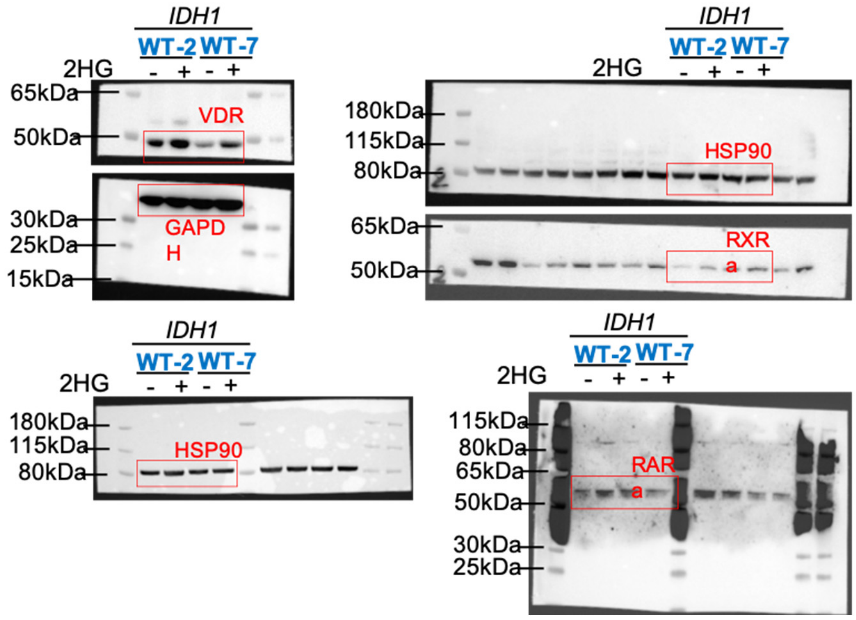

Figure 2F

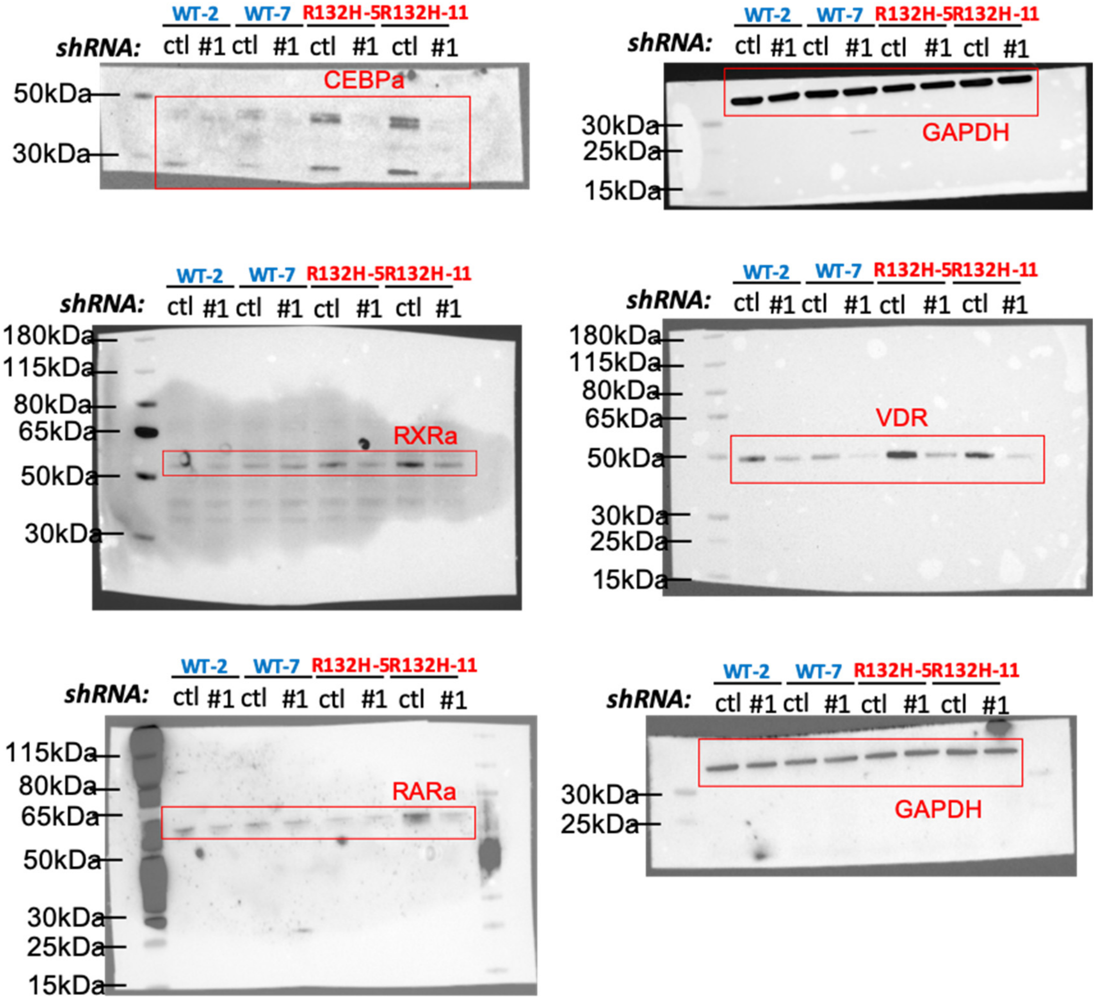

Figure 2G

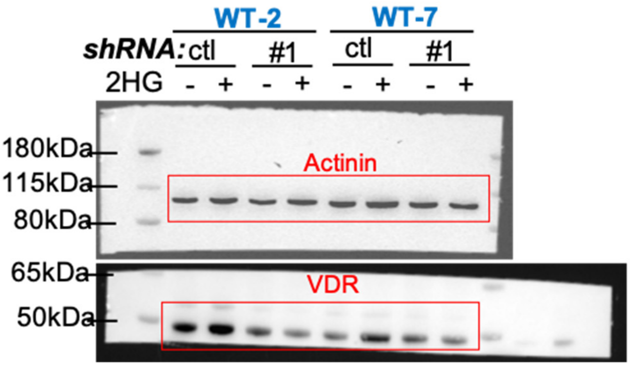

Figure S3A

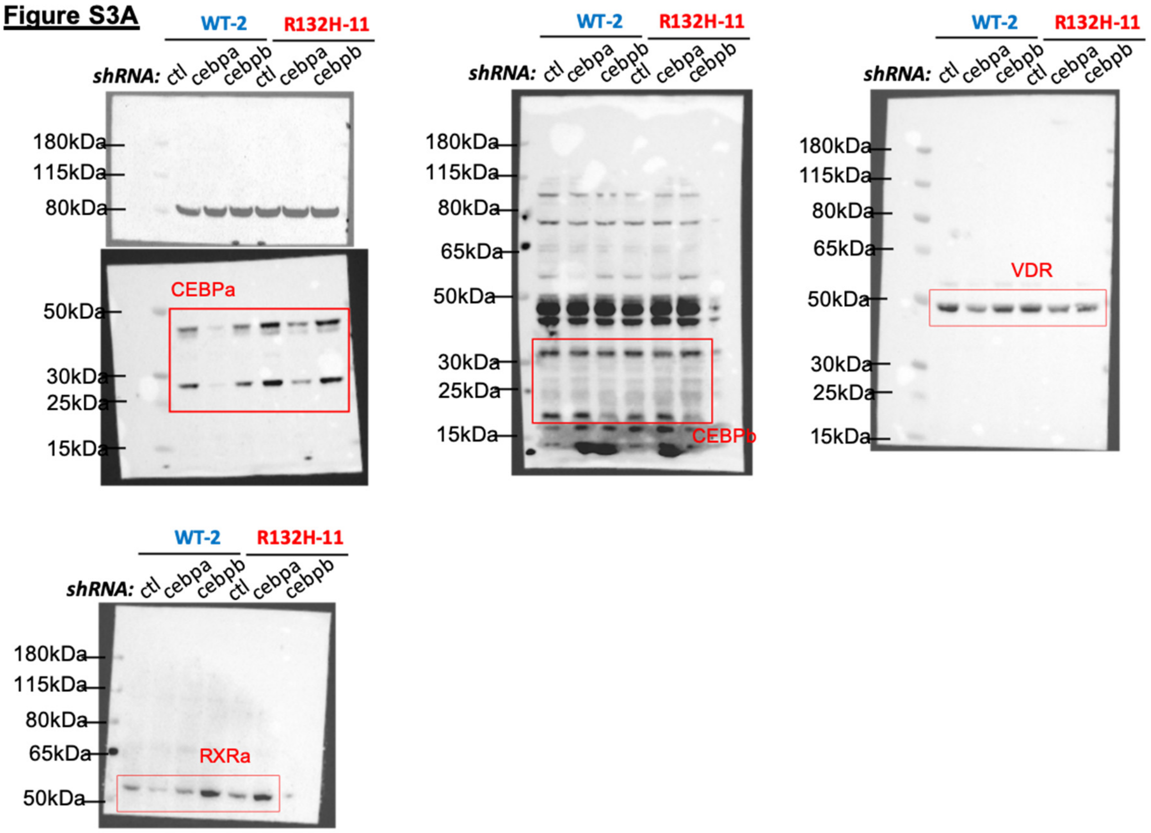

Figure S3B

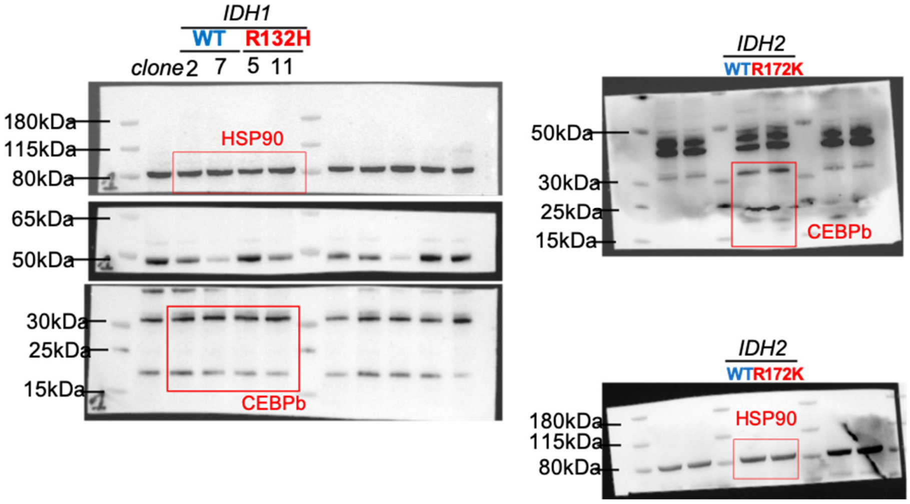

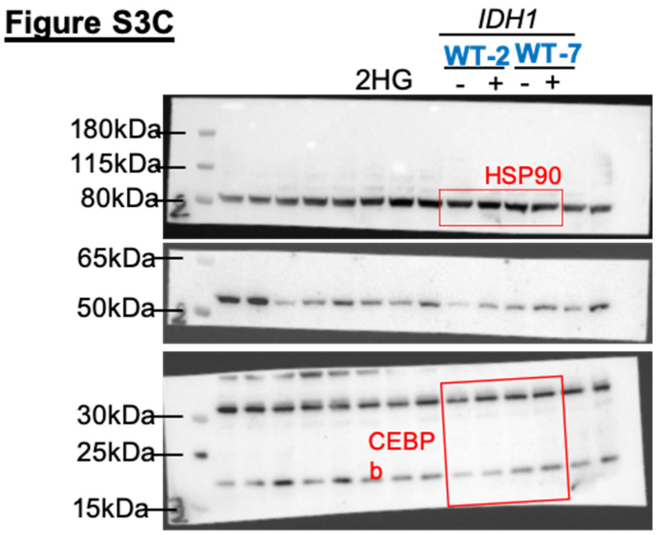

Figure S6. Uncropped western blot images.

© 2021 by the authors. Licensee MDPI, Basel, Switzerland. This article is an open access article distributed under the terms and conditions of the Creative Commons Attribution (CC BY) license (http://creativecommons.org/licenses/by/4.0/).
