# Supplementary material for: Activation of Vitamin D Receptor Pathway Enhances Differentiating Capacity in Acute Myeloid Leukemia with Isocitrate Dehydrogenase Mutations
Source: Cancers (Basel). 2021 Oct 19;13(20):5243. doi: 10.3390/cancers13205243 (PMC8533831; doi:10.3390/cancers13205243)
Supplement: Supplementary file 1 [file cancers-13-05243-s001.zip › Table S1.pdf]

Table S1 : Gene signature (related to Figure 1A)

HL60^{MUT} (Boutzen et al., 2016)		HL60^{WT} +2HG (Boutzen et al., 2016)	IDH^{MUT} patient (TCGA)			IDH^{MUT} patient (Verhaak)
Upregulated and Downregulated (>+/-1.5-fold change) genes in HL60 IDH1-R132H to WT IDH1 (Boutzen et al., 2016)	*Downregulated (>+/-0.667-fold change et pv<0.05) genes in HL60 IDH1-R132H to WT IDH1 (Boutzen et al., 2016)*	*ATRA responsive genes in WT+2HG (FDR<0.05) (Boutzen et al., 2016)*	*Upregulated and Downregulated (>+/- 1.5-fold change et pv<0.05) genes in mutant IDH to WT IDH*	*Downregulated (>+/- 0.667-fold change et pv<0.05) genes in mutant IDH1 to WT IDH*		*Upregulated and Downregulated (>+/- 1.5-fold change et pv<0.05) genes in mutant IDH to WT IDH*
CCNA1	CLECL1	XK	CYP27C1	CARNS1	CD36	ELL2
NFATC2	ZNF429	VIM-AS1	STL	GPR183	CX3CR1	SELL
MNDA	ZNF431	TYROBP	ATP1A3	RBM47	SCPEP1	DNM1
PRTN3	IGLJ3	TTLL11-IT1	CYMP	TDO2	CRYGB	CDK3
CPT1A	GPR174	TRIB1	ZFP112	CTXN1	KIR2DL4	GAB2
SLCO4C1	ZNF551	TNRC6C-AS1	NMT2	NPBWR2	ARSG	AMY1A /// AMY1B ///
IL8	ZNF680	TNFAIP2	NCRNA00246B	SPAG6	PECAM1	SSBP2
DSG2	ZNF714	TLR3	DOC2A	HCRTR2	CPE	MEIS1
SCPEP1	GALNT3	THSD7A	PGAP1	LOC100129935	PNPLA1	TRIM73
LYZ	IGLL1	THEMIS2	OBSL1	PHKG1	GHRHR	PBX3
GLIPR1	T1560	TCN1	ABCA3	GAS2L3	CXorf21	STAR
ANPEP	ZNF350	SUCNR1	OR2L13	OTC	DOCK9	MARCKSL1
CCR7	CRIM1	SREBF1	FLRT3	M41	SIGLEC9	FOSL2
CD68	SH2D4A	SQRDL	CCDC102A	IFI30	C3orf47	RGC32
ACSS2	FSTL5	SPRY1	F3	LOC100128098	KCNK6	C1ORF186
PTGR1	ZNF83	SPECC1	COL4A5	CDKN1A	CXCL16	LOC286052
TNF	CLC	SMOX	C14orf79	NPPA	SLC28A2	ARL4A
CEBPA	ZNF675	SMIM3	LOC400891	LOC400084	AMICA1	PRICKLE1
TRIM71	GPR65	SLC44A1	PTGR2	KCTD21	C16orf3	USP44
DHRS9	ZNF544	SLC16A9	INHBE	PLBD1	CCDC144C	HOXA4
TNFAIP3	ZNF530	SLA	INSL3	FSTL5	SP8	SLC35F2
PTPRJ	ZNF790	SIGLEC5	PRKD3	GAFA1	ITGAM	DACH1
P2RX7	ZNF850	SERPINB8	MAK	PTPRK	PDE7B	BTBD11
JUP	SH3BGRL2	SERPINB2	H2BFM	FAM9C	SH2B3	SNCAIP
CTSG	ZNF814	SERPINB10	ST7OT1	CD86	SLC7A7	ADAM17
MS4A3	ZNF260	SERPINB1	DOCK4	WDR16	HLA-DMA	PELI2
LTBR	CST7	SELP	COL24A1	LOC100130468	GPM6B	HOXB2
RET	MIR181B2	SELL	ZBTB16	FBXL2	PTPRJ	MIRN21
FCGR1A	IL1RL1	SCUBE1	CPA3	CEACAM6	SCG5	NXF3
CD300A	TCF4	SCPEP1	SLC16A9	SOX14	APCDD1L	GPC4
AZU1	YME1L1	SAT1	MYCN	RBP1	QRFPR	DPYSL3
LGALS1	ZNF550	RXRA	HOXA9	SLC30A2	LOC401324	HOXA10
KLF6	ANXA3	ROGDI	GOLGA8B	LOC100505832	HIST1H2BK	TMEM49
GBP2	SMIM10	ROCK1P1	HTRA3	UTS2	CAPN12	FAM46A

APOBEC3G	RAB27B	RNU5B-1	SCGB2A2	ELTD1	LOC440900	NFIL3
APOBEC3D	NFE2L3	RCSD1	WDFY3	RASGEF1A	CLECL1	SLC9A3
NRP1	HEG1	RALGAPA2	CDH2	DSCAML1	WFDC1	TES
JUN	CEP70	RAB31	LOC644538	MCOLN2	LOC100505633	IRX5
ME1	MIR3198-1	PVRL1	ANKRD33B	LOC388210	CARD18	FUT4
TSHZ3	CD38	PTGS1	LRP12	SEPX1	RNF148	PGAP1
METTL7A	TRAJ36	PSMB10	FLJ41455	SP5	HSPC159	ATP2B1
PREX1	HPSE	PRTN3	SH3BGRL2	UPP1	MST1P9	RASAL2
AHNAK	AAK1	PRKCB	LHFP	LOC642345	CD33	FGFRL1
STEAP3	EXOC6B	PRG2	SLC9A7	GLRX	CD74	CFD
MIR33B	CBX5	PRAM1	PABPC4L	ODZ4	LOC730755	AK5
ZNF385A	FAM134B	PPP1R18	KIT	CD1B	SERPINI2	CHD7
STAR	ZNF430	PLEK	LRRC56	MAS1L	DENND2D	AZU1
TYROBP	XYLT1	PLCB2	MLLT11	ANXA13	PART1	LOC285148
SERPINB1	ZNF708	PIWIL4	MFI2	LOC100128946	GIMAP8	HNRPLL
SESN3	TRAJ38	PARP14	TWISTNB	BCL2A1	MGC42157	SKIL
ITGA6	TFPI	OSM	CBX2	SLITRK1	PLEKHA7	JAG1
PLXNB2	ZNF345	OAS3	CRISPLD1	ALS2CR12	CES1	HOXB6
CFD	GPR27	NOG	GRAMD1C	SLC25A18	MYBPH	LOC652226
TNFAIP2	ANXA1	NFE2	RASAL2	RBM20	MYCBPAP	COL4A5
TEX15	ANTXR1	NDRG1	KISS1R	ABRA	EFHD1	HOXA3
CD300C	ZNF721	NCF2	KHDRBS3	ITIH3	SIGLEC5	NRXN2
TNFRSF10D	EML5	NCF1C	CLDN10	RNASE3	KAZALD1	RFX2
TSPAN14	DACH1	NCF1B	CCDC122	ARSJ	TEKT3	HOXA5
MS4A7	SLC22A16	MOV10	SERPINH1	WDR87	TNFRSF11A	DSC2
STXBP5L	ZDHHC23	MIR548D1	TRAT1	LOC100506495	REXO1L1	HOXB3
LPPR3	IGLJ7	MIR181B1	PON2	THBD	AGXT	MXD1
YME1L1	TRBV5-4	MICAL2	TFPI	TKTL1	SLC26A3	LOC643911 /// LOC650
CLEC12A	ZNF566	METTL7A	NRXN2	DKFZP434F142	CD300LF	CTSG
VIM	UBXN10	MCEMP1	CHST13	PLA2G10	ARHGAP39	NKX2-3
MIR223	PDE7B	LOC100506459	RAB9B	SPIB	PRG3	S100A10
PCED1B-AS1	EPX	LINC00649	C19orf77	DNTT	SLC44A3	CD9
PTGS1	CCDC141	LFNG	C1orf186	VNN1	HDAC9	HOXA9
PRSS57	MIR361	LBR	RBP4	GNPDA1	OSTalpha	MPO
EMILIN2	TRBV6-6	KRTAP9-3	ZNF324	ABCC6	CNTN4	CCNA1
CMTM7	TRAJ45	KDM7A	LOC154761	BEAN1	GCNT2	HOMER3
HGF	CYP4V2	ITGB7	HDGFRP3	ALDH4A1	TPK1	MGC33556
CCL3L3	LUM	ITGAX	OSM	LOC253044	LOC285778	NEGR1
KLF7	MIR4521	ITGAM	ENO2	TULP1	GPRIN3	MYCN
LILRA2	KRCC1	ITGA5	EGR1	KIAA0825	C20orf186	TPSAB1 /// TPSB2 ///
ARMCX6	IFITM1	IRF1	NPM2	LILRB3	XCR1	TPSAB1
CR1	TRAJ34	IL6R	FAM57A	LOC283033	CD1A	EREG
DDIT4	SESTD1	IL10RB-AS1	DPY19L2	IL1F8	C1orf116	RASGRP3
ALDOC	NLRC3	IER3	NUDT9P1	UCP2	CCDC153	MAMDC2

IL6ST	IL5RA	ICAM1	EMID1	RPS6KL1	PLIN4	CA2
APBB1IP	IFITM2	HOXA1	DPYSL3	C1orf49	OR4C1P	IRX3
METTL7B	KRT6C	HNRNPLL	LOC401097	ARHGAP31	FBXO6	CCDC3
ARNTL	CLDN12	HLA-L	CD59	CTTNBP2	ARHGEF10L	BEX1
FKBP3	CGREF1	HIC1	HMBOX1	C4orf34	NAGK	
HVCN1	ACTR3B	HGF	SCML1	SAMD4A	EHD3	
GPR84	FAM46C	GZMA	TANC2	GIMAP7	FCER1A	
BPI	SASH3	GVINP1	SMAD1	ADCY8	LIPJ	
CXCL10	LNP1	GRN	CENPV	EPAS1	BANK1	
FOSL2	TMEM14A	GPR174	SEPP1	CLRN3	RAB31	
LYST	CSTF2T	GPR132	ZNF711	CDH5	TBX15	
IL6R	MYH15	GNG2	LRRN1	KCNJ11	DLL4	
THEMIS2	ZNF573	GBP3	CCDC3	C6orf138	PLAC4	
DDIT3	HES1	GBP2	DNM1	DKK2	ANXA2	
MTL5	SAMD9L	GALM	HMGN5	NLRC4	LOC728342	
NFE2	UBE2E3	FLOT1	ARHGAP5	LHX6	C21orf56	
ARRB1	PLCH1	FGR	ATP6AP1L	SLC38A4	TLCD1	
SGPP1	ZNF613	FGFBP3	MAP1A	SNX31	RIT2	
TET1	TIGD2	FBP1	CDK14	HIST1H4D	SERPINB8	
IFI30	IGFBP2	FAM46C	USP53	ALOX5	HLA-DQA1	
PSTPIP2	TRAJ56	F5	IGF1R	TNF	SLC18A3	
ELANE	FAM171B	ERAP2	FLJ41484	XK	ATP4A	
HSPA1B	FAHD2B	EPX	C1orf21	FAM9B	LOC284669	
EMR2	NCALD	EPAS1	PP13439	IFNK	CIB2	
C5AR1	ZNF8	EMILIN2	SERPINE1	LOC100505964	LY9	
SSBP3	ZDHHC14	ELF4	CYP11A1	HOXB1	KLK15	
VCL	PCSK6	EDIL3	C5orf30	NAT8	CD1D	
PXN	CABLES1	DTX3L	LOC100127983	TIMP1	FGGY	
JHDM1D	RIMKLB	DOCK5	ANKRD37	CA3	AGTRAP	
PTGER4	MIR563	DNAJB12	LOC283861	KLRF1	LOC729059	
LDLRAD3	MIR29C	DHRS9	TRIM6	PHYHD1	LOC100506655	
RAB39A	septin 11	DHRS3	LOC100101266	LOC285972	IL1F10	
ANKRD22	CYB5A	DEFB115	GPHN	CD68	CALML4	
SLAMF6	THSD7A	DDX60	FAM135A	LONRF2	HORMAD1	
ARMCX3	TFR2	DDIT4	LOC100506451	HTR3B	C1orf65	
PVR	PROK2	CYTH4	AASS	LOC497256	ZNF718	
S100A13	KBTBD7	CYBB	TMEM99	ASB2	OPLAH	
FHL3	SLC9A7	CTSO	ZNF880	VLDLR	LOC285419	
KITLG	ZNF264	CLEC12A	FAM89A	FGD4	OGFRL1	
LMNA	YME1L1	CFP	TLR3	CLEC1B	KCTD12	
TRAM1	DBP	CELSR3-AS1	GPSM1	CD19	GRASPOS	
RXRA	TESPA1	CEBPA	ZNF257	C17orf44	GRIP2	
CD86	EXPH5	CD53	MAP7	SYTL5	ZIC3	
OAS3	WDR17	CD38	PHGDH	NCAN	C2orf55	

ILDR2	CD300LB	ZBTB10	LOC439938	VIPR2
RNFT1	CD300A	ZNF251	LRG1	MPZL3
ENTPD1	CCR1	LOC400986	FIBCD1	NPFFR2
HLA-C	CCNA1	CLGN	MGC87042	IFIT5
DNM1	CCL20	LOC100506010	SERPINB2	CD80
TMBIM1	CCL2	DSC2	KCTD7	TNFSF13B
EVI2B	CARD6	ZNF578	ZRSR1	LOC339260
SLC25A6	C1orf106	AUTS2	GIMAP4	MGMT
STXBP6	C16orf54	ATP1B1	C10orf44	NEDD9
SLC16A3	C10orf54	PROK2	MARCO	NTM
ZNF611	ARL14EPL	ZNF677	LOC100505478	MARCH1
MARCH3	ARHGAP9	STMN3	HAP1	P2RY13
METRNL	ARHGAP12	C15orf52	BEST2	C5orf32
RBFOX2	APOL6	XYLT1	LRP3	NAV1
TRIM69	APBB1IP	SRSF12	LOC780529	CCL24
CSF3R	AP1S2	CCRL1	FAT4	KIAA1239
HPCAL1	ALOX5AP	SMO	C10orf116	LOC100287628
TUBA1A	ADGRG5	SLAIN1	OR2J3	TYROBP
PTGER2	ADGRG3	RHOBTB1	PYHIN1	LY96
FCGR1B	ACSL1	FLJ45482	FAM53A	AVPR1B
GGT5	ACPP	GPR125	OR1J4	CCDC65
CYTH4		C21orf96	VIT	TDRD12
ITGAX		DST	CHST15	MYPN
METTL23		LOC100128252	XPNPEP2	BHMT2
LACTB		ZNF493	DEFB125	IL10RA
CD82		LOC388692	LOC100129029	GLTPD2
PNRC1		ITGAV	LOC348751	CALD1
ST6GALNAC6		ZNF219	MICAL2	NOD2
RNASE6		NCRNA00260	FCRLB	LOC283484
TSSK3		CD99P1	HSD17B3	UNC93B1
RETN		PAWR	BASE	ZSCAN12P1
BNIP3		ANGPT1	OR1A2	LOC100293532
TBC1D30		AK4	CLEC4E	C19orf59
RASA4		NPDC1	C20orf85	LOC100506714
COIL		PHOSPHO2	GNAT2	LOC388948
EHBP1L1		MANEAL	PDE4A	MOGAT1
GPR114		TCEA2	APOBEC3A	KIAA0087
MRAP2		SLITRK4	C14orf86	ZFHX3
MED13L		ABCA2	NXPH2	CDH7
HK1		ZNF667	OR10D3	COL8A2
MS4A6A		SEMA4C	POU3F3	SPRR2G
LILRB1		H2BFXP	ARMC9	TLR8
CD244		LOC100505687	FAM26D	PDZD3
GLO1		RNF217	SIGLEC15	C5orf47

TM6SF1		GPR162	GJA10	TMEM150B	
SLC2A3		CPNE8	OR4D2	GLDC	
TLR6		MCHR2	HTATIP2	SLC22A2	
WIPI1		C11orf95	PRLR	CAPN9	
KLF10		DCBLD2	LOC149703	PRTN3	
RCAN3		CHST12	LILRB2	TEAD2	
SLCO3A1		LOC727916	NCALD	TRIM10	
ITGA5		LOC100507266	NAPSB	MIPOL1	
TSC22D3		UCN	CCDC64B	APOC2	
PARP12		TMSB15B	SGMS2	GAGE3	
FURIN		GPR27	KNG1	ACOT4	
APOBEC3F		P2RY11	CLCN1	FAM82B	
MIR3161		BAG2	FBXL21	EPB41L2	
BCL6		LOC100272216	POU5F1P3	FBP1	
TXNIP		C14orf128	MEIG1	GCET2	
NOG		ZCCHC18	SLC7A13	GNAL	
STAT6		SPNS2	TAS2R41	FPR3	
SESN2		C15orf5	SLAMF7	KBTBD8	
HSPA1A		ZNF395	NLRP2	NCF4	
IL13RA1		NGFRAP1	CSTA	RGS10	
TLE4		MMP2	EME2	SLC25A41	
TP53BP2		LAMB2P1	HK3	SIGLEC7	
ZEB1		KIAA1683	GRM2	SNX21	
SSH3		CHD7	PAX7	HOXC13	
PPP1R26		NOXA1	NCR1	HIST1H3I	
MIR520F		PKIA	LOC100506795	SLIT1	
DNMBP		ADARB1	PRICKLE3	MS4A2	
NLRC4		WBP5	RHBDL2	LOC100216479	
P2RY2		SMAD5	C16orf89	LRP1	
UBL3		FGFRL1	PRLHR	CASQ1	
LTA4H		JMY	LGALS13	KRT78	
GBGT1		NCRNA00173	PKIB	LRRC32	
SLC39A14		TMEM22	TAF7L	BCL2L10	
QPRT		CXXC5	ASGR1	HTR3A	
ICAM1		LOC100288152	VGLL3	LOXL3	
ADD2			GAS1	SLC44A5	
PLOD1			ARL5C	LPPR5	
DENND5A			DNAJC15	TSPAN16	
ITGAM			LRRC18	CALCB	
TNFRSF10B			JOSD2	SAMHD1	
			CYBB	FAS-AS1	
			ZFYVE28	PHLDB2	
			ISG15	TFR2	
			ARMC3	AGR2	

			SIGLEC16	ZBTB38	
			C3orf43	C14orf184	
			CFP	PLTP	
			CD14	ZNF536	
			C17orf46	IL1RL2	
			IRG1	C21orf116	
			KRT12	FLJ42709	
			TLR5	FCGR2A	
			RIMS1	C5orf20	
			LAIR2	ACOX2	
			PANK1	CECR1	
			AQP9	SLC6A12	
			KRT80	NFAM1	
			GPR65	HERC2P3	
			FCAR	CD1C	
			SPEM1	SLC2A12	
			OR2F1	ALOX5AP	
			AZU1	NOTCH4	
			LOC339535	DAP	
			KCNA5	EPHB3	
			HLA-DRB6	QPCT	
			MYCL1	GPR68	
			NTN4	MX1	
			AKR1B1	FCER1G	
			KLF5	CD101	
			MYL1	LOC643529	
			STATH	DOCK10	
			DUOX2	TREM2	
			CHRM1	LOC730091	
			SLC37A2	PDPN	
			HSD17B6	CSN1S1	
			VNN2	GCHFR	
			RNASE2	LRRIQ3	
			LOC151174	HLA-DPB2	
			RRAD	SSTR4	
			SLC34A1	ZNF518B	
			CCR4	MS4A4A	
			COL4A1	IL12RB1	
			LOC100287114	FLJ32790	
			DGAT2	ST7OT4	
			LHFPL1	DHRS9	
			CYP51A1	A2LD1	
			CRNN	GP1BA	
			ATP10A	GLB1L	

			HAVCR1	DKFZP761C1711		
			PTGDS	THBS4		
			LOC100507244	TRIM72		
			FCN3	PRDM13		
			SLC26A9	TMEM130		
			CLEC4D	RTP4		
			MGAM	C12orf5		
			AXDND1	ACAT1		
			LACRT	LYVE1		
			CYP2C8	GRIN2A		
			CCL17	LRP1B		
			OSCAR	CTSS		
			C17orf59	MS4A14		
			LOC254099	KCNN3		
			CRYBA1	CRB1		
			KRTAP10-11	LPAL2		
			STEAP4	PDLIM1		
			ZSCAN4	LILRB1		
			CDO1	HLA-DPB1		
			VGF	LOC386597		
			C21orf128	HBZ		
			CHST7	IL5RA		
			TFPI2	HVCN1		
			GADL1	GKN2		
			CXADR	HLF		
			CLEC7A	ENC1		
			LOC100133790	LOXHD1		
			ALDH2	IL2RG		
			FCHO1	TMEM47		
			LOC100132116	GALC		
			EPHX1	PNP		
			GIMAP6	LPAR6		
			OR7E24	ABCA9		
			ST5	FRMD3		
			C6orf58	SLC22A23		
			S100A9	HAVCR2		
			CDH18	NLRP12		
			KIAA0930	LOC284865		
			SLC41A1	BST1		
			PRAMEF10	EDNRA		
			CLEC4F	WNK2		
			OVOL2	C1orf53		
			RPL39L	PSRC1		
			MARCH2	RFPL1		

			HCP5	PIK3R5	
			NEFM	C20orf103	
			CYP46A1	FAM19A4	
			ATP1A2	CACNG4	
			NHSL2	MGC2848	
			KLF4	ADAP2	
			KIAA1274	SLFN13	
			LOC729970	TMC4	
			RBM11	SLC26A7	
			C2orf46	TMEM144	
			ABCG2	CD200R1	
			KIR3DL3	CDHR3	
			LOC644714	LOC100131262	
			AFF3	BPIL2	
			C15orf56	CD1E	
			TAC4	TSHZ3	
			CR2	C1orf170	
			ALB	SMPD3	
			PCDHB16	MECOM	
			CLEC1A	CUX2	
			ROBO1	SH2D1A	
			LOC100128343	SH3D21	
			ATP6V0D1	MEGF9	
			HCG11	SLC2A8	
			NPC1L1	GAPDHS	
			EVC	GRID2	
			HES6	CSF2RA	
			SEMA3C	LOC286121	
			LOC728192	NCF2	
			PNLIPRP3	LST1	
			ASAH2B	DOK2	
			BTBD17	ZNF185	
			INSM2	HEXIM2	
			IER3	CLEC12B	
			CLDN6	BLNK	
			KANK4	RIBC2	
			POM121L12	CDC42BPB	
			UMODL1	GPR15	
			FLJ35024	TEAD1	
			PAH	RNASE1	
			LOC100507391	C1orf46	
			TMEM145	FCGR2B	
			ARSI	C19orf26	
			HTR6	UGT8	

			CDH13	MICAL1	
			CYBA	PRSS47	
			ZNF703	KIF16B	
			LOC100287030	NUDT19	
			APOF	C7orf57	
			HIST1H2AL	DPPA4	
			LOC728114	APOL4	
			PEBP4	SRL	
			GPRC5A	LOC151438	
			TTLL13	FAM105A	
			TRIM29	PAK6	
			C2orf3	SLC24A4	
			AKAP5	DYSF	
			LOC728805	LOC100505826	
			ADORA3	AKAP7	
			MAG	IFI44	
			MYBPC2	SERPIND1	
			ASPA	CSF3	
			PADI4	KRT35	
			CYP2C18	BTNL8	
			LOC645323	PIH1D2	
			PCDHGA9	LOC284788	
			LOC100131496	TCEANC2	
			NOS1AP	MYOF	
			HPR	NPL	
			ALMS1P	PFKFB2	
			RBP7	PRAME	
			GDAP1L1	OR7E19P	
			SCGB2A1	ZNF385A	
			RPL10L	TPPP2	
			OR2J2	UBASH3B	
			SHC3	CD300C	
			PADI2	ZNF684	
			DBH	NEK6	
			C19orf47	HNF1B	
			SGSH	LIPA	
			S100A8	KCNB2	
			HOXC6	TUBA4A	
			GPRIN1	NAAA	
			FCGR3B	DENND2A	
			HOXD8	FLJ25328	
			MEOX2	SNX29	
			SLC11A1	SERPINF1	
			MS4A7	INTS4L1	

			LPXN	LOC100129794	
			CNTNAP1	SPATA12	
			GNS	VSTM1	
			VSIG4	SIGLEC11	
			HLA-DPA1	DHRS3	
			C2	STARD8	
			MMP13	ATP10B	
			DRP2	CMTM2	
			KIAA1755	CMKLR1	
			GCH1	RNF175	
			TMEM200A	CD300LB	
			GIMAP1	RASSF4	
			CLC	CLTCL1	
			ODF4	RAPGEF5	
			B3GALT4	S100P	
			HIC1	MMEL1	
			AIM1	CR1	
			SYCN	LOC200772	
			DKFZp547J222	IL1RN	
			PNOC	GPBAR1	
			BASP1	LOC100287547	
			CCDC19	WFDC6	
			C15orf55	NLRP14	
			DUSP9	P2RY6	
			ADIG	FCGRT	
			LPCAT1	BRSK1	
			S100A1	C3orf45	
			PDC	FERD3L	
			SH3PXD2A	ZNF167	
			ARC	PP14571	
			TRPM8	EPOR	
			OR5P3	FLJ20712	
			ST14	RNH1	
			GLT1D1	FCGR2C	
			HNMT	ERCC2	
			ZNF705G	PTPRT	
			LOC400238	MRPS15	
			CALHM3	LOC100505967	
			LOC100129058	AIG1	
			CC2D2A	F5	
			MAGEB18	C10orf128	
			STAB1	ANKRD53	
			NUDT16P1	RHBDL3	
			LRRC29	CTSH	

			PACRG	C17orf51	
			SLITRK2	MPEG1	
			EDDM3B	ACER1	
			DOK3	CDH23	
			PILRA	MLF1	
			SLC26A4	KAZN	
			PRSS50	CCR2	
			GPR158	CXCL9	
			LOC100130452	ZFP90	
			VCX2	TMEM104	
			LILRA6	PALM3	
			ANKRD45	PTPRE	
			ZFR2	LOC338667	
			NLRC5	ADAMTS16	
			CNN3	CIDEB	
			NCRNA00189	TEX13A	
			FGR	GIPC2	
			LOC285857	AP1S3	
			C16orf81	NEB	
			HLA-DOA	PPARD	
			CFHR4	SPINT2	
			OR12D2	SIDT2	
			DIO3	CEBPE	
			STAB2	ENHO	
			MIA2	CARD16	
			SLCO5A1	ILDR1	
			CREB5	HIVEP3	
			PEG3-AS1	LAX1	
			GRN	TLR10	
			SUCLG2	ESPN	
			DAPL1	ADPRH	
			USP18	HENMT1	
			ANKRD30A	CACNA2D3	
			MDGA2	FAP	
			OR2L1P	LGALS3BP	
			KLHDC7B	C12orf74	
			ANXA2P2	RFX6	
			NPBWR1	C20orf27	
			ZIC4	SLAMF6	
			C7orf10	KIAA0319	
			CD4	DLGAP1	
			RXRA	KLK14	
			SLC5A12	ENTPD3	
			IL23R	S100B	

			IFIT1	SLC30A1	
			SERPINB9	NRP1	
			SCGB1A1	SNX11	
			C10orf113	CPS1-IT	
			ZNF438	PRRG3	
			ANGPTL6	MPV17L2	
			ADH6	NPTX2	
			KIR2DL2	TGFBI	
			GRHL1	CTNNA3	
			ARTN	MOSC2	
			LGALS3	UNC13A	
			LILRA1	GSTA3	
			SGCZ	SEMA4A	
			ESRP1	DDAH1	
			KRTAP3-3	LOC284930	
			STXBP2	LOC150622	
			NR1I2	KIAA1024	
			SCN4B	BOLA3	
			NCF1C	PAOX	
			TRIP6	MTFP1	
			MNDA	KATNAL2	
			C10orf27	CAMK2A	
			HSF5	ZNF239	
			ASZ1	C1orf54	
			SPIN2A	LOC339760	
			TLX2	LOC157860	
			FGF8	PRAP1	
			IGSF6	CETP	
			GPX5	CLCN5	
			CLPB	LILRB4	
			IGSF5	LOC339593	
			RIPPLY2	NCRNA00028	
			KL	POU4F1	
			FAM107B	CCR6	
			TYMP	GPR18	
			SOX3	RCBTB2	
			NGB	SMAD6	
			MEGF10	MS4A6A	
			LOC646484	MSLN	
			FAM65B	HLA-DMB	
			SERPINA1	TACSTD2	
			B3GNT3	AK7	
			WNT5B	PGR	
			C13orf18	ZNF517	

			PTPRN2	CYB5R1		
			KBTBD5	KRT73		
			TRIM7	MYH11		
			C5AR1	LIPC		
			TBL1Y	PM20D1		
			NBPF8	C20orf166		
			LOC284798	PACSIN3		
			STX11	LSM14B		
			HLA-DQB1	CKM		
			C2orf50	ZNRF2		
			BAIAP2L1	CAND2		
			ODZ3	OTUD7B		
			DOCK1	PGAM2		
			GOLGA6A	METTL7B		
			RANBP17	LOC284837		
			GGT5	CD180		
			LOC401176	LOC284998		
			UMOD	SLC16A6		
			GALR1	IRF8		
			COL23A1	MX2		
			PPM1M	LOC503519		
			MCOLN1	CCRL2		
			C3orf48	GNG4		
			RIMKLB	S100A16		
			FAM46D	CCDC26		
			FAM3B	TIFAB		
			LPAR5	DLGAP3		
			HSPA6	SPZ1		
			HHLA2	OSTBETA		
			SLC15A3	AP2S1		
			FABP1	CHST2		
			ZSCAN10	HAPLN4		
			RHOU	CCND1		
			LOC283112	KIAA1409		
			LOC643763	TACC2		
			CCR1	NPB		
			ANXA6	ZNF642		
			EYA2	OASL		
			CA5A	ZMAT4		
			SRRM3	GMPPB		
			SUN3	JPH1		
			C9orf72	F8		
			FAAH	ENTPD1		
			SLC27A3	CDH3		

			KCTD4	FAM71F1	
			OR51B4	ADAMTS18	
			B3GAT1	HYLS1	
			C1orf111	PRG2	
			GPER	CALR3	
			TNNI2	CDH17	
			PDGFRA	KMO	
			MANSC1	CACNA2D4	
			C15orf62	KLHL29	
			LY6G6D	HIGD1B	
			CST7	ZNF57	
			NME3	PCDH19	
			LOC100127974	SNCB	
			HSPB1	ACY3	
			CST3	CYP4F2	
			LTK	BACE2	
			EFS	LOC729683	
			LOC100288860	TLR7	
			CDK20	GALR2	
			PAX4	DDO	
			GRB14	ATF7IP2	
			ZNF750	C3orf20	
			OR2F2	CNDP2	
			LOC100505540	GNGT2	
			ARHGAP24	PPT1	
			MAP7D2	TAGLN	
			LHX9	LOC100128025	
			GALM	MYBPC1	
			LDLR	PFKFB4	
			FRMD4A	FAM200B	
			IPCEF1	CXCR2	
			TNFSF12	FARP1	
			HRH1	UPK3A	
			HMGB4	PCDHGC4	
			MGAT5	OTP	
			TNFSF8	CLEC5A	
			GPX3	NAGA	
			SEMA3G	CLEC12A	
			SYT4	GSTTP1	
			TBC1D9	IGFBPL1	
			FLJ39632	EPSTI1	
			C2orf58	SIPA1L2	
			TSPO	KYNU	
			RAB3B	MACC1	

			FPR2	LOC116437	
			BPI	OAS2	
			ITGAL	STYK1	
			ZNF442	CLDN17	
			TNFAIP6	LOC400128	
			CD96	LY86	
			LOC283485	CD93	
			OTUB2	ZNF555	
			PCDH7	NEXN	
			CLSTN2	PI4K2A	
			LILRA2	EPX	
			ZNF815	SPRYD5	
			C6orf124	ME1	
			TNMD	CAPN3	
			FIGF	GTSF1	
			LOC339316	RETN	
			SAA3P	PLG	
			ACE2	CXorf30	
			GPNMB	PTH2R	
			RBP3	SLC26A8	
			HLA-DRA	SV2B	
			CDA	KIF17	
			EPYC	SLC4A3	
			NT5E	MTMR11	
			TDRD7	LOC100128501	
			ANO1	ROGDI	
			UROC1	DNAJA4	
			LOC285286	ST18	
			CLEC10A	IL13RA2	
			C9orf144	MRVI1	
