# Supplementary material for: Activation of Vitamin D Receptor Pathway Enhances Differentiating Capacity in Acute Myeloid Leukemia with Isocitrate Dehydrogenase Mutations
Source: Cancers (Basel). 2021 Oct 19;13(20):5243. doi: 10.3390/cancers13205243 (PMC8533831; doi:10.3390/cancers13205243)
Supplement: Supplementary file 1 [file cancers-13-05243-s001.zip › Table S2.pdf]

Table S2 : List of Pharmacological Substances by cohort (related to Figure 1A)

Pharmacological Substances (Genomatix) enriched in IDH1^{MUT} patient (Verhaak)

ATRA-related Gene signature
VD-related Gene Signature

Pharmacological substance	Pharmacological substance id	P-value	Adjusted p-value	# Genes (observed)	# Genes (expected)	# Genes (total)
TRETINOIN	C0040845	1.58E-03	n/a	94	69.75693547	989
HEPARIN	C0019134	2.38E-04	n/a	50	29.9764384	425
PORCINE HEPARIN	C0770546	2.66E-04	n/a	50	30.11750399	427
POLYSACCHARIDES	C0032594	1.65E-04	n/a	44	24.96860987	354
BROMODEOXYURIDINE	C0006233	5.43E-03	n/a	40	26.37926579	374
INTERFERON TYPE I	C0021743	1.73E-04	n/a	39	21.30090446	302
IONOMYCIN	C0022020	2.20E-04	n/a	38	20.80717489	295
GLYCOSAMINOGLYCANS	C0017973	2.51E-03	n/a	35	21.30090446	302
ATORVASTATIN	C0286651	7.12E-06	n/a	34	15.16455119	215
INTERLEUKIN 12	C0123759	3.08E-07	n/a	32	12.06110816	171
CYTOCHALASINS	C0010739	4.23E-04	n/a	32	17.06893669	242
OLEIC ACID	C0028928	3.53E-03	n/a	32	19.39651896	275
RECOMBINANT INTERFERON	C1522537	7.61E-05	n/a	31	14.88242	211
DITHIOTHREITOL	C0012789	1.72E-03	n/a	31	17.77426465	252
VITAMIN A	C0042839	2.20E-03	n/a	31	18.05639583	256
ALLERGEN	C3274161	4.57E-05	n/a	30	13.82442806	196
INTERFERONS	C0021747	1.80E-04	n/a	30	14.88242	211
INTERFERON	C3652465	1.80E-04	n/a	30	14.88242	211
HUMAN LEUKOCYTE INTERFERON	C0733470	1.95E-04	n/a	30	14.9529528	212
ASPIRIN	C0004057	8.12E-04	n/a	29	15.51721517	220
IMMUNOLOGIC ADJUVANTS	C0001551	1.09E-04	n/a	28	13.1191001	186
HEPATITIS B SURFACE ANTIGENS	C0019168	2.69E-04	n/a	28	13.82442806	196
UROKINASE	C0042071	2.93E-04	n/a	28	13.89496086	197
ALL TRANS RETINOL	C0087161	1.48E-03	n/a	28	15.37614958	218
ADRENAL CORTEX HORMONES	C0001617	1.70E-03	n/a	28	15.51721517	220
SERPINS	C0036764	5.56E-03	n/a	28	16.8573383	239
HYALURONAN	C0813622	3.74E-04	n/a	27	13.40123128	190
CHLOROQUINE	C0008269	1.42E-03	n/a	27	14.60028882	207
PROSTAGLANDINS	C0033554	3.87E-03	n/a	27	15.65828076	222
ACETYL LDL	C0050486	8.28E-08	n/a	26	8.111271566	115
HYDROXYMETHYLGLUTARYL COA REDUCTASE INHI	C0360714	1.25E-03	n/a	26	13.75389526	195
VON WILLEBRAND FACTOR	C0042971	1.58E-07	n/a	25	7.829140382	111
SEPTA	C0723285	2.36E-04	n/a	24	11.00311621	156
RECOMBINANT UROKINASE	C1522670	4.19E-04	n/a	24	11.42631299	162
PREDNISOLONE	C0032950	5.37E-03	n/a	24	13.75389526	195
CHONDROITIN SULFATES	C0008466	6.10E-03	n/a	24	13.89496086	197

DEXTRAN SULFATE	C0079224	1.28E-05	n/a	22	8.04073877	114
INTRAVENOUS IMMUNOGLOBULIN	C0085297	2.96E-05	n/a	22	8.463935548	120
MINOCYCLINE	C0026187	4.66E-04	n/a	22	10.15672266	144
CPG ODN	C0914671	9.83E-04	n/a	22	10.72098503	152
RECOMBINANT ALPHA CHEMOKINE	C1527035	3.57E-03	n/a	22	11.84950977	168
ANGIOGENESIS INHIBITORS	C0596087	8.63E-03	n/a	22	12.76643612	181
RECOMBINANT INTERFERON ALPHA INDUCIBLE PR	C1522712	5.06E-06	n/a	21	7.053279623	100
CARRAGEENAN	C0007289	4.39E-04	n/a	21	9.451394695	134
RITUXIMAB	C0393022	1.15E-03	n/a	21	10.15672266	144
SERINE PROTEINASE INHIBITORS	C0036733	5.66E-03	n/a	21	11.56737858	164
ENDOGLYCOSIDASES	C3537242	9.64E-03	n/a	21	12.13164095	172
BUDESONIDE	C0054201	5.06E-05	n/a	20	7.547009197	107
BETA GLUCANS	C1134651	9.87E-05	n/a	20	7.899673178	112
POLY I C	C0032414	1.23E-03	n/a	20	9.521927491	135
RECOMBINANT MACROPHAGE COLONY STIMULAT	C1524095	2.48E-03	n/a	20	10.08618986	143
FACTOR XIII	C0015528	1.82E-05	n/a	19	6.489017253	92
LIPOPEPTIDES	C2266975	2.50E-05	n/a	19	6.630082846	94
BISPECIFIC ANTIBODY	C0206492	9.32E-05	n/a	19	7.264878012	103
FUCOIDAN	C0060794	1.43E-03	n/a	19	8.957665121	127
IMIQUIMOD	C0165032	1.43E-03	n/a	19	8.957665121	127
HUMAN IMMUNOGLOBULIN A	C2825347	1.72E-03	n/a	19	9.098730714	129
METHYLPREDNISOLONE	C0025815	2.26E-03	n/a	19	9.310329102	132
BLOOD COAGULATION FACTOR	C0005789	4.76E-03	n/a	19	9.945124268	141
PLASMINOGEN ACTIVATOR	C0032144	4.76E-03	n/a	19	9.945124268	141
THERAPEUTIC TUMOR INFILTRATING LYMPHOCYTE	C1515408	5.99E-03	n/a	19	10.15672266	144
PROTEIN KINASE C INHIBITOR	C1514555	8.01E-03	n/a	19	10.43885384	148
MANNANS	C0024727	9.88E-05	n/a	18	6.700615642	95
ULEX EUROPAEUS FLOWER EXTRACT	C3486655	1.71E-04	n/a	18	6.982746827	99
TETANUS TOXOID ADSORBED	C0305062	4.12E-04	n/a	18	7.4764764	106
APROTININ	C0003641	5.83E-04	n/a	18	7.688074789	109
AMPHOTERICIN B	C0002679	7.28E-04	n/a	18	7.829140382	111
TOLONIUM CHLORIDE	C0040380	9.03E-04	n/a	18	7.970205974	113
EPOPROSTENOL	C0033567	1.66E-03	n/a	18	8.393402751	119
METHACHOLINE	C0600370	3.16E-03	n/a	18	8.887132325	126
MONOPHOSPHORYL LIPID A	C0066776	6.28E-06	n/a	17	5.007828532	71
CYTOCHALASIN B	C0010737	4.04E-04	n/a	17	6.841681234	97
VALSARTAN	C0216784	9.22E-04	n/a	17	7.335410808	104
TETANUS VACCINE	C0039620	1.15E-03	n/a	17	7.4764764	106
MERBROMIN	C0025397	1.42E-03	n/a	17	7.617541993	108
IMMUNOTOXINS	C0021084	2.83E-03	n/a	17	8.111271566	115
ANTIPLASMIN	C0003410	6.45E-06	n/a	16	4.514098959	64
RICINUS COMMUNIS WHOLE EXTRACT	C3537211	3.81E-05	n/a	16	5.148894125	73
INDIUM BLEOMYCIN	C0063476	1.17E-03	n/a	16	6.841681234	97
BICUCULLINE	C0005372	2.22E-03	n/a	16	7.264878012	103

TRYPTOPHAN	C0041249	2.22E-03	n/a	16	7.264878012	103
PROTEASE INHIBITORS	C0033607	2.72E-03	n/a	16	7.405943604	105
HEPARIN SEPHAROSE	C0062515	6.20E-03	n/a	16	8.04073877	114
PROSTAGLANDINS I	C0205911	7.32E-03	n/a	16	8.181804363	116
FLUVASTATIN	C0082608	8.61E-03	n/a	16	8.322869955	118
GAMMA GLOBULIN SERUM	C0305052	1.79E-06	n/a	15	3.667705404	52
HIRUDINS	C1451270	2.35E-05	n/a	15	4.443566162	63
HIRUDIN	C0019573	2.35E-05	n/a	15	4.443566162	63
OLIGOADENYLATE	C0133819	3.59E-04	n/a	15	5.572090902	79
GLUCANS	C0017696	9.13E-04	n/a	15	6.065820476	86
CHOLIC ACID	C0055568	1.31E-03	n/a	15	6.277418864	89
CHOLIC ACIDS	C0008404	1.31E-03	n/a	15	6.277418864	89
INFLIXIMAB	C0666743	4.23E-03	n/a	15	7.053279623	100
LOSARTAN	C0126174	5.11E-03	n/a	15	7.194345215	102
CARDIAC GLYCOSIDES	C0007158	7.98E-03	n/a	15	7.547009197	107
OXALATE	C3669135	9.43E-03	n/a	15	7.688074789	109
BCG VACCINE	C0004886	9.43E-03	n/a	15	7.688074789	109
CHONDROITIN 4 SULFATE	C0086008	5.80E-04	n/a	14	5.219426921	74
LOW MOLECULAR WEIGHT HEPARIN	C0019139	1.14E-03	n/a	14	5.572090902	79
ADENOSINE TRIPHOSPHATE	C0001480	1.86E-03	n/a	14	5.854222087	83
RECOMBINANT IMMUNE GLOBULIN	C1527233	2.09E-03	n/a	14	5.924754883	84
FIBRATES	C1449704	4.93E-03	n/a	14	6.489017253	92
MANNITOL	C0024730	9.44E-03	n/a	14	6.982746827	99
MEASLES VACCINE	C0025010	1.97E-06	n/a	13	2.821311849	40
CEFTAZIDIME	C0007559	4.71E-05	n/a	13	3.667705404	52
FACTOR VII	C0015502	1.24E-03	n/a	13	5.007828532	71
ALLOPURINOL	C0002144	2.65E-03	n/a	13	5.43102531	77
CYSTEINYL LEUKOTRIENE	C0675175	3.34E-03	n/a	13	5.572090902	79
OLMESARTAN	C1098320	4.17E-03	n/a	13	5.713156495	81
ARECOLINE	C0003753	4.65E-03	n/a	13	5.783689291	82
PARAFORM	C0070066	5.17E-03	n/a	13	5.854222087	83
PITAVASTATIN	C1101838	8.48E-03	n/a	13	6.206886068	88
PLASMIN INHIBITOR	C0032139	9.17E-05	n/a	12	3.385574219	48
HLA DR ALPHA CHAINS	C0019763	1.14E-04	n/a	12	3.456107015	49
THERAPEUTIC CORYNEBACTERIUM PARVUM	C1522468	5.17E-04	n/a	12	4.020369385	57
DILTIAZEM	C0012373	6.11E-04	n/a	12	4.090902181	58
COMPLEMENT INACTIVATING AGENTS	C1564892	7.19E-04	n/a	12	4.161434978	59
SODIUM SULFIDE	C0074770	1.52E-03	n/a	12	4.514098959	64
MITOMYCINS	C0026254	2.61E-03	n/a	12	4.796230144	68
DIGOXIN	C0012265	2.96E-03	n/a	12	4.86676294	69
POLYMYXIN B	C0032535	3.77E-03	n/a	12	5.007828532	71
CYSTEINE PROTEINASE INHIBITORS	C0010658	3.77E-03	n/a	12	5.007828532	71
ETANERCEPT	C0717758	7.36E-03	n/a	12	5.43102531	77
BACTERICIDAL PERMEABILITY INCREASING PROTEIN	C0052897	2.22E-04	n/a	11	3.17397583	45

ALLERGENIC EXTRACT	C0440016	3.35E-04	n/a	11	3.315041423	47
FACTOR VIIA	C0015505	7.06E-04	n/a	11	3.597172608	51
PIROXICAM	C0031990	9.93E-04	n/a	11	3.7382382	53
AMD 3100	C0662900	1.37E-03	n/a	11	3.879303793	55
OK 432	C0918027	1.37E-03	n/a	11	3.879303793	55
RECOMBINANT INTERLEUKIN 4	C1522540	1.60E-03	n/a	11	3.949836589	56
HYPOLIPIDEMIC AGENTS	C0086440	2.15E-03	n/a	11	4.090902181	58
CERIVASTATIN	C0528023	2.15E-03	n/a	11	4.090902181	58
CURDLAN	C0056608	2.15E-03	n/a	11	4.090902181	58
4 BETA AMINOETHYLBENZOLSULFOFLUORIDE	C0047895	2.84E-03	n/a	11	4.231967774	60
THROMBIN INHIBITORS	C2746042	3.25E-03	n/a	11	4.30250057	61
LIPASE	C0023764	3.25E-03	n/a	11	4.30250057	61
7 AMINOACTINOMYCIN D	C0049832	3.25E-03	n/a	11	4.30250057	61
LEVAMISOLE	C0023556	4.21E-03	n/a	11	4.443566162	63
ITRACONAZOLE	C0064113	4.21E-03	n/a	11	4.443566162	63
BEXAROTENE	C0765273	4.77E-03	n/a	11	4.514098959	64
DESOXYCORTICOSTERONE ACETATE	C0813203	4.77E-03	n/a	11	4.514098959	64
PRISTANE	C0071986	6.80E-03	n/a	11	4.725697347	67
VANILLOID	C1519952	8.50E-03	n/a	11	4.86676294	69
MEDICATED	C3812869	9.46E-03	n/a	11	4.937295736	70
ACETYLMURAMYL ALANYL ISOGLUTAMINE	C0001060	9.46E-03	n/a	11	4.937295736	70
MURAMIDASE	C0026794	3.57E-06	n/a	10	1.763319906	25
CONJUGATE VACCINE	C0206515	1.75E-04	n/a	10	2.609713461	37
PURINERGIC P1 RECEPTOR AGONISTS	C2917322	2.22E-04	n/a	10	2.680246257	38
PHOSPHORAMIDON	C0070915	6.54E-04	n/a	10	3.032910238	43
FIBRIN TISSUE ADHESIVE	C0016004	7.94E-04	n/a	10	3.103443034	44
ABATACEPT	C1619966	1.37E-03	n/a	10	3.315041423	47
THIOPENTAL	C0039925	1.91E-03	n/a	10	3.456107015	49
COMPOUND 48 80	C0009574	1.91E-03	n/a	10	3.456107015	49
RECOMBINANT GRANULOCYTE COLONY STIMULAT	C0376562	2.24E-03	n/a	10	3.526639812	50
HUMAN IMMUNOGLOBULIN G	C2702333	2.61E-03	n/a	10	3.597172608	51
INTERFERON TYPE II	C0021745	3.50E-03	n/a	10	3.7382382	53
CHONDROITIN 6 SULFATE	C0086009	4.04E-03	n/a	10	3.808770996	54
ACYCLOVIR	C0001367	4.63E-03	n/a	10	3.879303793	55
ATENOLOL	C0004147	4.63E-03	n/a	10	3.879303793	55
EGG LYSOZYME	C4040986	6.83E-03	n/a	10	4.090902181	58
CLOTRIMAZOLE	C0009074	7.71E-03	n/a	10	4.161434978	59
CYTOTOXIC AGENT	C0304497	9.75E-03	n/a	10	4.30250057	61
SODIUM HYALURONATE	C0087000	2.07E-05	n/a	9	1.69278711	24
HUMAN THROMBIN	C2369149	2.65E-04	n/a	9	2.257049479	32
ANTI ASTHMATIC AGENTS	C0282556	3.42E-04	n/a	9	2.327582276	33
COAGULATION PROTEIN	C1292266	4.36E-04	n/a	9	2.398115072	34
OLOPATADINE	C0527189	5.51E-04	n/a	9	2.468647868	35
VITAMIN K 1	C0031862	6.89E-04	n/a	9	2.539180664	36

CEFOTAXIME	C0007554	6.89E-04	n/a	9	2.539180664	36
IMMUNOL	C1517844	6.89E-04	n/a	9	2.539180664	36
PHENOXYBENZAMINE	C0031441	1.28E-03	n/a	9	2.750779053	39
FIBRINOGEN HUMAN PLASMA DERIVED	C2587184	1.55E-03	n/a	9	2.821311849	40
MILTEFOSINE	C0068006	1.55E-03	n/a	9	2.821311849	40
ANTIFIBRINOLYTIC AGENTS	C0003304	1.87E-03	n/a	9	2.891844645	41
CASTANOSPERMINE	C0054849	2.23E-03	n/a	9	2.962377442	42
KAOLIN	C0022499	2.23E-03	n/a	9	2.962377442	42
ALTEPLASE	C0032143	3.67E-03	n/a	9	3.17397583	45
ELASTASE INHIBITOR	C0596481	4.28E-03	n/a	9	3.244508627	46
ETOMIDATE	C0015131	4.97E-03	n/a	9	3.315041423	47
SEPRANOLONE	C0376202	6.60E-03	n/a	9	3.456107015	49
GW9662	C1137010	9.79E-03	n/a	9	3.667705404	52
ALUMINUM HYDROXIDE	C0002371	9.79E-03	n/a	9	3.667705404	52
S 27609	C0381150	8.14E-06	n/a	8	1.199057536	17
ACECLOFENAC	C0050403	2.23E-05	n/a	8	1.340123128	19
DEFIBROTIDE	C0057257	3.48E-05	n/a	8	1.410655925	20
ARGATROBAN	C0048470	1.12E-04	n/a	8	1.622254313	23
RECOMBINANT FVIIA	C0534191	5.18E-04	n/a	8	1.974918294	28
3',5' DICHLOROMETHOTREXATE	C0046764	6.71E-04	n/a	8	2.045451091	29
LEPIRUDIN	C0772394	8.59E-04	n/a	8	2.115983887	30
PHOSPHOLIPASE A2 INHIBITORS	C3658209	1.09E-03	n/a	8	2.186516683	31
SERPIN SUPERFAMILY	C0036763	1.36E-03	n/a	8	2.257049479	32
REPAGLINIDE	C0246689	1.36E-03	n/a	8	2.257049479	32
PRANLUKAST	C0526058	1.36E-03	n/a	8	2.257049479	32
FEBUXOSTAT	C0249529	2.52E-03	n/a	8	2.468647868	35
RECOMBINANT INTERFERON BETA 1A	C0982234	2.52E-03	n/a	8	2.468647868	35
ANGIOTENSIN II TYPE 1 RECEPTOR BLOCKERS	C1449680	2.52E-03	n/a	8	2.468647868	35
MINERALOCORTICOID RECEPTOR ANTAGONISTS	C1579268	3.05E-03	n/a	8	2.539180664	36
HLA DR BETA AG	C0019765	3.05E-03	n/a	8	2.539180664	36
SODIUM DODECYL SULFATE	C0037506	3.65E-03	n/a	8	2.609713461	37
LUXOL FAST BLUE MBS	C0065274	3.65E-03	n/a	8	2.609713461	37
PLATELET CONCENTRATE	C2362067	5.14E-03	n/a	8	2.750779053	39
RECOMBINANT INTERFERON GAMMA	C0021740	8.18E-03	n/a	8	2.962377442	42
CUPRAMMONIUM CELLULOSE	C0056591	9.44E-03	n/a	8	3.032910238	43
DIPHENHYDRAMINE	C0012522	9.44E-03	n/a	8	3.032910238	43
HUMAN IMMUNOGLOBULIN M	C2825348	9.44E-03	n/a	8	3.032910238	43
TOBRAMYCIN	C0040341	9.44E-03	n/a	8	3.032910238	43
CEFUROXIME	C0007562	5.52E-05	n/a	7	1.12852474	16
ANTIDIARRHEALS	C0003292	5.52E-05	n/a	7	1.12852474	16
HEMATOPORPHYRIN	C0018959	1.35E-04	n/a	7	1.269590332	18
9 AMINOCAMPTOTHECIN	C0214192	2.01E-04	n/a	7	1.340123128	19
3 HYDROXYKYNURENINE	C0047472	4.10E-04	n/a	7	1.481188721	21
KH 1060	C0167620	4.10E-04	n/a	7	1.481188721	21

CHYMOPAPAIN	C0008736	1.32E-03	n/a	7	1.763319906	25
SYNTHETIC ANTIGENS	C0039115	2.16E-03	n/a	7	1.904385498	27
IMMUNOGLOBULIN G	C0020852	2.16E-03	n/a	7	1.904385498	27
KREBS RINGER SOLUTION	C0064411	2.71E-03	n/a	7	1.974918294	28
IRON DEXTRAN COMPLEX	C0022092	3.35E-03	n/a	7	2.045451091	29
DISUCCINIMIDYL SUBERATE	C0058479	3.35E-03	n/a	7	2.045451091	29
PHOSPHOLIPASE A2 INHIBITOR	C0893636	4.11E-03	n/a	7	2.115983887	30
BIRB 796	C1121746	4.99E-03	n/a	7	2.186516683	31
TOLEROGEN	C0076797	4.99E-03	n/a	7	2.186516683	31
TAPI 2	C0389500	6.01E-03	n/a	7	2.257049479	32
BECLOMETHASONE	C0004905	6.01E-03	n/a	7	2.257049479	32
INTERFERON BETA 1A	C0254119	7.18E-03	n/a	7	2.327582276	33
PROLACTIN RELEASING HORMONE	C0033374	7.18E-03	n/a	7	2.327582276	33
CASPOFUNGIN	C0537894	8.50E-03	n/a	7	2.398115072	34
CETRORELIX	C0209366	8.50E-03	n/a	7	2.398115072	34
LINAGLIPTIN	C2746078	9.99E-03	n/a	7	2.468647868	35
RECOMBINANT INTERLEUKIN 8	C1527432	9.99E-03	n/a	7	2.468647868	35
FREE RETINOL	C3873611	1.99E-05	n/a	6	0.705327962	10
EOSINOPHIL DERIVED NEUROTOXIN	C0059409	1.35E-04	n/a	6	0.916926351	13
THROMBIN	C0040018	2.22E-04	n/a	6	0.987459147	14
RP 73401	C0295452	3.48E-04	n/a	6	1.057991943	15
EZN 2208	C2347621	3.48E-04	n/a	6	1.057991943	15
SR 11237	C0254507	1.08E-03	n/a	6	1.269590332	18
METRIZAMIDE	C0025869	1.48E-03	n/a	6	1.340123128	19
LACTATED RINGER'S SOLUTION	C0073385	1.48E-03	n/a	6	1.340123128	19
LANTHANUM CARBONATE	C0768119	1.48E-03	n/a	6	1.340123128	19
SINGLE CHAIN UROKINASE TYPE PLASMINOGEN AC	C0086987	1.99E-03	n/a	6	1.410655925	20
BOVINE THROMBIN	C1337342	1.99E-03	n/a	6	1.410655925	20
IXAZOMIB	C3273711	2.62E-03	n/a	6	1.481188721	21
ANTITHROMBINS	C0003440	2.62E-03	n/a	6	1.481188721	21
SUPLATAST TOSILATE	C0527067	2.62E-03	n/a	6	1.481188721	21
BRIVANIB ALANINATE	C1948076	3.39E-03	n/a	6	1.551721517	22
NORGESTREL	C0028368	4.32E-03	n/a	6	1.622254313	23
PHENYLALANYL LEUCYL PHENYLALANYL GLUTAMII	C0070651	5.42E-03	n/a	6	1.69278711	24
REACTIVE BLUE 2	C0140012	5.42E-03	n/a	6	1.69278711	24
TOUCHDOWN	C0912256	5.42E-03	n/a	6	1.69278711	24
TENIDAP	C0076092	6.71E-03	n/a	6	1.763319906	25
RIVASTIGMINE	C0649350	6.71E-03	n/a	6	1.763319906	25
MRS 2179	C0674169	6.71E-03	n/a	6	1.763319906	25
HIV VACCINE	C0086413	8.21E-03	n/a	6	1.833852702	26
RESIQUIMOD	C0965594	8.21E-03	n/a	6	1.833852702	26
HYDROETHIDINE	C0063082	9.94E-03	n/a	6	1.904385498	27
PARECOXIB SODIUM	C0915144	9.94E-03	n/a	6	1.904385498	27
HUMAN IMMUNOGLOBULIN PRODUCT	C0358321	9.94E-03	n/a	6	1.904385498	27

BILE ACID SEQUESTRANTS	C2917337	9.94E-03	n/a	6	1.904385498	27
BRIMONIDINE	C0525227	9.94E-03	n/a	6	1.904385498	27
RUBITECAN	C0213800	9.94E-03	n/a	6	1.904385498	27
ORAL ANTICOAGULANTS	C0354604	9.94E-03	n/a	6	1.904385498	27
2' FUCOSYLLACTOSE	C0045214	3.23E-04	n/a	5	0.705327962	10
ANTISTASIN	C0052098	5.57E-04	n/a	5	0.775860759	11
TOLMETIN	C0040377	5.57E-04	n/a	5	0.775860759	11
DG172	C3493533	8.99E-04	n/a	5	0.846393555	12
ACID CYSTEINE PROTEINASE INHIBITORS	C0001097	8.99E-04	n/a	5	0.846393555	12
BC8	C2987608	1.38E-03	n/a	5	0.916926351	13
ZINC SALTS	C0351669	1.38E-03	n/a	5	0.916926351	13
E 7010	C0656781	1.38E-03	n/a	5	0.916926351	13
ZARDAVERINE	C0085049	2.02E-03	n/a	5	0.987459147	14
BIOBRAN	C1527110	2.02E-03	n/a	5	0.987459147	14
PLECONARIL	C0759032	2.02E-03	n/a	5	0.987459147	14
DIRECT FACTOR XA INHIBITORS	C3653500	2.02E-03	n/a	5	0.987459147	14
FACTOR VIII	C0015506	2.02E-03	n/a	5	0.987459147	14
TRASYLOL	C0592198	2.85E-03	n/a	5	1.057991943	15
CUROSURF	C0111318	2.85E-03	n/a	5	1.057991943	15
HUMAN INTRAVENOUS IMMUNOGLOBULINS	C3494178	2.85E-03	n/a	5	1.057991943	15
COD LIVER OIL	C0009213	3.91E-03	n/a	5	1.12852474	16
CANAKINUMAB	C2718773	3.91E-03	n/a	5	1.12852474	16
S 28463	C0389573	3.91E-03	n/a	5	1.12852474	16
INGENOL	C0063537	3.91E-03	n/a	5	1.12852474	16
OKY 046	C0133777	3.91E-03	n/a	5	1.12852474	16
7 NITROBENZ 2 OXA 1,3 DIAZOL 4 YLCHOLESTEROL	C0174000	3.91E-03	n/a	5	1.12852474	16
TRIFLURIDINE	C0040987	3.91E-03	n/a	5	1.12852474	16
COMPSTATIN	C0673968	5.23E-03	n/a	5	1.199057536	17
ANTI TUMOR NECROSIS FACTOR ALPHA DRUG	C1562242	5.23E-03	n/a	5	1.199057536	17
PROTHROMBIN COMPLEX	C3541956	5.23E-03	n/a	5	1.199057536	17
POLY ICLC	C0071359	6.82E-03	n/a	5	1.269590332	18
PITAVASTATIN CALCIUM	C2720023	6.82E-03	n/a	5	1.269590332	18
DIRECT THROMBIN INHIBITORS	C3541936	6.82E-03	n/a	5	1.269590332	18
CORD FACTORS	C0010004	6.82E-03	n/a	5	1.269590332	18
CALCIUM	C0006675	6.82E-03	n/a	5	1.269590332	18
ACYCLIC RETINOID	C0101406	8.73E-03	n/a	5	1.340123128	19
RYE GRASS	C4049835	8.73E-03	n/a	5	1.340123128	19
ZOFENOPRILATE	C0085065	8.73E-03	n/a	5	1.340123128	19
COAGULATION FACTOR VIIIC	C1307126	1.14E-04	n/a	4	0.352663981	5
CI 1044	C0963191	3.29E-04	n/a	4	0.423196777	6
VTX 294	C3713619	3.29E-04	n/a	4	0.423196777	6
TISSUE FACTOR PATHWAY INHIBITOR	C0164707	7.23E-04	n/a	4	0.493729574	7
HIRULOG 1	C0950817	7.23E-04	n/a	4	0.493729574	7
ZHANKUIC ACID A	C0387325	7.23E-04	n/a	4	0.493729574	7

CROFELEMER	C2744960	7.23E-04	n/a	4	0.493729574	7
3,3',4',5 TETRACHLOROSALICYLANILIDE	C0046874	1.36E-03	n/a	4	0.56426237	8
AGNUSIDE	C1122015	1.36E-03	n/a	4	0.56426237	8
ALOSETRON HYDROCHLORIDE	C0291771	1.36E-03	n/a	4	0.56426237	8
DESIRUDIN	C0378366	1.36E-03	n/a	4	0.56426237	8
METHYLENE DIPHOSPHONATE	C0066351	1.36E-03	n/a	4	0.56426237	8
PROXIGERMANIUM	C0081913	2.32E-03	n/a	4	0.634795166	9
TRIAZOLODIAZEPINE	C0245118	2.32E-03	n/a	4	0.634795166	9
TRIAZOLE ANTIFUNGAL	C0360380	2.32E-03	n/a	4	0.634795166	9
HLA G ANTIGEN	C0082920	2.32E-03	n/a	4	0.634795166	9
SUBCUTANEOUS HEPARIN	C0353681	2.32E-03	n/a	4	0.634795166	9
GINSENOSIDE RB2	C0061271	2.32E-03	n/a	4	0.634795166	9
ONO 4007	C0254026	3.66E-03	n/a	4	0.705327962	10
DEAE SEPHADEX A 50	C0057215	3.66E-03	n/a	4	0.705327962	10
BN 50730	C0081833	5.43E-03	n/a	4	0.775860759	11
MONOCLONAL ANTIBODY LL2	C0281743	5.43E-03	n/a	4	0.775860759	11
PYRILAMINE MALEATE	C0282330	5.43E-03	n/a	4	0.775860759	11
BETA AMINOETHYLISOTHIURONIUM DIHYDROBRO	C0001716	5.43E-03	n/a	4	0.775860759	11
BAY X 1005	C0656891	5.43E-03	n/a	4	0.775860759	11
BENRALIZUMAB	C2982078	5.43E-03	n/a	4	0.775860759	11
ASPERGILLUS FUMIGATUS EXTRACT	C1445421	5.43E-03	n/a	4	0.775860759	11
NADROPARIN	C0206232	7.69E-03	n/a	4	0.846393555	12
MCC 555	C0758974	7.69E-03	n/a	4	0.846393555	12
MZM	C0721501	7.69E-03	n/a	4	0.846393555	12
TJ 9	C1138012	7.69E-03	n/a	4	0.846393555	12
SC 41930	C0074112	7.69E-03	n/a	4	0.846393555	12
THROMBIN ALFA	C2240523	7.69E-03	n/a	4	0.846393555	12
FONTANA MASSON STAIN	C0060631	7.69E-03	n/a	4	0.846393555	12
LITHOCHOLATE	C0086564	7.69E-03	n/a	4	0.846393555	12
IRDYE 800CW	C3254109	7.69E-03	n/a	4	0.846393555	12
E5564	C0912379	7.69E-03	n/a	4	0.846393555	12
Z 100	C0149256	7.69E-03	n/a	4	0.846393555	12
5' DEOXY 5 FLUOROCYTIDINE	C0963263	3.50E-04	n/a	3	0.211598389	3
NSC 640488	C0165764	3.50E-04	n/a	3	0.211598389	3
HOMATROPINE	C0062922	3.50E-04	n/a	3	0.211598389	3
EPOXICONAZOLE	C0668753	1.30E-03	n/a	3	0.282131185	4
5 CHLORO 2 METHYL 4 ISOTHIAZOLIN 3 ONE	C0049104	1.30E-03	n/a	3	0.282131185	4
RS 504393	C3657798	1.30E-03	n/a	3	0.282131185	4
DIPHTHERIA TETANUS ACELLULAR PERTUSSIS VACC	C0535644	1.30E-03	n/a	3	0.282131185	4
L 652731	C0124915	1.30E-03	n/a	3	0.282131185	4
INOSINIC ACID	C0021544	3.14E-03	n/a	3	0.352663981	5
ALPIDEM	C0051484	3.14E-03	n/a	3	0.352663981	5
MONOCLONAL ANTIBODY HEFI 1	C0796480	3.14E-03	n/a	3	0.352663981	5
BOVINE SEMINAL RIBONUCLEASE	C0106964	3.14E-03	n/a	3	0.352663981	5

F 4614	C0384949	3.14E-03	n/a	3	0.352663981	5
LEUKADHERIN 1	C3851799	3.14E-03	n/a	3	0.352663981	5
HAES STERIL	C0591584	3.14E-03	n/a	3	0.352663981	5
ROXIFIBAN ACETATE	C3501644	3.14E-03	n/a	3	0.352663981	5
MENINGITIS VACCINE	C0361275	3.14E-03	n/a	3	0.352663981	5
RINTATOLIMOD	C1434913	3.14E-03	n/a	3	0.352663981	5
AMPHOTERICIN B DEOXYCHOLATE	C0051761	3.14E-03	n/a	3	0.352663981	5
EXAMETAZIME	C0175035	3.14E-03	n/a	3	0.352663981	5
FOSCAN	C0701505	3.14E-03	n/a	3	0.352663981	5
MOEXIPRILAT	C0209137	5.94E-03	n/a	3	0.423196777	6
GS 9620	C3658665	5.94E-03	n/a	3	0.423196777	6
PHLOGENZYM	C0769132	5.94E-03	n/a	3	0.423196777	6
BENZYLOXYCARBONYLPHENYLALANYLPHENYLALAI	C0053319	5.94E-03	n/a	3	0.423196777	6
TIAPROFENIC ACID	C0076653	5.94E-03	n/a	3	0.423196777	6
GI 129471	C0257713	5.94E-03	n/a	3	0.423196777	6
CITRATE PHOSPHATE DEXTROSE	C0055816	5.94E-03	n/a	3	0.423196777	6
WORMWOOD EXTRACT	C1095890	5.94E-03	n/a	3	0.423196777	6
BRL 50481	C1566895	5.94E-03	n/a	3	0.423196777	6
ARP 100	C3714018	5.94E-03	n/a	3	0.423196777	6
BL1521	C1528435	5.94E-03	n/a	3	0.423196777	6
3 HYDROXYPYRIDIN 4 ONE	C0908483	5.94E-03	n/a	3	0.423196777	6
HAEMACCEL	C0918273	5.94E-03	n/a	3	0.423196777	6
TECHNETIUM TC 99M PENTETATE	C0080212	5.94E-03	n/a	3	0.423196777	6
ST 1571	C1097576	5.94E-03	n/a	3	0.423196777	6
1 FURAN 2 YL 3 PYRIDIN 2 YL PROPENONE	C1700549	5.94E-03	n/a	3	0.423196777	6
2 AZETIDINONE	C0761491	9.87E-03	n/a	3	0.493729574	7
BEMIPARIN	C0916062	9.87E-03	n/a	3	0.493729574	7
B 428	C0754251	9.87E-03	n/a	3	0.493729574	7
KD IX 73 4	C0388970	9.87E-03	n/a	3	0.493729574	7
ALVEOFACT	C0210638	9.87E-03	n/a	3	0.493729574	7
POLYSORBATES	C0032602	9.87E-03	n/a	3	0.493729574	7
CAELYX	C0701183	9.87E-03	n/a	3	0.493729574	7
MUR NAC L ALA D ISOGLN	C0086659	9.87E-03	n/a	3	0.493729574	7
SYNTHETIC COPOLYMER	C1268899	9.87E-03	n/a	3	0.493729574	7
ACTARIT	C0378042	9.87E-03	n/a	3	0.493729574	7
TORBAFYLLINE	C0076835	9.87E-03	n/a	3	0.493729574	7
GROWTH HORMONE RECEPTOR ANTAGONIST	C1947982	9.87E-03	n/a	3	0.493729574	7
CRISAMICIN A	C0056490	9.87E-03	n/a	3	0.493729574	7
SINGLET	C0723338	9.87E-03	n/a	3	0.493729574	7
HIF 2ALPHA INHIBITOR PT2385	C3896783	9.87E-03	n/a	3	0.493729574	7
PKSI 527	C0084061	9.87E-03	n/a	3	0.493729574	7
S 2238	C0073756	9.87E-03	n/a	3	0.493729574	7
LANOTEPLASE	C0753753	9.87E-03	n/a	3	0.493729574	7
ANTIPROTOZOAL AGENTS	C0003416	9.87E-03	n/a	3	0.493729574	7

ZINQUIN	C0249733	9.87E-03	n/a	3	0.493729574	7
SPIRADOLINE	C0143266	9.87E-03	n/a	3	0.493729574	7
HEXTEND	C1170317	9.87E-03	n/a	3	0.493729574	7
AN3485	C3661155	9.87E-03	n/a	3	0.493729574	7
PEG PLGA	C3849743	9.87E-03	n/a	3	0.493729574	7
AN6414	C3661233	4.97E-03	n/a	2	0.141065592	2
JZP 430	C4079896	4.97E-03	n/a	2	0.141065592	2
DIPHTHERIA ANTITOXIN	C0012547	4.97E-03	n/a	2	0.141065592	2
SUCCINYLALBUMIN	C0075459	4.97E-03	n/a	2	0.141065592	2
OCTOCOG ALFA	C1691209	4.97E-03	n/a	2	0.141065592	2
CORBRIN	C0390439	4.97E-03	n/a	2	0.141065592	2
REACTIVE RED 2	C0387847	4.97E-03	n/a	2	0.141065592	2
MS 247	C0908719	4.97E-03	n/a	2	0.141065592	2
BRIMONIDINE TARTRATE TIMOLOL MALEATE DRUG	C4042830	4.97E-03	n/a	2	0.141065592	2
PENTETATE	C1702916	4.97E-03	n/a	2	0.141065592	2
AM 5206	C3177710	4.97E-03	n/a	2	0.141065592	2
ARACYTIN	C1511628	4.97E-03	n/a	2	0.141065592	2
OCTIMIBATE	C0069352	4.97E-03	n/a	2	0.141065592	2
SY 640	C0385278	4.97E-03	n/a	2	0.141065592	2
ATROPA BELLADONNA	C0301370	4.97E-03	n/a	2	0.141065592	2
CHO DEUNG SAN	C1566301	4.97E-03	n/a	2	0.141065592	2
LY 223982	C0083281	4.97E-03	n/a	2	0.141065592	2
RU 27987	C0073665	4.97E-03	n/a	2	0.141065592	2
HUMAN PLASMA PREPARATION	C0304926	4.97E-03	n/a	2	0.141065592	2
EUROPEAN OLIVE POLLEN EXTRACT	C2701713	4.97E-03	n/a	2	0.141065592	2
PENTADECYLAMINE	C3501270	4.97E-03	n/a	2	0.141065592	2
CGS 23885	C0527949	4.97E-03	n/a	2	0.141065592	2
TETRASTARCH	C1816356	4.97E-03	n/a	2	0.141065592	2
BALUGRASTIM	C3871462	4.97E-03	n/a	2	0.141065592	2
LERISETRON	C0758799	4.97E-03	n/a	2	0.141065592	2
MHY407	C3661098	4.97E-03	n/a	2	0.141065592	2
ZOLPIDEM TARTRATE	C0724725	4.97E-03	n/a	2	0.141065592	2
CARBOXYCINNAMIC ACID BISHYDROXAMIDE	C0672711	4.97E-03	n/a	2	0.141065592	2
OPHTHALMIC PREPARATION	C0304650	4.97E-03	n/a	2	0.141065592	2
FIRATEGRAST	C2700183	4.97E-03	n/a	2	0.141065592	2
CT 1418	C0764328	4.97E-03	n/a	2	0.141065592	2
MDL 27695	C0065806	4.97E-03	n/a	2	0.141065592	2
RECOMBINANT HUMAN ALBUMIN HUMAN GRAN	C2825833	4.97E-03	n/a	2	0.141065592	2
1 CYCLOHEXYL 3 DODECYLUREA	C1121212	4.97E-03	n/a	2	0.141065592	2
GALMIC	C1455539	4.97E-03	n/a	2	0.141065592	2
BMS 817399	C3886791	4.97E-03	n/a	2	0.141065592	2
BMS 457	C3713233	4.97E-03	n/a	2	0.141065592	2
CDMI	C0045683	4.97E-03	n/a	2	0.141065592	2
PROVERA	C0699702	4.97E-03	n/a	2	0.141065592	2

DEXTRAN T 70	C0011802	4.97E-03	n/a	2	0.141065592	2
HAEMOPHILUS INFLUENZAE TYPE B POLYSACCHAR	C0062085	4.97E-03	n/a	2	0.141065592	2
LEONURUS CARDIACA EXTRACT	C3486751	4.97E-03	n/a	2	0.141065592	2
1,2 DIAMINO 4,5 METHYLENEDIOXYBENZENE	C0209062	4.97E-03	n/a	2	0.141065592	2
ICI 128436	C0123076	4.97E-03	n/a	2	0.141065592	2
M NITROBENZOYLALANINE	C0252609	4.97E-03	n/a	2	0.141065592	2
LYSOCELLIN	C0065382	4.97E-03	n/a	2	0.141065592	2
SSR180575	C1136821	4.97E-03	n/a	2	0.141065592	2

Pharmacological Substances (Genomatix) enriched in IDH1^{MUT} patient (TCGA)

ATRA-related Gene signature
VD-related Gene Signature

Pharmacological substance	macological substan	P-value	Adjusted p-value	# Genes (observed)	# Genes (expected)	# Genes (total)
TRETINOIN	C0040845	1.05E-04	n/a	15	5.03633047	989
RETINOIDS	C0035339	2.04E-04	n/a	13	4.155354564	816
TRICHOSTATIN A	C0077063	3.27E-03	n/a	8	2.490157331	489
CYCLOHEXIMIDE	C0010572	3.02E-03	n/a	7	1.924906894	378
CYCLIN DEPENDENT KINASE INHIBITOR AGENTS	C1511576	2.47E-03	n/a	6	1.374933495	270
FUCOIDAN	C0060794	4.63E-04	n/a	5	0.646727978	127
HYDROCORTISONE	C0020268	1.59E-03	n/a	5	0.850421829	167
VP 16	C0733688	8.40E-03	n/a	5	1.257809531	247
BACTERICIDAL PERMEABILITY INCREASING PROTEIN	C0052897	7.82E-05	n/a	4	0.229155583	45
PREGNANT MARE SERUM GONADOTROPINS	C1136247	1.18E-03	n/a	4	0.463403511	91
TYRPHOSTIN AG 1478	C0528985	3.64E-03	n/a	4	0.631450939	124
BAFILOMYCIN A1	C0052919	4.20E-03	n/a	4	0.65691267	129
ANISOMYCIN	C0003082	6.19E-03	n/a	4	0.733297864	144
8 BROMO CYCLIC ADENOSINE MONOPHOSPHATE	C0000661	7.64E-03	n/a	4	0.779128981	153
CERULETIDE	C0006639	9.70E-03	n/a	4	0.83514479	164
RECOMBINANT FIBROBLAST GROWTH FACTOR 2	C1522505	7.99E-04	n/a	3	0.183324466	36
HERCEPTIN	C0338204	1.44E-03	n/a	3	0.224063236	44
TRAUMEEL S	C0968917	1.74E-03	n/a	3	0.239340275	47
IMO	C1517800	2.60E-03	n/a	3	0.274986699	54
AMPICILLIN	C0002680	4.79E-03	n/a	3	0.341187201	67
AMPICILLINS	C2095775	4.79E-03	n/a	3	0.341187201	67
RICINUS COMMUNIS WHOLE EXTRACT	C3537211	6.09E-03	n/a	3	0.371741278	73
DYNE VETERINARY DRUG	C0308914	8.39E-03	n/a	3	0.417572395	82
MINERALOCORTICOIDS	C0026160	9.26E-03	n/a	3	0.432849434	85
NITRIC OXIDE SYNTHASE INHIBITOR	C1373060	9.56E-03	n/a	3	0.43794178	86
LEX 032	C0381874	3.78E-04	n/a	2	0.030554078	6
DIFID	C3490744	7.01E-04	n/a	2	0.04073877	8
ARJUNOLIC ACID	C0638629	8.99E-04	n/a	2	0.045831117	9
FACTOR XIIA INHIBITOR	C0208681	1.12E-03	n/a	2	0.050923463	10
MILATUZUMAB	C2716103	1.36E-03	n/a	2	0.056015809	11

TACALCITOL	C0953467	2.23E-03	n/a	2	0.071292848	14
BACTERICIDE	C0004635	2.57E-03	n/a	2	0.076385194	15
HUMAN NEUTROPHIL PEPTIDE 1	C0122620	3.31E-03	n/a	2	0.086569887	17
TEZOSENTAN	C0907956	3.31E-03	n/a	2	0.086569887	17
BB 3103	C0666068	3.71E-03	n/a	2	0.091662233	18
DEFIBROTIDE	C0057257	4.57E-03	n/a	2	0.101846926	20
TENOXICAM	C0076096	5.04E-03	n/a	2	0.106939272	21
SIVELESTAT	C0130137	5.53E-03	n/a	2	0.112031618	22
HUMAN NEUTROPHIL PEPTIDE 2	C0122159	5.53E-03	n/a	2	0.112031618	22
AZILSARTAN	C1958569	6.56E-03	n/a	2	0.122216311	24
ADRENERGIC AGENTS	C0001637	7.11E-03	n/a	2	0.127308657	25
CHYMOPAPAIN	C0008736	7.11E-03	n/a	2	0.127308657	25
BET BROMODOMAINS 2/3/4 INHIBITOR OTX015	C3641185	8.26E-03	n/a	2	0.13749335	27
PIRARUBICIN	C0071126	8.87E-03	n/a	2	0.142585696	28
STRONTIUM RANELATE	C0936139	8.87E-03	n/a	2	0.142585696	28
WEB 2086	C0078460	9.49E-03	n/a	2	0.147678042	29
MESNA	C0000294	9.49E-03	n/a	2	0.147678042	29

Pharmacological Substances (Genomatix) enriched in H^{R132H}

ATRA-related Gene signature
VD-related Gene Signature

Pharmacological substance	macological substan	P-value	Adjusted p-value	# Genes (observed)	# Genes (expected)	# Genes (total)
TRETINOIN	C0040845	3.21E-03	n/a	29	16.9882192	989
DEXAMETHASONE	C0011777	1.75E-05	n/a	22	8.00456031	466
DOXORUBICIN	C0013089	5.91E-03	n/a	21	11.54305693	672
IONOMYCIN	C0022020	3.38E-06	n/a	18	5.067264574	295
HEPARIN	C0019134	1.08E-03	n/a	17	7.30029642	425
PORCINE HEPARIN	C0770546	1.14E-03	n/a	17	7.334650756	427
PROGESTERONE	C0033308	4.25E-03	n/a	16	7.626662613	444
INTERLEUKIN 12	C0123759	1.46E-06	n/a	14	2.937295736	171
INTERFERON TYPE I	C0021743	7.36E-04	n/a	14	5.18750475	302
GLYCOSAMINOGLYCANS	C0017973	7.36E-04	n/a	14	5.18750475	302
ENDOPEPTIDASES	C0030946	1.28E-03	n/a	14	5.496693775	320
INDOMETHACIN	C0021246	2.66E-06	n/a	13	2.662461047	155
RECOMBINANT ALPHA CHEMOKINE	C1527035	6.50E-06	n/a	13	2.885764232	168
ALLERGEN	C3274161	3.39E-05	n/a	13	3.366724937	196
DINOPROSTONE	C0012472	8.78E-05	n/a	13	3.69309113	215
SERPINS	C0036764	2.51E-04	n/a	13	4.105343163	239
SODIUM BUTYRATE	C0142812	5.04E-04	n/a	13	4.414532188	257
BLEOMYCIN	C0005740	5.28E-03	n/a	13	5.754351296	335
FUCOIDAN	C0060794	1.86E-06	n/a	12	2.181500342	127
SB 203580	C0297666	6.83E-05	n/a	12	3.109067417	181
CHYMOTRYPSIN	C0008742	1.20E-04	n/a	12	3.298016265	192

CYTOCHALASINS	C0010739	9.81E-04	n/a	12	4.156874667	242
TYROSINE	C0041485	5.89E-03	n/a	12	5.170327582	301
BUDESONIDE	C0054201	2.20E-06	n/a	11	1.837956981	107
SERINE PROTEINASE INHIBITORS	C0036733	1.24E-04	n/a	11	2.81705556	164
VITAMIN D3	C3265062	1.98E-04	n/a	11	2.971650072	173
PREDNISOLONE	C0032950	5.52E-04	n/a	11	3.349547769	195
INTERFERON	C3652465	1.06E-03	n/a	11	3.624382458	211
INTERFERONS	C0021747	1.06E-03	n/a	11	3.624382458	211
RECOMBINANT INTERFERON	C1522537	1.06E-03	n/a	11	3.624382458	211
HUMAN LEUKOCYTE INTERFERON	C0733470	1.10E-03	n/a	11	3.641559626	212
ASPIRIN	C0004057	1.48E-03	n/a	11	3.77897697	220
VP 16	C0733688	3.65E-03	n/a	11	4.242760508	247
BORTEZOMIB	C1176309	6.49E-03	n/a	11	4.586303869	267
CYTOCHALASIN B	C0010737	6.25E-06	n/a	10	1.666185301	97
RECOMBINANT INTERFERON ALPHA INDUCIBLE PR	C1522712	8.22E-06	n/a	10	1.717716805	100
BETA GLUCANS	C1134651	2.25E-05	n/a	10	1.923842821	112
IBUPROFEN	C0020740	3.54E-05	n/a	10	2.02690583	118
SODIUM ARSENITE	C0074719	4.71E-05	n/a	10	2.095614502	122
RECOMBINANT MACROPHAGE COLONY STIMULAT	C1524095	1.79E-04	n/a	10	2.456335031	143
HEPATITIS B SURFACE ANTIGENS	C0019168	2.08E-03	n/a	10	3.366724937	196
UROKINASE	C0042071	2.16E-03	n/a	10	3.383902105	197
CHONDROITIN SULFATES	C0008466	2.16E-03	n/a	10	3.383902105	197
CYCLOPHOSPHAMIDE	C0010583	4.75E-03	n/a	10	3.77897697	220
PROSTAGLANDINS	C0033554	5.06E-03	n/a	10	3.813331307	222
ARACHIDONATE 5 LIPOXYGENASE INHIBITORS	C1289971	1.26E-07	n/a	9	0.841681234	49
ALPHA 1 ANTITRYPSIN	C0002191	1.35E-06	n/a	9	1.099338755	64
GLUCANS	C0017696	1.62E-05	n/a	9	1.477236452	86
PROLINEDITHIOCARBAMATE	C0084183	2.80E-05	n/a	9	1.58029946	92
TANSHINONE II A	C0897640	5.03E-05	n/a	9	1.700539637	99
MERBROMIN	C0025397	9.95E-05	n/a	9	1.855134149	108
APROTININ	C0003641	1.07E-04	n/a	9	1.872311317	109
TOLONIUM CHLORIDE	C0040380	1.41E-04	n/a	9	1.941019989	113
BAPETA	C0043775	1.96E-04	n/a	9	2.02690583	118
ALPROSTADIL	C0002335	2.52E-04	n/a	9	2.095614502	122
BAICALEIN	C0052927	3.02E-04	n/a	9	2.147146006	125
CELECOXIB	C0538927	3.02E-04	n/a	9	2.147146006	125
ARA C	C0733521	3.21E-04	n/a	9	2.164323174	126
PROTAMINES	C0033603	3.61E-04	n/a	9	2.19867751	128
CARRAGEENAN	C0007289	5.05E-04	n/a	9	2.301740518	134
MINOCYCLINE	C0026187	8.49E-04	n/a	9	2.473512199	144
RITUXIMAB	C0393022	8.49E-04	n/a	9	2.473512199	144
PEPTIDOMIMETICS	C2936235	1.03E-03	n/a	9	2.542220871	148
DEOXYGLUCOSE	C0011501	2.38E-03	n/a	9	2.868587064	167
CHOLECALCIFEROL	C0008318	4.37E-03	n/a	9	3.143421753	183

IMMUNOLOGIC ADJUVANTS	C0001551	4.86E-03	n/a	9	3.194953257	186
BACTERICIDAL PERMEABILITY INCREASING PROTEIN	C0052897	8.35E-07	n/a	8	0.772972562	45
SODIUM SALICYLATE	C0037549	2.26E-06	n/a	8	0.87603557	51
REBAMIPIDE	C0069562	6.13E-06	n/a	8	0.996275747	58
INFLAMMATION MEDIATORS	C0243042	1.02E-05	n/a	8	1.064984419	62
ADENOSINE ETHYLCARBOXAMIDE 05 N	C0050789	1.15E-05	n/a	8	1.082161587	63
RECOMBINANT CYTOKINES	C1524094	1.83E-05	n/a	8	1.150870259	67
POLYMYXIN B	C0032535	2.81E-05	n/a	8	1.219578931	71
RICINUS COMMUNIS WHOLE EXTRACT	C3537211	3.45E-05	n/a	8	1.253933267	73
CYSTEINYL LEUKOTRIENE	C0675175	6.12E-05	n/a	8	1.356996276	79
MANNANS	C0024727	2.26E-04	n/a	8	1.631830965	95
BISPECIFIC ANTIBODY	C0206492	3.92E-04	n/a	8	1.769248309	103
PROTEASE INHIBITORS	C0033607	4.47E-04	n/a	8	1.803602645	105
TRIPTOLIDE	C0077274	1.14E-03	n/a	8	2.078437334	121
HERBIMYCIN	C0062565	1.72E-03	n/a	8	2.215854678	129
METHYLPREDNISOLONE	C0025815	1.99E-03	n/a	8	2.267386182	132
GLYCOSIDE HYDROLASES	C0017976	2.29E-03	n/a	8	2.318917686	135
FENRETINIDE	C0060197	2.51E-03	n/a	8	2.353272022	137
PROTEIN KINASE INHIBITORS	C1449702	5.32E-03	n/a	8	2.662461047	155
SURAMIN	C0038880	5.74E-03	n/a	8	2.696815383	157
RECOMBINANT UROKINASE	C1522670	6.90E-03	n/a	8	2.782701224	162
CERULETIDE	C0006639	7.41E-03	n/a	8	2.81705556	164
4 HYDROXY 2 NONENAL	C0048297	7.95E-03	n/a	8	2.851409896	166
HYDROCORTISONE	C0020268	8.23E-03	n/a	8	2.868587064	167
LUTEOLIN	C0065264	9.74E-03	n/a	8	2.954472904	172
SIVELESTAT	C0130137	5.51E-08	n/a	7	0.377897697	22
LIPOXYGENASE INHIBITORS	C0085385	1.80E-06	n/a	7	0.601200882	35
PERITONEAL DIALYSIS SOLUTION	C0353714	1.80E-06	n/a	7	0.601200882	35
ANTI INFLAMMATORY AGENTS	C0003209	7.66E-06	n/a	7	0.738618226	43
ELASTASE INHIBITOR	C0596481	1.22E-05	n/a	7	0.79014973	46
RECOMBINANT GRANULOCYTE COLONY STIMULAT	C0376562	2.14E-05	n/a	7	0.858858402	50
PEONIFLORIN	C0070320	2.45E-05	n/a	7	0.87603557	51
OK 432	C0918027	4.05E-05	n/a	7	0.944744243	55
4 BETA AMINOETHYLBENZOLSULFOFLUORIDE	C0047895	7.16E-05	n/a	7	1.030630083	60
7 AMINOACTINOMYCIN D	C0049832	7.97E-05	n/a	7	1.047807251	61
BETAMETHASONE	C0005308	7.97E-05	n/a	7	1.047807251	61
TOSYLPHENYLALANYL CHLOROMETHYL KETONE	C0040498	1.60E-04	n/a	7	1.168047427	68
RECOMBINANT FLT3 LIGAND	C1522457	1.76E-04	n/a	7	1.185224595	69
ACETYLMURAMYL ALANYL ISOGLUTAMINE	C0001060	1.92E-04	n/a	7	1.202401763	70
CALCIPOTRIENE	C0065767	3.49E-04	n/a	7	1.32264194	77
LOW MOLECULAR WEIGHT HEPARIN	C0019139	4.08E-04	n/a	7	1.356996276	79
DIMETHYL FUMARATE	C0058218	4.41E-04	n/a	7	1.374173444	80
ADALIMUMAB	C1122087	6.84E-04	n/a	7	1.477236452	86
FACTOR XIII	C0015528	1.02E-03	n/a	7	1.58029946	92

LIPOPEPTIDES	C2266975	1.16E-03	n/a	7	1.614653796	94
TELMISARTAN	C0248719	1.31E-03	n/a	7	1.649008133	96
PEPSIN A	C0030909	1.66E-03	n/a	7	1.717716805	100
MEPRIN	C0733413	2.72E-03	n/a	7	1.872311317	109
EPOPROSTENOL	C0033567	4.43E-03	n/a	7	2.044082998	119
MITOGEN ACTIVATED PROTEIN KINASE INHIBITOR	C1513344	7.74E-03	n/a	7	2.267386182	132
SULFORAFAN	C0163159	8.38E-03	n/a	7	2.301740518	134
MONENSIN	C0026408	9.77E-03	n/a	7	2.370449191	138
BACTERICIDE	C0004635	1.06E-07	n/a	6	0.257657521	15
FILGRASTIM	C0210630	3.76E-07	n/a	6	0.309189025	18
NEDOCROMIL SODIUM	C0886658	1.42E-06	n/a	6	0.377897697	22
PYRROLIDINEDITHIOCARBAMATE	C1313719	4.15E-06	n/a	6	0.446606369	26
FOSFOMYCIN	C0016610	5.26E-06	n/a	6	0.463783537	27
PROCATEROL	C0072032	6.60E-06	n/a	6	0.480960705	28
ALPHA,BETA METHYLENEADENOSINE 5' TRIPHOSPH	C0051285	2.16E-05	n/a	6	0.584023714	34
ZINC OXIDE	C0043491	2.57E-05	n/a	6	0.601200882	35
VISCUM ALBUM PREPARATION	C1166209	6.54E-05	n/a	6	0.70426389	41
RECOMBINANT INTERFERON GAMMA	C0021740	7.52E-05	n/a	6	0.721441058	42
SALMETEROL	C0073992	1.12E-04	n/a	6	0.772972562	45
SU 5416	C0762592	1.12E-04	n/a	6	0.772972562	45
PHENYLMETHYLSULFONYL FLUORIDE	C0031493	2.03E-04	n/a	6	0.858858402	50
BUTEIN	C0054245	3.45E-04	n/a	6	0.944744243	55
RECOMBINANT INTERLEUKIN 4	C1522540	3.81E-04	n/a	6	0.961921411	56
TOFACITINIB	C2930696	4.20E-04	n/a	6	0.979098579	57
MYXOVIRUS RESISTANCE PROTEINS	C0072378	5.07E-04	n/a	6	1.013452915	59
OSTHOL	C0069679	6.07E-04	n/a	6	1.047807251	61
CYCLOOXYGENASE 2 INHIBITORS	C1257954	6.07E-04	n/a	6	1.047807251	61
THROMBIN INHIBITORS	C2746042	6.07E-04	n/a	6	1.047807251	61
TETRAMETHYLPYRAZINE	C0076305	6.62E-04	n/a	6	1.064984419	62
HIRUDIN	C0019573	7.22E-04	n/a	6	1.082161587	63
LEVAMISOLE	C0023556	7.22E-04	n/a	6	1.082161587	63
NS398	C1567346	7.22E-04	n/a	6	1.082161587	63
HIRUDINS	C1451270	7.22E-04	n/a	6	1.082161587	63
HYALURONATE	C0178695	8.53E-04	n/a	6	1.116515923	65
CHONDROITIN 4 SULFATE	C0086008	1.68E-03	n/a	6	1.271110436	74
BRYOSTATIN 1	C0054173	1.93E-03	n/a	6	1.305464772	76
BISINDOLYLMALEIMIDE I	C0091914	2.06E-03	n/a	6	1.32264194	77
ALLOPURINOL	C0002144	2.06E-03	n/a	6	1.32264194	77
MATRINE	C0065751	2.35E-03	n/a	6	1.356996276	79
NARINGIN	C0068451	2.35E-03	n/a	6	1.356996276	79
URSOLIC ACID	C0077938	2.51E-03	n/a	6	1.374173444	80
CARNOSINE	C0007267	2.67E-03	n/a	6	1.391350612	81
OXOPHENYLARSINE	C0069795	2.84E-03	n/a	6	1.40852778	82
GROWTH INHIBITORS	C0018282	3.02E-03	n/a	6	1.425704948	83

CORDYCEPIN	C0056331	3.02E-03	n/a	6	1.425704948	83
THIAZOLIDINEDIONES	C1257987	3.21E-03	n/a	6	1.442882116	84
COBALTOUS CHLORIDE	C0056036	3.40E-03	n/a	6	1.460059284	85
2,4 THIAZOLIDINEDIONE	C0289779	3.60E-03	n/a	6	1.477236452	86
ADENINE NUCLEOTIDES	C0001413	3.60E-03	n/a	6	1.477236452	86
CHRYSIN	C0055661	4.27E-03	n/a	6	1.528767956	89
RIBOFLAVIN	C0035527	4.51E-03	n/a	6	1.545945124	90
SALICYLATE	C0036075	5.29E-03	n/a	6	1.597476628	93
SALICYLATES	C0036077	5.29E-03	n/a	6	1.597476628	93
NITRITE ION	C3848529	5.58E-03	n/a	6	1.614653796	94
CATECHIN	C0007404	5.87E-03	n/a	6	1.631830965	95
OVARIAN HORMONE	C0729511	6.17E-03	n/a	6	1.649008133	96
MATRIX METALLOPROTEINASE INHIBITORS	C1513016	6.49E-03	n/a	6	1.666185301	97
INFLIXIMAB	C0666743	7.50E-03	n/a	6	1.717716805	100
PENICILLIN	C0220892	7.86E-03	n/a	6	1.734893973	101
PENICILLINS	C0030842	7.86E-03	n/a	6	1.734893973	101
BENZYLOXYCARBONYLVALYL ALANYL ASPARTYL FLU	C0383779	8.24E-03	n/a	6	1.752071141	102
PARTHENOLIDE	C0070126	8.24E-03	n/a	6	1.752071141	102
LYCOPENE	C0065331	8.24E-03	n/a	6	1.752071141	102
CILOSTAZOL	C0055729	9.44E-03	n/a	6	1.803602645	105
HA 1A	C0120659	7.68E-08	n/a	5	0.137417344	8
MAPRACORAT	C3252363	7.48E-06	n/a	5	0.292011857	17
DEFIBROTIDE	C0057257	1.80E-05	n/a	5	0.343543361	20
ONO 1301	C0538030	5.74E-05	n/a	5	0.429429201	25
CORM 2	C1120350	8.48E-05	n/a	5	0.463783537	27
WEB 2086	C0078460	1.21E-04	n/a	5	0.498137873	29
NIACINAMIDE	C0028027	1.44E-04	n/a	5	0.515315041	30
INDO 1	C0063477	1.97E-04	n/a	5	0.549669378	32
NINTEDANIB	C2930789	2.29E-04	n/a	5	0.566846546	33
LAMINARAN	C0064631	2.65E-04	n/a	5	0.584023714	34
MK 886	C0128640	3.05E-04	n/a	5	0.601200882	35
KETOROLAC	C0073631	3.49E-04	n/a	5	0.61837805	36
CEFOTAXIME	C0007554	3.49E-04	n/a	5	0.61837805	36
SODIUM DODECYL SULFATE	C0037506	3.98E-04	n/a	5	0.635555218	37
PURINERGIC P1 RECEPTOR AGONISTS	C2917322	4.52E-04	n/a	5	0.652732386	38
ALLOGENEIC LYMPHOCYTES	C0879438	5.12E-04	n/a	5	0.669909554	39
INTRAVESICAL BCG	C1289970	5.77E-04	n/a	5	0.687086722	40
ABATACEPT	C1619966	1.22E-03	n/a	5	0.807326898	47
RADIOIMMUNOCONJUGATES	C0243019	1.34E-03	n/a	5	0.824504066	48
ITF 2357	C1571394	1.62E-03	n/a	5	0.858858402	50
GLIOTOXIN	C0017641	1.62E-03	n/a	5	0.858858402	50
ALLICIN	C0051200	1.62E-03	n/a	5	0.858858402	50
FLUTICASONE	C0082607	1.93E-03	n/a	5	0.893212738	52
PIROXICAM	C0031990	2.10E-03	n/a	5	0.910389907	53

NS 398	C0215737	2.28E-03	n/a	5	0.927567075	54
CAP18 LIPOPOLYSACCHARIDE BINDING PROTEIN	C0108282	2.28E-03	n/a	5	0.927567075	54
TRANS RESVERATROL	C1101322	2.28E-03	n/a	5	0.927567075	54
DIALYSIS SOLUTIONS	C0011947	2.48E-03	n/a	5	0.944744243	55
EFAVIRENZ	C0674428	2.90E-03	n/a	5	0.979098579	57
CAFFEIC ACID	C0054433	2.90E-03	n/a	5	0.979098579	57
EGG LYSOZYME	C4040986	3.13E-03	n/a	5	0.996275747	58
PAMIDRONATE	C0043603	3.13E-03	n/a	5	0.996275747	58
OMPTIN	C0285969	3.13E-03	n/a	5	0.996275747	58
NIGERICIN	C0028076	3.63E-03	n/a	5	1.030630083	60
TRANILAST	C0146337	3.90E-03	n/a	5	1.047807251	61
EDELFOSINE	C0044549	3.90E-03	n/a	5	1.047807251	61
ERYTHROPOIETIN	C0014822	4.48E-03	n/a	5	1.082161587	63
CIGLITAZONE	C0055721	4.79E-03	n/a	5	1.099338755	64
RHEIN	C0073177	5.12E-03	n/a	5	1.116515923	65
SULINDAC SULFIDE	C0075615	5.46E-03	n/a	5	1.133693091	66
SULFASALAZINE	C0036078	6.20E-03	n/a	5	1.168047427	68
ILOPROST	C0079594	6.59E-03	n/a	5	1.185224595	69
IMMUNE SERA	C0020960	6.59E-03	n/a	5	1.185224595	69
NELFINAVIR	C0525005	6.59E-03	n/a	5	1.185224595	69
PROTEIN SYNTHESIS INHIBITORS	C0033671	7.00E-03	n/a	5	1.202401763	70
PYRROLIDINE DITHIOCARBAMATE	C0163305	7.43E-03	n/a	5	1.219578931	71
MONOPHOSPHORYL LIPID A	C0066776	7.43E-03	n/a	5	1.219578931	71
CLODRONATE	C0162357	7.88E-03	n/a	5	1.236756099	72
CLARITHROMYCIN	C0055856	9.32E-03	n/a	5	1.288287604	75
SILYMARIN	C0037135	9.84E-03	n/a	5	1.305464772	76
ICI 200355	C0123089	2.85E-06	n/a	4	0.120240176	7
ONO 5046	C0133951	1.64E-05	n/a	4	0.17177168	10
ISOACTEOSIDE	C0641716	5.37E-05	n/a	4	0.223303185	13
CYL 26Z	C1613957	5.37E-05	n/a	4	0.223303185	13
CP 105696	C0289246	5.37E-05	n/a	4	0.223303185	13
RWJ 67657	C0910100	7.41E-05	n/a	4	0.240480353	14
THENOYLTRIFLUOROACETONE	C0039757	7.41E-05	n/a	4	0.240480353	14
TEPOXALIN	C0145162	9.97E-05	n/a	4	0.257657521	15
RECOMBINANT GRANULOCYTE MACROPHAGE COI	C0376563	1.31E-04	n/a	4	0.274834689	16
URANYL ACETATE	C0077854	2.15E-04	n/a	4	0.309189025	18
SR 11237	C0254507	2.15E-04	n/a	4	0.309189025	18
5 CHLOROMETHYLFLUORESCEIN DIACETATE	C0110185	2.15E-04	n/a	4	0.309189025	18
BORRELIDIN	C0053956	2.68E-04	n/a	4	0.326366193	19
OLPRINONE	C0637063	2.68E-04	n/a	4	0.326366193	19
POLAPREZINC	C0286382	3.31E-04	n/a	4	0.343543361	20
EPINASTINE	C0078442	3.31E-04	n/a	4	0.343543361	20
CILOMILAST	C0968887	3.31E-04	n/a	4	0.343543361	20
ADSORBENTS	C0350056	4.03E-04	n/a	4	0.360720529	21

KH 1060	C0167620	4.03E-04	n/a	4	0.360720529	21
SHETA2	C1260101	4.86E-04	n/a	4	0.377897697	22
SC 236	C0767382	5.81E-04	n/a	4	0.395074865	23
RECOMBINANT HUMAN GRANULOCYTE COLONY S	C1271481	5.81E-04	n/a	4	0.395074865	23
METHIONYLMETHYLSULFONIUM CHLORIDE	C0658922	5.81E-04	n/a	4	0.395074865	23
REACTIVE BLUE 2	C0140012	6.87E-04	n/a	4	0.412252033	24
ADRENERGIC AGENTS	C0001637	8.07E-04	n/a	4	0.429429201	25
CHYMOPAPAIN	C0008736	8.07E-04	n/a	4	0.429429201	25
DICLOFENAC SODIUM	C0700583	8.07E-04	n/a	4	0.429429201	25
TENIDAP	C0076092	8.07E-04	n/a	4	0.429429201	25
AZELASTINE	C0052762	9.41E-04	n/a	4	0.446606369	26
CIS UROCANIC ACID	C3274548	9.41E-04	n/a	4	0.446606369	26
FERUMOXIDES	C0246249	1.26E-03	n/a	4	0.480960705	28
LAMBDA CARRAGEENAN	C0022971	1.26E-03	n/a	4	0.480960705	28
TAPI 1	C0294540	1.44E-03	n/a	4	0.498137873	29
FK778	C1142717	1.64E-03	n/a	4	0.515315041	30
BENZALKONIUM CHLORIDE	C0005026	1.64E-03	n/a	4	0.515315041	30
CC 4047	C1176324	1.64E-03	n/a	4	0.515315041	30
COOMASSIE BRILLIANT BLUE	C0056270	1.64E-03	n/a	4	0.515315041	30
TIGECYCLINE	C1260298	1.64E-03	n/a	4	0.515315041	30
BOLDINE	C0053908	1.64E-03	n/a	4	0.515315041	30
CEPHARANTHINE	C0055082	1.85E-03	n/a	4	0.532492209	31
SERPIN SUPERFAMILY	C0036763	2.09E-03	n/a	4	0.549669378	32
CHYMOSTATIN	C0055676	2.09E-03	n/a	4	0.549669378	32
SESAMOL	C0074406	2.09E-03	n/a	4	0.549669378	32
RECOMBINANT HUMAN THROMBOPOIETIN	C0392939	2.09E-03	n/a	4	0.549669378	32
TAPI 2	C0389500	2.09E-03	n/a	4	0.549669378	32
GLUCURONYL GLUCOSAMINE GLYCAN SULFATE	C0061450	2.09E-03	n/a	4	0.549669378	32
OPRELVEKIN	C0537670	2.35E-03	n/a	4	0.566846546	33
LORATADINE	C0065180	2.35E-03	n/a	4	0.566846546	33
USTEKINUMAB	C1608841	2.62E-03	n/a	4	0.584023714	34
CRANBERRY ALLERGENIC EXTRACT	C2740793	2.62E-03	n/a	4	0.584023714	34
TOSYLLYSINE CHLOROMETHYL KETONE	C0040495	2.62E-03	n/a	4	0.584023714	34
CRANBERRY PREPARATION	C0453273	2.62E-03	n/a	4	0.584023714	34
RECOMBINANT INTERLEUKIN 8	C1527432	2.92E-03	n/a	4	0.601200882	35
ZIRCONIUM OXIDE	C0078814	2.92E-03	n/a	4	0.601200882	35
IMMUNOGLOBULIN D	C0020843	2.92E-03	n/a	4	0.601200882	35
OLOPATADINE	C0527189	2.92E-03	n/a	4	0.601200882	35
METYRAPONE	C0025876	3.24E-03	n/a	4	0.61837805	36
IMMUNOL	C1517844	3.24E-03	n/a	4	0.61837805	36
FLUORO JADE	C1097415	3.96E-03	n/a	4	0.652732386	38
AG 126	C0526815	4.35E-03	n/a	4	0.669909554	39
ROFLUMILAST	C0965618	4.35E-03	n/a	4	0.669909554	39
AS 101	C0950716	4.35E-03	n/a	4	0.669909554	39

KETOTIFEN	C0022642	4.35E-03	n/a	4	0.669909554	39
RNA SYNTHESIS INHIBITORS	C0376567	4.77E-03	n/a	4	0.687086722	40
PKH 26	C0137168	4.77E-03	n/a	4	0.687086722	40
MILRINONE	C0128513	5.69E-03	n/a	4	0.721441058	42
COMPOUND 20	C0056212	5.69E-03	n/a	4	0.721441058	42
CASTANOSPERMINE	C0054849	5.69E-03	n/a	4	0.721441058	42
CUPRAMMONIUM CELLULOSE	C0056591	6.19E-03	n/a	4	0.738618226	43
CATHELICIDIN LL 37	C3891304	6.19E-03	n/a	4	0.738618226	43
THEAFLAVIN	C0076379	6.19E-03	n/a	4	0.738618226	43
PENTOXIFYLLINE	C0030899	6.19E-03	n/a	4	0.738618226	43
SODIUM SULFATE	C0074769	6.19E-03	n/a	4	0.738618226	43
DIFFERENTIATION INHIBITOR	C1511940	6.72E-03	n/a	4	0.755795394	44
ANTI ALLERGIC AGENTS	C0282555	6.72E-03	n/a	4	0.755795394	44
GLYCYRRHETINIC ACID	C0017986	7.28E-03	n/a	4	0.772972562	45
CETIRIZINE	C0055147	7.28E-03	n/a	4	0.772972562	45
EDETIC ACID	C0013618	7.87E-03	n/a	4	0.79014973	46
COLCEMIDE	C0813866	7.87E-03	n/a	4	0.79014973	46
PIRFENIDONE	C0298067	7.87E-03	n/a	4	0.79014973	46
RH1 BENZOQUINONE	C0764430	7.87E-03	n/a	4	0.79014973	46
CICAPROST	C0055705	8.48E-03	n/a	4	0.807326898	47
EFALIZUMAB	C1174995	8.48E-03	n/a	4	0.807326898	47
ETHACRYNIC ACID	C0014963	9.13E-03	n/a	4	0.824504066	48
9,10 ANTHRAQUINONE	C0050133	9.13E-03	n/a	4	0.824504066	48
PROTAMINE SULPHATE	C0033602	9.13E-03	n/a	4	0.824504066	48
TOCILIZUMAB	C1609165	9.13E-03	n/a	4	0.824504066	48
RECOMBINANT FIBROBLAST GROWTH FACTOR 1	C1522455	9.13E-03	n/a	4	0.824504066	48
WITHAFERIN A	C0078503	9.81E-03	n/a	4	0.841681234	49
COMPOUND 48 80	C0009574	9.81E-03	n/a	4	0.841681234	49
5 CHLORO 2 METHYL 4 ISOTHIAZOLIN 3 ONE	C0049104	1.97E-05	n/a	3	0.068708672	4
PHOTOALLERGEN	C0440108	1.97E-05	n/a	3	0.068708672	4
ONO 6818	C0963626	1.97E-05	n/a	3	0.068708672	4
IMMLG5521	C3254304	4.88E-05	n/a	3	0.08588584	5
HAMAMELITANNIN	C0297195	4.88E-05	n/a	3	0.08588584	5
LEUKADHERIN 1	C3851799	4.88E-05	n/a	3	0.08588584	5
PINANE	C0071079	4.88E-05	n/a	3	0.08588584	5
1,2,5 THIADIAZOLIDIN 3 ONE 1,1 DIOXIDE	C0966875	4.88E-05	n/a	3	0.08588584	5
TYRPHOSTIN AG 1288	C1519731	9.63E-05	n/a	3	0.103063008	6
AH 13205	C0215899	9.63E-05	n/a	3	0.103063008	6
TILMICOSIN	C0076681	1.66E-04	n/a	3	0.120240176	7
LIPOSOMAL CURCUMIN	C3898588	2.63E-04	n/a	3	0.137417344	8
PROLASTIN	C0701947	2.63E-04	n/a	3	0.137417344	8
DIMEMORFAN	C0058193	2.63E-04	n/a	3	0.137417344	8
FK 706	C0669496	3.89E-04	n/a	3	0.154594512	9
EMEDASTINE	C0064263	3.89E-04	n/a	3	0.154594512	9

NPC 15669	C0132918	3.89E-04	n/a	3	0.154594512	9
SAXATILIN	C1137277	5.49E-04	n/a	3	0.17177168	10
Y 24180	C0149072	5.49E-04	n/a	3	0.17177168	10
LASALOCID	C0023082	7.45E-04	n/a	3	0.188948849	11
CADEXOMER IODINE	C0054409	7.45E-04	n/a	3	0.188948849	11
PCB 104	C1453501	7.45E-04	n/a	3	0.188948849	11
ASPERGILLUS FUMIGATUS EXTRACT	C1445421	7.45E-04	n/a	3	0.188948849	11
DORNASE ALFA	C1135662	9.81E-04	n/a	3	0.206126017	12
ALPHA TYPE 1 POLARIZED DENDRITIC CELLS	C1831973	9.81E-04	n/a	3	0.206126017	12
TAPROSTENE	C0075812	9.81E-04	n/a	3	0.206126017	12
MZM	C0721501	9.81E-04	n/a	3	0.206126017	12
PHENYL BUTYRATE	C3848590	9.81E-04	n/a	3	0.206126017	12
SC 41930	C0074112	9.81E-04	n/a	3	0.206126017	12
CYCLOSPORIN H	C0056823	9.81E-04	n/a	3	0.206126017	12
REPARIXIN	C1506748	1.26E-03	n/a	3	0.223303185	13
ISIS 2302	C3495448	1.26E-03	n/a	3	0.223303185	13
INDOLICIDIN	C0123515	1.26E-03	n/a	3	0.223303185	13
METHYL SALICYLATE	C0066282	1.26E-03	n/a	3	0.223303185	13
MEDICARPIN	C0065857	1.26E-03	n/a	3	0.223303185	13
HARPAGOSIDE	C0062127	1.58E-03	n/a	3	0.240480353	14
SHOSAIKO TO	C1138004	1.58E-03	n/a	3	0.240480353	14
CV 3988	C0056625	1.58E-03	n/a	3	0.240480353	14
ONO AE1 329	C1138034	1.95E-03	n/a	3	0.257657521	15
FLUNISOLIDE	C0060501	1.95E-03	n/a	3	0.257657521	15
SOFALCONE	C0142971	1.95E-03	n/a	3	0.257657521	15
FLUNIXIN MEGLUMINE	C0060503	1.95E-03	n/a	3	0.257657521	15
N DODECANE	C0067942	2.37E-03	n/a	3	0.274834689	16
ACETATE	C0000975	2.37E-03	n/a	3	0.274834689	16
ASPHALT	C0052558	2.37E-03	n/a	3	0.274834689	16
ACETATES	C0000979	2.37E-03	n/a	3	0.274834689	16
TAUROLIDINE	C0144567	2.37E-03	n/a	3	0.274834689	16
WEB 2170	C0085001	2.37E-03	n/a	3	0.274834689	16
LORNOXICAM	C0055477	2.37E-03	n/a	3	0.274834689	16
FLUORANTHENE	C0060514	2.37E-03	n/a	3	0.274834689	16
GAMMA INTERFERON	C3539881	2.85E-03	n/a	3	0.292011857	17
COMPSTATIN	C0673968	2.85E-03	n/a	3	0.292011857	17
PIDOTIMOD	C0081002	2.85E-03	n/a	3	0.292011857	17
HUMAN NEUTROPHIL PEPTIDE 1	C0122620	2.85E-03	n/a	3	0.292011857	17
N METHYLFORMAMIDE	C0066373	3.37E-03	n/a	3	0.309189025	18
AMINONUCLEOSIDE	C0085791	3.37E-03	n/a	3	0.309189025	18
CARBOCYSTEINE	C0006992	3.37E-03	n/a	3	0.309189025	18
HEXAMINE SILVER	C0037128	3.37E-03	n/a	3	0.309189025	18
OXATOMIDE	C0134337	3.37E-03	n/a	3	0.309189025	18
AMETANTRONE	C0102923	3.37E-03	n/a	3	0.309189025	18

CREST	C2948667	3.95E-03	n/a	3	0.326366193	19
PLATONIN	C0137287	3.95E-03	n/a	3	0.326366193	19
CEP 11004	C1172647	3.95E-03	n/a	3	0.326366193	19
BUTANE	C0006474	3.95E-03	n/a	3	0.326366193	19
SUCCINIMIDE	C0075432	3.95E-03	n/a	3	0.326366193	19
ACECLOFENAC	C0050403	3.95E-03	n/a	3	0.326366193	19
HELENALIN	C0062169	3.95E-03	n/a	3	0.326366193	19
TETRAGASTRIN	C0039659	3.95E-03	n/a	3	0.326366193	19
TRP P 1	C0077413	3.95E-03	n/a	3	0.326366193	19
CORILAGIN	C0164514	3.95E-03	n/a	3	0.326366193	19
CLEMASTINE	C0008929	3.95E-03	n/a	3	0.326366193	19
OM 85 BV	C0163642	4.59E-03	n/a	3	0.343543361	20
SUCCINIMIDES	C0038623	4.59E-03	n/a	3	0.343543361	20
SR 11302	C0539455	4.59E-03	n/a	3	0.343543361	20
ANTIPYRETICS	C0003419	4.59E-03	n/a	3	0.343543361	20
IRRIGATION SOLUTIONS	C2746010	4.59E-03	n/a	3	0.343543361	20
CALCIUM PHOSPHATE CRYSTALS	C0368819	5.29E-03	n/a	3	0.360720529	21
POLYETHYLENE GLYCOL 8000	C0030776	5.29E-03	n/a	3	0.360720529	21
SU 6668	C0970963	5.29E-03	n/a	3	0.360720529	21
LIPOCALIN 1	C0078147	6.05E-03	n/a	3	0.377897697	22
MOLGRAMOSTIM	C0218633	6.05E-03	n/a	3	0.377897697	22
GOLD SODIUM THIOMALATE	C0018034	6.05E-03	n/a	3	0.377897697	22
MONOASPARTYL CHLORIN E6	C0245194	6.05E-03	n/a	3	0.377897697	22
TALL 104	C1832043	6.05E-03	n/a	3	0.377897697	22
DIPIVEFRIN	C0058415	6.05E-03	n/a	3	0.377897697	22
HUMAN NEUTROPHIL PEPTIDE 2	C0122159	6.05E-03	n/a	3	0.377897697	22
SODIUM IODATE	C0074742	6.05E-03	n/a	3	0.377897697	22
ONC201	C3827072	6.87E-03	n/a	3	0.395074865	23
OLEANDRIN	C0069397	6.87E-03	n/a	3	0.395074865	23
BONGKREKIC ACID	C0005982	6.87E-03	n/a	3	0.395074865	23
FR 167653	C0535734	6.87E-03	n/a	3	0.395074865	23
NAFAMOSTAT MESILATE	C0207682	6.87E-03	n/a	3	0.395074865	23
SULPROSTONE	C0075628	6.87E-03	n/a	3	0.395074865	23
RO 31 8425	C0140783	6.87E-03	n/a	3	0.395074865	23
ANGIOTENSIN II	C0003009	6.87E-03	n/a	3	0.395074865	23
DAMNACANTHAL	C0212399	6.87E-03	n/a	3	0.395074865	23
COMPOUND 32	C0258113	7.75E-03	n/a	3	0.412252033	24
ECHINOMYCIN	C0013508	7.75E-03	n/a	3	0.412252033	24
ANTIMETASTATIC AGENT	C1516021	7.75E-03	n/a	3	0.412252033	24
DALTEPARIN	C0206461	7.75E-03	n/a	3	0.412252033	24
CMT 3	C2979988	7.75E-03	n/a	3	0.412252033	24
MANIDIPINE	C0065656	7.75E-03	n/a	3	0.412252033	24
MURAMIDASE	C0026794	8.70E-03	n/a	3	0.429429201	25
RHUBARB PREPARATION	C0304561	8.70E-03	n/a	3	0.429429201	25

MRS 2179	C0674169	8.70E-03	n/a	3	0.429429201	25
CLI 095	C3179438	8.70E-03	n/a	3	0.429429201	25
NF 449	C0670517	8.70E-03	n/a	3	0.429429201	25
TERBINAFINE	C0076110	8.70E-03	n/a	3	0.429429201	25
CCI 779	C0796512	8.70E-03	n/a	3	0.429429201	25
PYCNOGENOL	C0072642	9.72E-03	n/a	3	0.446606369	26
4 COUMARIC ACID	C0048207	9.72E-03	n/a	3	0.446606369	26
GALIXIMAB	C1098262	9.72E-03	n/a	3	0.446606369	26
PYCNOGENOLS	C1616563	9.72E-03	n/a	3	0.446606369	26
XMD 8 92	C3252765	9.72E-03	n/a	3	0.446606369	26
LGD 1069	C0300770	9.72E-03	n/a	3	0.446606369	26
CECROPINS	C0597874	9.72E-03	n/a	3	0.446606369	26
DAK 16	C0112273	2.94E-04	n/a	2	0.034354336	2
FLECAINIDE ACETATE	C0086303	2.94E-04	n/a	2	0.034354336	2
1 PHENAZINECARBOXYLIC ACID	C0044583	2.94E-04	n/a	2	0.034354336	2
YM 47141	C0386137	2.94E-04	n/a	2	0.034354336	2
P 1507	C0297250	2.94E-04	n/a	2	0.034354336	2
REPANDOSIDE	C2934769	2.94E-04	n/a	2	0.034354336	2
KN 04	C0378618	8.66E-04	n/a	2	0.051531504	3
MUTEIN 6	C0538992	8.66E-04	n/a	2	0.051531504	3
L 658758	C0083171	8.66E-04	n/a	2	0.051531504	3
BDPT	C0644698	8.66E-04	n/a	2	0.051531504	3
ASCARIDOLE	C0052466	8.66E-04	n/a	2	0.051531504	3
AGRIMONIIN	C0050985	8.66E-04	n/a	2	0.051531504	3
6 HYDROXY 2,4,7 TRIMETHOXYPHENANTHRENE	C3253438	8.66E-04	n/a	2	0.051531504	3
LIPEGFILGRASTIM	C3640212	8.66E-04	n/a	2	0.051531504	3
TMI 1	C1456763	8.66E-04	n/a	2	0.051531504	3
HESPERIDIN METHYL CHALONE	C2350707	8.66E-04	n/a	2	0.051531504	3
MDL 101028	C0291761	8.66E-04	n/a	2	0.051531504	3
3' CHLORO 5,7 DIMETHOXYISOFLAVONE	C3491366	8.66E-04	n/a	2	0.051531504	3
RACTOPAMINE HYDROCHLORIDE	C0958044	1.72E-03	n/a	2	0.068708672	4
NITROFLURBIPROFEN	C0381496	1.72E-03	n/a	2	0.068708672	4
RECOMBINANT CTAP III	C1523977	1.72E-03	n/a	2	0.068708672	4
POLUDAN	C0071336	1.72E-03	n/a	2	0.068708672	4
AM6 36	C3177627	1.72E-03	n/a	2	0.068708672	4
ISIS 2922	C0950924	1.72E-03	n/a	2	0.068708672	4
PROSOM	C0722865	1.72E-03	n/a	2	0.068708672	4
RK I 123	C3714162	1.72E-03	n/a	2	0.068708672	4
POLYHEXAMETHYLENE BIGUANIDE	C0137794	1.72E-03	n/a	2	0.068708672	4
ACETYL AMVSEFLKQAWIENEEQEYVVQTVK	C0382612	1.72E-03	n/a	2	0.068708672	4
HWA 138	C0063039	1.72E-03	n/a	2	0.068708672	4
GLUTAMYL GLYCYL ARGINYL CHLOROMETHYL KET(	C0252794	1.72E-03	n/a	2	0.068708672	4
GLYCYL PROLYL ARGININE	C0061724	1.72E-03	n/a	2	0.068708672	4
SANTONIN RELATED COMPOUND 2	C3657379	1.72E-03	n/a	2	0.068708672	4

PD 098063	C0533563	1.72E-03	n/a	2	0.068708672	4
RP 55778	C0140881	1.72E-03	n/a	2	0.068708672	4
RHESUS ROTAVIRUS VACCINE	C0140933	1.72E-03	n/a	2	0.068708672	4
ON09310	C2353771	1.72E-03	n/a	2	0.068708672	4
AW00179	C3661253	1.72E-03	n/a	2	0.068708672	4
IRFI 042	C0971022	1.72E-03	n/a	2	0.068708672	4
BIIL 284	C0972319	1.72E-03	n/a	2	0.068708672	4
GLIOVIRIN	C0061326	1.72E-03	n/a	2	0.068708672	4
NPB001 05	C3253929	1.72E-03	n/a	2	0.068708672	4
BB 2275	C0257712	1.72E-03	n/a	2	0.068708672	4
RO 23 9424	C0073515	1.72E-03	n/a	2	0.068708672	4
IMUNOFAN	C0379865	1.72E-03	n/a	2	0.068708672	4
BVT173187	C3492858	1.72E-03	n/a	2	0.068708672	4
BECLOMETHASONE 17 MONOPROPIONATE	C0081729	1.72E-03	n/a	2	0.068708672	4
2 METHYL 4 ISOTHIAZOLIN 3 ONE	C0046343	1.72E-03	n/a	2	0.068708672	4
ALPHA HEXYLCINNAMALDEHYDE	C2935868	1.72E-03	n/a	2	0.068708672	4
FUSIDATE SODIUM	C0037509	2.84E-03	n/a	2	0.08588584	5
MALTOTETRAOSE	C0065615	2.84E-03	n/a	2	0.08588584	5
POLYMYXIN B SULFATE	C0001714	2.84E-03	n/a	2	0.08588584	5
BUTRIN	C0627897	2.84E-03	n/a	2	0.08588584	5
DIPHTHERIA TETANUS TOXOIDS	C0717715	2.84E-03	n/a	2	0.08588584	5
CSL 32	C3502702	2.84E-03	n/a	2	0.08588584	5
ISOBUTRIN	C0627895	2.84E-03	n/a	2	0.08588584	5
TRIFLUOPERAZINE HYDROCHLORIDE	C0304381	2.84E-03	n/a	2	0.08588584	5
YAKUCHINONE A	C0078676	2.84E-03	n/a	2	0.08588584	5
ONOPORDUM EXTRACT	C3486813	2.84E-03	n/a	2	0.08588584	5
SODIUM ASCORBYL PHOSPHATE	C1814855	2.84E-03	n/a	2	0.08588584	5
VINYL ETHER	C0078264	2.84E-03	n/a	2	0.08588584	5
SALICIN EXTRACT	C0874039	2.84E-03	n/a	2	0.08588584	5
PLANTAGO LANCEOLATA LEAF EXTRACT	C3256432	2.84E-03	n/a	2	0.08588584	5
AZADIRONE	C1453317	2.84E-03	n/a	2	0.08588584	5
CEPHAMYCINS	C0007736	2.84E-03	n/a	2	0.08588584	5
OHIOENSIN F	C2353874	2.84E-03	n/a	2	0.08588584	5
PAPCX	C0043958	2.84E-03	n/a	2	0.08588584	5
PARTHENIUM HYSTEROPHORUS EXTRACT	C1956630	2.84E-03	n/a	2	0.08588584	5
OLODATEROL	C2934193	2.84E-03	n/a	2	0.08588584	5
CT 1501R	C0243901	2.84E-03	n/a	2	0.08588584	5
F 4614	C0384949	2.84E-03	n/a	2	0.08588584	5
MABTHERA	C1314901	2.84E-03	n/a	2	0.08588584	5
HYPOTHIOCYANITE	C0122932	2.84E-03	n/a	2	0.08588584	5
SDZ MRL 953	C0141826	2.84E-03	n/a	2	0.08588584	5
CP 195543	C0676002	2.84E-03	n/a	2	0.08588584	5
DIGITOXIGENIN	C0012257	2.84E-03	n/a	2	0.08588584	5
CDP 571	C0910864	2.84E-03	n/a	2	0.08588584	5

CDP870	C1172157	2.84E-03	n/a	2	0.08588584	5
MR 889	C0066861	2.84E-03	n/a	2	0.08588584	5
PF 04418948	C3490773	2.84E-03	n/a	2	0.08588584	5
BUDLEIN A	C1957519	2.84E-03	n/a	2	0.08588584	5
CI 949	C0055696	2.84E-03	n/a	2	0.08588584	5
NAPROXEN N BUTYL NITRATE	C0673815	2.84E-03	n/a	2	0.08588584	5
SOLITHROMYCIN	C3655918	2.84E-03	n/a	2	0.08588584	5
PD 144795	C0381400	4.21E-03	n/a	2	0.103063008	6
SALICORTIN	C0676660	4.21E-03	n/a	2	0.103063008	6
3 DEOXYSAPPANCHALCONE	C2717294	4.21E-03	n/a	2	0.103063008	6
RETINYL RETINOATE	C2354879	4.21E-03	n/a	2	0.103063008	6
TYRPHOSTIN AG 126	C1435212	4.21E-03	n/a	2	0.103063008	6
PIMARIC ACID	C0071065	4.21E-03	n/a	2	0.103063008	6
OC000459	C3491265	4.21E-03	n/a	2	0.103063008	6
ALLERGINA	C1172086	4.21E-03	n/a	2	0.103063008	6
VTX 294	C3713619	4.21E-03	n/a	2	0.103063008	6
DIOSMECTITE	C0163748	4.21E-03	n/a	2	0.103063008	6
AH 23848B	C0101892	4.21E-03	n/a	2	0.103063008	6
PYRANOPROFEN	C0072652	4.21E-03	n/a	2	0.103063008	6
CV 159	C0056620	4.21E-03	n/a	2	0.103063008	6
ONO AP 324	C0764633	4.21E-03	n/a	2	0.103063008	6
LEX 032	C0381874	4.21E-03	n/a	2	0.103063008	6
SEPTILIN	C0074359	4.21E-03	n/a	2	0.103063008	6
TEA TREE OIL	C0216194	4.21E-03	n/a	2	0.103063008	6
IMMTHER	C0281671	4.21E-03	n/a	2	0.103063008	6
BRAIN NATRIURETIC PEPTIDE 45	C0107094	4.21E-03	n/a	2	0.103063008	6
CG100649	C4042958	4.21E-03	n/a	2	0.103063008	6
SC 58635	C0538926	4.21E-03	n/a	2	0.103063008	6
ELLA	C2927379	4.21E-03	n/a	2	0.103063008	6
CSY 0073	C3252427	4.21E-03	n/a	2	0.103063008	6
BRL 50481	C1566895	4.21E-03	n/a	2	0.103063008	6
T ACTIVIN	C0075727	4.21E-03	n/a	2	0.103063008	6
NNKOAC	C0216823	4.21E-03	n/a	2	0.103063008	6
PINE BARK EXTRACT	C0872909	4.21E-03	n/a	2	0.103063008	6
HOCHU EKKI TO	C0911380	4.21E-03	n/a	2	0.103063008	6
2,3 DIHYDROXYBENZOIC ACID	C0046544	4.21E-03	n/a	2	0.103063008	6
SC 10	C0173244	4.21E-03	n/a	2	0.103063008	6
CI 959	C0055699	4.21E-03	n/a	2	0.103063008	6
7 OME APBT	C4077637	5.82E-03	n/a	2	0.120240176	7
SB 656933	C3254571	5.82E-03	n/a	2	0.120240176	7
GLUCOSAMINE	C0017718	5.82E-03	n/a	2	0.120240176	7
NOC 7	C0759114	5.82E-03	n/a	2	0.120240176	7
CAELYX	C0701183	5.82E-03	n/a	2	0.120240176	7
ACTARIT	C0378042	5.82E-03	n/a	2	0.120240176	7

XANTHOTOXOL	C0207281	5.82E-03	n/a	2	0.120240176	7
ARACHIDONATE 12 LIPOXYGENASE INHIBITORS	C2936662	5.82E-03	n/a	2	0.120240176	7
INDOXAM	C0767967	5.82E-03	n/a	2	0.120240176	7
LONOMYCIN	C0065176	5.82E-03	n/a	2	0.120240176	7
VETERINARY DRUGS	C0376438	5.82E-03	n/a	2	0.120240176	7
HUMIRA	C1171255	5.82E-03	n/a	2	0.120240176	7
MGN1703	C2986392	5.82E-03	n/a	2	0.120240176	7
HUMAN LEUKOCYTE ELASTASE INHIBITOR	C0379422	5.82E-03	n/a	2	0.120240176	7
IRSOGLADINE MALEATE	C0091573	5.82E-03	n/a	2	0.120240176	7
ECABET SODIUM	C0378416	5.82E-03	n/a	2	0.120240176	7
CHLOROPROCAINE	C0055443	5.82E-03	n/a	2	0.120240176	7
MDX H210 ANTIBODY	C0281736	5.82E-03	n/a	2	0.120240176	7
BROMIDE ION	C3848571	5.82E-03	n/a	2	0.120240176	7
BERBERRUBINE	C0760606	5.82E-03	n/a	2	0.120240176	7
BEMIPARIN	C0916062	5.82E-03	n/a	2	0.120240176	7
CLADOSPORIUM HERBARUM EXTRACT	C1445427	5.82E-03	n/a	2	0.120240176	7
CNCM	C0633185	5.82E-03	n/a	2	0.120240176	7
ANGIOGEN	C1172713	5.82E-03	n/a	2	0.120240176	7
ARACHIDONATE 15 LIPOXYGENASE INHIBITORS	C2936663	5.82E-03	n/a	2	0.120240176	7
MUR NAC L ALA D ISOGLN	C0086659	5.82E-03	n/a	2	0.120240176	7
CHLOROACETIC ACID	C0008225	5.82E-03	n/a	2	0.120240176	7
DALFOPRISTIN / QUINUPRISTIN	C0526513	5.82E-03	n/a	2	0.120240176	7
PROCYANIDIN B1	C0890082	5.82E-03	n/a	2	0.120240176	7
HEXTEND	C1170317	5.82E-03	n/a	2	0.120240176	7
ADENOSINE A3 RECEPTOR AGONIST CF102	C0289969	5.82E-03	n/a	2	0.120240176	7
RU24858	C3852127	7.68E-03	n/a	2	0.137417344	8
CEPROTIN	C1949898	7.68E-03	n/a	2	0.137417344	8
DIPHENYLIODONIUM CHLORIDE	C0955122	7.68E-03	n/a	2	0.137417344	8
BEPOTASTINE	C2698371	7.68E-03	n/a	2	0.137417344	8
KELATORPHAN	C0064276	7.68E-03	n/a	2	0.137417344	8
ROMURTIDE	C0067246	7.68E-03	n/a	2	0.137417344	8
U 74389G	C0252126	7.68E-03	n/a	2	0.137417344	8
DENBUFYLLINE	C0057384	7.68E-03	n/a	2	0.137417344	8
CORN SILK PREPARATION	C0937769	7.68E-03	n/a	2	0.137417344	8
6 CHLORO 2',3' DIDEOXYGUANOSINE	C0381855	7.68E-03	n/a	2	0.137417344	8
2,2',4,6,6' PENTACHLOROBIPHENYL	C0962700	7.68E-03	n/a	2	0.137417344	8
SALIX	C1815865	7.68E-03	n/a	2	0.137417344	8
CALCOFLUOR WHITE	C0108160	7.68E-03	n/a	2	0.137417344	8
U 74389F	C0168928	7.68E-03	n/a	2	0.137417344	8
3,3',4',5 TETRACHLOROSALICYLANILIDE	C0046874	7.68E-03	n/a	2	0.137417344	8
PD IA	C0259505	7.68E-03	n/a	2	0.137417344	8
GSK2656157	C4044483	7.68E-03	n/a	2	0.137417344	8
PURINERGIC P2 RECEPTOR AGONISTS	C2936554	7.68E-03	n/a	2	0.137417344	8
ENNIATIN B	C0116183	7.68E-03	n/a	2	0.137417344	8

DESIRUDIN	C0378366	7.68E-03	n/a	2	0.137417344	8
JM 34	C0382648	7.68E-03	n/a	2	0.137417344	8
BXT 51072	C0757964	7.68E-03	n/a	2	0.137417344	8
BAY 73 6691	C1674022	7.68E-03	n/a	2	0.137417344	8
HUMULON	C0063031	7.68E-03	n/a	2	0.137417344	8
VERTICILLIN A	C0952065	7.68E-03	n/a	2	0.137417344	8
CREBANINE	C0056473	7.68E-03	n/a	2	0.137417344	8
NARASIN	C0068443	7.68E-03	n/a	2	0.137417344	8
SECURININE	C0074259	7.68E-03	n/a	2	0.137417344	8
SIM 010603	C3490196	7.68E-03	n/a	2	0.137417344	8
KJE	C0171293	7.68E-03	n/a	2	0.137417344	8
DIFID	C3490744	7.68E-03	n/a	2	0.137417344	8
XL184	C1710686	7.68E-03	n/a	2	0.137417344	8
CEVIMELINE	C0093437	7.68E-03	n/a	2	0.137417344	8
MVA 85A	C2933874	7.68E-03	n/a	2	0.137417344	8
JTE 607	C1172246	9.77E-03	n/a	2	0.154594512	9
PROXIGERMANIUM	C0081913	9.77E-03	n/a	2	0.154594512	9
ENPROSTIL	C0085191	9.77E-03	n/a	2	0.154594512	9
WOBE MUGOS E	C0935809	9.77E-03	n/a	2	0.154594512	9
TRIAZOLODIAZEPINE	C0245118	9.77E-03	n/a	2	0.154594512	9
TOCOPHEROL ACETATE	C0078373	9.77E-03	n/a	2	0.154594512	9
POLYPHENON E	C0917976	9.77E-03	n/a	2	0.154594512	9
PENTAMIDINE ISETHIONATE	C0030864	9.77E-03	n/a	2	0.154594512	9
FELINE INFECTIOUS PERITONITIS VIRUS VACCINE	C0310728	9.77E-03	n/a	2	0.154594512	9
ORAL POLIOVIRUS VACCINE	C0032375	9.77E-03	n/a	2	0.154594512	9
BWA868C	C2350218	9.77E-03	n/a	2	0.154594512	9
2 DEOXY 2,3 DEHYDRO N ACETYLNEURAMINIC ACI	C0046054	9.77E-03	n/a	2	0.154594512	9
ACETOPYRROTHINE	C0050460	9.77E-03	n/a	2	0.154594512	9
ELASTATINAL	C0059010	9.77E-03	n/a	2	0.154594512	9
APRICOXIB	C1737955	9.77E-03	n/a	2	0.154594512	9
MC1575	C3712842	9.77E-03	n/a	2	0.154594512	9
CAY 10441	C3179460	9.77E-03	n/a	2	0.154594512	9
SOPHORIDINE	C0143094	9.77E-03	n/a	2	0.154594512	9
ZOMETA	C0939788	9.77E-03	n/a	2	0.154594512	9
ANASTELLIN	C0965520	9.77E-03	n/a	2	0.154594512	9
OXIDIZED CELLULOSE	C0007649	9.77E-03	n/a	2	0.154594512	9
PHLOXINE	C0250317	9.77E-03	n/a	2	0.154594512	9
ANTITHROMBIN III	C0003438	9.77E-03	n/a	2	0.154594512	9
ALLOFERON	C1721989	9.77E-03	n/a	2	0.154594512	9
TUMOR VASCULATURE TARGETED TUMOR NECROS	C1143119	9.77E-03	n/a	2	0.154594512	9
ACANTHOIC ACID	C0390661	9.77E-03	n/a	2	0.154594512	9

Pharmacological Substances (Genomatix) enriched in H^{WT}+2HG

ATRA-related Gene signature
VD-related Gene Signature

Pharmacological substance	macological substan	P-value	Adjusted p-value	# Genes (observed)	# Genes (expected)	# Genes (total)
TRETINOIN	C0040845	1.18E-11	n/a	32	8.268602265	989
RETINOIDS	C0035339	1.21E-05	n/a	20	6.822223911	816
DEXAMETHASONE	C0011777	1.35E-04	n/a	13	3.89602493	466
INTERFERON TYPE I	C0021743	4.40E-05	n/a	11	2.524891693	302
ENDOPEPTIDASES	C0030946	7.42E-05	n/a	11	2.675381926	320
FUCOIDAN	C0060794	1.02E-07	n/a	10	1.061792202	127
CYCLOHEXIMIDE	C0010572	1.24E-03	n/a	10	3.1602949	378
UROKINASE	C0042071	3.97E-05	n/a	9	1.647031998	197
ASPIRIN	C0004057	9.34E-05	n/a	9	1.839325074	220
RECOMBINANT MACROPHAGE COLONY STIMULAT	C1524095	2.58E-05	n/a	8	1.195561298	143
1,2 DIACYLGLYCEROL	C0043791	2.34E-03	n/a	8	2.332598617	279
DIGLYCERIDES	C0012262	2.45E-03	n/a	8	2.349319754	281
IONOMYCIN	C0022020	3.30E-03	n/a	8	2.466367713	295
CILOSTAZOL	C0055729	2.73E-05	n/a	7	0.877859694	105
SERINE PROTEINASE INHIBITORS	C0036733	4.46E-04	n/a	7	1.371133237	164
LINOLEIC ACID	C0023749	4.80E-04	n/a	7	1.387854374	166
CALCITRIOL	C0006674	5.54E-04	n/a	7	1.421296648	170
INTERLEUKIN 12	C0123759	5.73E-04	n/a	7	1.429657217	171
SB 203580	C0297666	8.03E-04	n/a	7	1.513262902	181
ATORVASTATIN	C0286651	2.17E-03	n/a	7	1.797522232	215
SERPINS	C0036764	3.90E-03	n/a	7	1.998175876	239
GANGLIOSIDES	C0017082	5.66E-03	n/a	7	2.140305541	256
U 0126	C0755562	6.94E-03	n/a	7	2.223911226	266
BORTEZOMIB	C1176309	7.07E-03	n/a	7	2.232271794	267
GLUCANS	C0017696	8.14E-05	n/a	6	0.719008893	86
WY 14,643	C0525129	1.18E-04	n/a	6	0.769172304	92
PROLINEDITHIOCARBAMATE	C0084183	1.18E-04	n/a	6	0.769172304	92
TELMISARTAN	C0248719	1.50E-04	n/a	6	0.802614578	96
RECOMBINANT INTERFERON ALPHA INDUCIBLE PR	C1522712	1.88E-04	n/a	6	0.836056852	100
ARTESUNATE	C0052432	1.88E-04	n/a	6	0.836056852	100
VALSARTAN	C0216784	2.33E-04	n/a	6	0.869499126	104
PROTEASE INHIBITORS	C0033607	2.45E-04	n/a	6	0.877859694	105
FTY 720	C0388088	8.31E-04	n/a	6	1.103595044	132
PLASMINOGEN ACTIVATOR	C0032144	1.17E-03	n/a	6	1.178840161	141
INDOMETHACIN	C0021246	1.90E-03	n/a	6	1.29588812	155
HEMIN	C0018988	2.03E-03	n/a	6	1.312609257	157
CERULETIDE	C0006639	2.52E-03	n/a	6	1.371133237	164
RECOMBINANT ALPHA CHEMOKINE	C1527035	2.84E-03	n/a	6	1.404575511	168
CARNITINE	C0007258	3.19E-03	n/a	6	1.438017785	172
VITAMIN D3	C3265062	3.29E-03	n/a	6	1.446378354	173

MAGNESIUM CATION	C2346927	3.38E-03	n/a	6	1.454738922	174
DEXTRAN SULFATE SODIUM	C0079225	4.21E-03	n/a	6	1.52162347	182
CHOLECALCIFEROL	C0008318	4.33E-03	n/a	6	1.529984039	183
PREDNISOLONE	C0032950	5.88E-03	n/a	6	1.630310861	195
ALLERGEN	C3274161	6.02E-03	n/a	6	1.63867143	196
DINOPROSTONE	C0012472	9.31E-03	n/a	6	1.797522232	215
PHOSPHATASE INHIBITOR	C0597217	9.72E-03	n/a	6	1.814243369	217
AM 580	C0102885	3.14E-06	n/a	5	0.234095919	28
WEB 2086	C0078460	3.77E-06	n/a	5	0.242456487	29
CUPRAMMONIUM CELLULOSE	C0056591	2.79E-05	n/a	5	0.359504446	43
ELASTASE INHIBITOR	C0596481	3.89E-05	n/a	5	0.384586152	46
RECOMBINANT GRANULOCYTE COLONY STIMULAT	C0376562	5.85E-05	n/a	5	0.418028426	50
DIHYDROETHIDIUM	C0644810	5.85E-05	n/a	5	0.418028426	50
EFAVIRENZ	C0674428	1.10E-04	n/a	5	0.476552406	57
4 BETA AMINOETHYLBENZOLSULFOFLUORIDE	C0047895	1.41E-04	n/a	5	0.501634111	60
CIGLITAZONE	C0055721	1.92E-04	n/a	5	0.535076385	64
RHEIN	C0073177	2.07E-04	n/a	5	0.543436954	65
ILOPROST	C0079594	2.74E-04	n/a	5	0.576879228	69
CYSTEINYL LEUKOTRIENE	C0675175	5.14E-04	n/a	5	0.660484913	79
ADALIMUMAB	C1122087	7.58E-04	n/a	5	0.719008893	86
CYTOCHALASIN B	C0010737	1.30E-03	n/a	5	0.810975146	97
TANSHINONE II A	C0897640	1.43E-03	n/a	5	0.827696283	99
BUDESONIDE	C0054201	2.02E-03	n/a	5	0.894580831	107
MERBROMIN	C0025397	2.10E-03	n/a	5	0.9029414	108
BETA GLUCANS	C1134651	2.46E-03	n/a	5	0.936383674	112
PROTAMINES	C0033603	4.37E-03	n/a	5	1.07015277	128
CHENODEOXYCHOLIC ACID	C0008024	4.52E-03	n/a	5	1.078513339	129
BAFILOMYCIN A1	C0052919	4.52E-03	n/a	5	1.078513339	129
TROGLITAZONE	C0245514	5.82E-03	n/a	5	1.145397887	137
KAEMPFEROLS	C1257968	7.15E-03	n/a	5	1.203921867	144
SEVOFLURANE	C0074414	7.15E-03	n/a	5	1.203921867	144
MINOCYCLINE	C0026187	7.15E-03	n/a	5	1.203921867	144
RITUXIMAB	C0393022	7.15E-03	n/a	5	1.203921867	144
KETAMINE	C0022614	8.01E-03	n/a	5	1.237364141	148
CPG ODN	C0914671	8.93E-03	n/a	5	1.270806415	152
PROTEIN KINASE INHIBITORS	C1449702	9.67E-03	n/a	5	1.29588812	155
TEPOXALIN	C0145162	5.88E-06	n/a	4	0.125408528	15
NCX 4016	C0950966	8.11E-05	n/a	4	0.234095919	28
ABCIXIMAB	C0288672	2.22E-04	n/a	4	0.300980467	36
ANTI INFLAMMATORY AGENTS	C0003209	4.44E-04	n/a	4	0.359504446	43
CETIRIZINE	C0055147	5.29E-04	n/a	4	0.376225583	45
RADIOIMMUNOCONJUGATES	C0243019	6.78E-04	n/a	4	0.401307289	48
PIROXICAM	C0031990	9.88E-04	n/a	4	0.443110131	53
TRANS RESVERATROL	C1101322	1.06E-03	n/a	4	0.4514707	54

OK 432	C0918027	1.14E-03	n/a	4	0.459831269	55
THERAPEUTIC CORYNEBACTERIUM PARVUM	C1522468	1.30E-03	n/a	4	0.476552406	57
TOFACITINIB	C2930696	1.30E-03	n/a	4	0.476552406	57
DIPHENYLENEIODIUM CHLORIDE	C0243281	1.39E-03	n/a	4	0.484912974	58
7 AMINOACTINOMYCIN D	C0049832	1.67E-03	n/a	4	0.50999468	61
AGLYCONE	C0304518	1.89E-03	n/a	4	0.526715817	63
PYRROLIDINE DITHIOCARBAMATE	C0163305	2.92E-03	n/a	4	0.593600365	71
EPLERENONE	C0961485	3.08E-03	n/a	4	0.601960933	72
PROBUCOL	C0033215	3.74E-03	n/a	4	0.635403207	76
OLIGOADENYLATE	C0133819	4.30E-03	n/a	4	0.660484913	79
LOW MOLECULAR WEIGHT HEPARIN	C0019139	4.30E-03	n/a	4	0.660484913	79
DYNE VETERINARY DRUG	C0308914	4.91E-03	n/a	4	0.685566619	82
GROWTH INHIBITORS	C0018282	5.12E-03	n/a	4	0.693927187	83
AMLODIPINE	C0051696	5.34E-03	n/a	4	0.702287756	84
SPIRONOLACTONE	C0037982	5.81E-03	n/a	4	0.719008893	86
U 44619	C0917834	6.30E-03	n/a	4	0.73573003	88
LUTEIN	C0043328	6.81E-03	n/a	4	0.752451167	90
NITRITE ION	C3848529	7.93E-03	n/a	4	0.785893441	94
DIPHENYLENEIODONIUM	C0058381	8.22E-03	n/a	4	0.794254009	95
FT011	C3501043	5.62E-06	n/a	3	0.041802843	5
SALIX	C1815865	3.09E-05	n/a	3	0.066884548	8
Y 24180	C0149072	6.54E-05	n/a	3	0.083605685	10
MANOALIDE	C0065711	1.18E-04	n/a	3	0.100326822	12
LY 255283	C0083286	1.94E-04	n/a	3	0.117047959	14
TRYPSIN	C0041236	2.40E-04	n/a	3	0.125408528	15
TRIFLUSAL	C0077126	2.40E-04	n/a	3	0.125408528	15
U 75302	C0077643	2.94E-04	n/a	3	0.133769096	16
LORNOXICAM	C0055477	2.94E-04	n/a	3	0.133769096	16
13 HYDROXYOCTADECADIENOIC ACID	C0090346	3.55E-04	n/a	3	0.142129665	17
FILGRASTIM	C0210630	4.23E-04	n/a	3	0.150490233	18
OZAGREL	C0069392	4.23E-04	n/a	3	0.150490233	18
CINNAMATES	C0008801	4.23E-04	n/a	3	0.150490233	18
CINNAMATE	C3496185	4.23E-04	n/a	3	0.150490233	18
MENHADEN OIL	C0065939	5.00E-04	n/a	3	0.158850802	19
TRIPAM CYS SER	C0091366	6.78E-04	n/a	3	0.175571939	21
NEDOCROMIL SODIUM	C0886658	7.80E-04	n/a	3	0.183932507	22
RO 31 8425	C0140783	8.91E-04	n/a	3	0.192293076	23
R 59949	C0072922	1.01E-03	n/a	3	0.200653644	24
RECOMBINANT GAMMA CHEMOKINE	C1527136	1.14E-03	n/a	3	0.209014213	25
ONO 1301	C0538030	1.14E-03	n/a	3	0.209014213	25
CHYMOPAPAIN	C0008736	1.14E-03	n/a	3	0.209014213	25
PYRROLIDINEDITHIOCARBAMATE	C1313719	1.29E-03	n/a	3	0.217374781	26
METALLOPORPHYRINS	C0025541	1.29E-03	n/a	3	0.217374781	26
CORM 2	C1120350	1.44E-03	n/a	3	0.22573535	27

ROFECOXIB	C0762662	1.44E-03	n/a	3	0.22573535	27
COPPER SULFATE	C0056301	1.60E-03	n/a	3	0.234095919	28
EFLORNITHINE	C0002260	1.60E-03	n/a	3	0.234095919	28
PROCATEROL	C0072032	1.60E-03	n/a	3	0.234095919	28
CHEMOKINE RECEPTOR ANTAGONIST	C1880030	1.60E-03	n/a	3	0.234095919	28
IRON DEXTRAN COMPLEX	C0022092	1.77E-03	n/a	3	0.242456487	29
TIROFIBAN	C0247025	1.77E-03	n/a	3	0.242456487	29
EPICATECHIN 3 GALLATE	C0059422	1.96E-03	n/a	3	0.250817056	30
CILENGITIDE	C0971473	1.96E-03	n/a	3	0.250817056	30
BOLDINE	C0053908	1.96E-03	n/a	3	0.250817056	30
CC 4047	C1176324	1.96E-03	n/a	3	0.250817056	30
SARPOGRELATE	C0286592	2.16E-03	n/a	3	0.259177624	31
CARBON BLACK	C0007010	2.16E-03	n/a	3	0.259177624	31
SERPIN SUPERFAMILY	C0036763	2.36E-03	n/a	3	0.267538193	32
HUMAN THROMBIN	C2369149	2.36E-03	n/a	3	0.267538193	32
RECOMBINANT HUMAN THROMBOPOIETIN	C0392939	2.36E-03	n/a	3	0.267538193	32
IMPERATORIN	C0063435	2.36E-03	n/a	3	0.267538193	32
RILPIVIRINE	C1566826	2.36E-03	n/a	3	0.267538193	32
INDO 1	C0063477	2.36E-03	n/a	3	0.267538193	32
GLUCURONYL GLUCOSAMINE GLYCAN SULFATE	C0061450	2.36E-03	n/a	3	0.267538193	32
CICLESONIDE	C0907850	2.58E-03	n/a	3	0.275898761	33
CRANBERRY PREPARATION	C0453273	2.82E-03	n/a	3	0.28425933	34
EPTIFIBATIDE	C0253563	2.82E-03	n/a	3	0.28425933	34
CRANBERRY ALLERGENIC EXTRACT	C2740793	2.82E-03	n/a	3	0.28425933	34
ZINC OXIDE	C0043491	3.06E-03	n/a	3	0.292619898	35
TERFENADINE	C0085173	3.06E-03	n/a	3	0.292619898	35
LINAGLIPTIN	C2746078	3.06E-03	n/a	3	0.292619898	35
MK 886	C0128640	3.06E-03	n/a	3	0.292619898	35
RECOMBINANT INTERLEUKIN 8	C1527432	3.06E-03	n/a	3	0.292619898	35
ONE ALPHA	C0591905	3.88E-03	n/a	3	0.317701604	38
BENZYL ALCOHOL	C0005100	3.88E-03	n/a	3	0.317701604	38
SCOPARONE	C0074191	4.18E-03	n/a	3	0.326062172	39
KETOTIFEN	C0022642	4.18E-03	n/a	3	0.326062172	39
ECULIZUMAB	C1541483	4.18E-03	n/a	3	0.326062172	39
LOXORIBINE	C0209660	4.49E-03	n/a	3	0.334422741	40
RNA SYNTHESIS INHIBITORS	C0376567	4.49E-03	n/a	3	0.334422741	40
STALLIMYCIN	C0244119	4.49E-03	n/a	3	0.334422741	40
MEASLES VACCINE	C0025010	4.49E-03	n/a	3	0.334422741	40
RIMONABANT	C1142933	6.25E-03	n/a	3	0.376225583	45
ALLERGENIC EXTRACT	C0440016	7.06E-03	n/a	3	0.39294672	47
EFALIZUMAB	C1174995	7.06E-03	n/a	3	0.39294672	47
SAPORIN	C0244777	7.92E-03	n/a	3	0.409667857	49
COMPOUND 48 80	C0009574	7.92E-03	n/a	3	0.409667857	49
ARACHIDONATE 5 LIPOXYGENASE INHIBITORS	C1289971	7.92E-03	n/a	3	0.409667857	49

LATANOPROST	C0090306	8.38E-03	n/a	3	0.418028426	50
GLIOTOXIN	C0017641	8.38E-03	n/a	3	0.418028426	50
SODIUM SALICYLATE	C0037549	8.85E-03	n/a	3	0.426388994	51
NATALIZUMAB	C1172734	9.34E-03	n/a	3	0.434749563	52
GAMMA TOCOPHEROL	C0017054	9.84E-03	n/a	3	0.443110131	53
CPENSPM	C0247670	2.06E-04	n/a	2	0.025081706	3
AM6 36	C3177627	4.11E-04	n/a	2	0.033442274	4
RS 504393	C3657798	4.11E-04	n/a	2	0.033442274	4
CHLORELLA 11 PEPTIDE	C2973755	4.11E-04	n/a	2	0.033442274	4
LEUKADHERIN 1	C3851799	6.81E-04	n/a	2	0.041802843	5
BIGELOVIN	C0390315	6.81E-04	n/a	2	0.041802843	5
KURARIDIN	C2974938	6.81E-04	n/a	2	0.041802843	5
HYPERACUTE PANCREATIC CANCER VACCINE	C1831791	6.81E-04	n/a	2	0.041802843	5
IDESCARPIN	C3713485	6.81E-04	n/a	2	0.041802843	5
CCR5 RECEPTOR ANTAGONISTS	C3850102	1.02E-03	n/a	2	0.050163411	6
SC 58635	C0538926	1.02E-03	n/a	2	0.050163411	6
OC000459	C3491265	1.02E-03	n/a	2	0.050163411	6
2 OXOTHIAZOLIDINE 4 CARBOXYLATE	C0124869	1.02E-03	n/a	2	0.050163411	6
PINE BARK EXTRACT	C0872909	1.02E-03	n/a	2	0.050163411	6
ACETYLAMINOPHENYLARSINE OXIDE	C0642476	1.02E-03	n/a	2	0.050163411	6
WX UK1	C1453900	1.02E-03	n/a	2	0.050163411	6
GR144053F	C1258963	1.42E-03	n/a	2	0.05852398	7
NOC 7	C0759114	1.42E-03	n/a	2	0.05852398	7
SKI 306X	C1137701	1.42E-03	n/a	2	0.05852398	7
INDOXAM	C0767967	1.42E-03	n/a	2	0.05852398	7
EICOSAPENTAENOATE	C1370123	1.42E-03	n/a	2	0.05852398	7
REOPRO	C0592046	1.42E-03	n/a	2	0.05852398	7
PF 956980	C2975489	1.42E-03	n/a	2	0.05852398	7
SRPIN340	C3712368	1.42E-03	n/a	2	0.05852398	7
CEPROTIN	C1949898	1.88E-03	n/a	2	0.066884548	8
DIPHENYLIODONIUM CHLORIDE	C0955122	1.88E-03	n/a	2	0.066884548	8
DENBUFYLLINE	C0057384	1.88E-03	n/a	2	0.066884548	8
NITROASPIRIN	C0531264	1.88E-03	n/a	2	0.066884548	8
2,2',4,6,6' PENTACHLOROBIPHENYL	C0962700	1.88E-03	n/a	2	0.066884548	8
GSK2606414	C3500976	1.88E-03	n/a	2	0.066884548	8
DIMEMORFAN	C0058193	1.88E-03	n/a	2	0.066884548	8
PROXIGERMANIUM	C0081913	2.40E-03	n/a	2	0.075245117	9
WOBE MUGOS E	C0935809	2.40E-03	n/a	2	0.075245117	9
LYSO 7	C3714050	2.40E-03	n/a	2	0.075245117	9
NSC 651016	C0676196	2.40E-03	n/a	2	0.075245117	9
N ACETYLCYSTEINAMIDE	C1452654	2.40E-03	n/a	2	0.075245117	9
RAMATROBAN	C0753318	2.98E-03	n/a	2	0.083605685	10
WOBENZYM	C0078510	2.98E-03	n/a	2	0.083605685	10
ONO 5046	C0133951	2.98E-03	n/a	2	0.083605685	10

ANTISTASIN	C0052098	3.63E-03	n/a	2	0.091966254	11
ABIETIC ACID	C0050365	3.63E-03	n/a	2	0.091966254	11
GAMBOGE	C0301271	3.63E-03	n/a	2	0.091966254	11
YCG 063	C2974353	3.63E-03	n/a	2	0.091966254	11
PYRILAMINE MALEATE	C0282330	3.63E-03	n/a	2	0.091966254	11
BAY X 1005	C0656891	3.63E-03	n/a	2	0.091966254	11
VIOXX	C0876768	3.63E-03	n/a	2	0.091966254	11
PCB 104	C1453501	3.63E-03	n/a	2	0.091966254	11
PHENYLAMIL	C0070664	4.33E-03	n/a	2	0.100326822	12
PHENYL BUTYRATE	C3848590	4.33E-03	n/a	2	0.100326822	12
CATUMAXOMAB	C1831854	4.33E-03	n/a	2	0.100326822	12
ETHYL PROTOCATECHUATE	C0059781	4.33E-03	n/a	2	0.100326822	12
3 MORPHOLINO SYDNONIMINE MONOHYDROCHI	C0951117	4.33E-03	n/a	2	0.100326822	12
ISIS 2302	C3495448	5.09E-03	n/a	2	0.108687391	13
EOSINOPHIL DERIVED NEUROTOXIN	C0059409	5.09E-03	n/a	2	0.108687391	13
PHOSPHORUS 32	C0851287	5.09E-03	n/a	2	0.108687391	13
2,5 DI TERT BUTYLHYDROQUINONE	C0045546	5.09E-03	n/a	2	0.108687391	13
POLY L LACTIC ACID	C0525166	5.09E-03	n/a	2	0.108687391	13
CYL 26Z	C1613957	5.09E-03	n/a	2	0.108687391	13
CP 105696	C0289246	5.09E-03	n/a	2	0.108687391	13
HEME ARGINATE	C0062212	5.90E-03	n/a	2	0.117047959	14
TAMIBAROTENE	C1567753	5.90E-03	n/a	2	0.117047959	14
PICLAMILAST	C0295451	5.90E-03	n/a	2	0.117047959	14
RWJ 67657	C0910100	5.90E-03	n/a	2	0.117047959	14
CV 3988	C0056625	5.90E-03	n/a	2	0.117047959	14
RP 73401	C0295452	6.77E-03	n/a	2	0.125408528	15
SMALLPOX VACCINE	C0037355	6.77E-03	n/a	2	0.125408528	15
APO 866	C1879518	6.77E-03	n/a	2	0.125408528	15
LOVAZA	C0912903	6.77E-03	n/a	2	0.125408528	15
LG 268	C0965513	6.77E-03	n/a	2	0.125408528	15
RO 41 5253	C0250032	6.77E-03	n/a	2	0.125408528	15
COD LIVER OIL	C0009213	7.70E-03	n/a	2	0.133769096	16
RECOMBINANT GRANULOCYTE MACROPHAGE COI	C0376563	7.70E-03	n/a	2	0.133769096	16
FRUCTOOLIGOSACCHARIDE	C0873033	7.70E-03	n/a	2	0.133769096	16
WEB 2170	C0085001	7.70E-03	n/a	2	0.133769096	16
ADENOSINE DIPHOSPHATE RECEPTOR ANTAGONIS	C2936586	7.70E-03	n/a	2	0.133769096	16
ISOANGUSTONE A	C2936061	7.70E-03	n/a	2	0.133769096	16
JTE 522	C0670957	7.70E-03	n/a	2	0.133769096	16
POLYAMINE ANALOGUE	C1709590	8.68E-03	n/a	2	0.142129665	17
INT 747	C2830015	8.68E-03	n/a	2	0.142129665	17
SPIRAMYCIN	C0037962	8.68E-03	n/a	2	0.142129665	17
MAPRACORAT	C3252363	8.68E-03	n/a	2	0.142129665	17
4 BROMOPHENACYL BROMIDE	C0048134	9.71E-03	n/a	2	0.150490233	18
POLY ICLC	C0071359	9.71E-03	n/a	2	0.150490233	18

DIRECT THROMBIN INHIBITORS	C3541936	9.71E-03	n/a	2	0.150490233	18
CB 3717	C0054911	9.71E-03	n/a	2	0.150490233	18
BACTENECIN	C0052891	9.71E-03	n/a	2	0.150490233	18
CORN TRYPSIN INHIBITOR	C0077421	9.71E-03	n/a	2	0.150490233	18
GAMMA THROMBIN	C0017052	9.71E-03	n/a	2	0.150490233	18
