# Supplementary material for: Activation of Vitamin D Receptor Pathway Enhances Differentiating Capacity in Acute Myeloid Leukemia with Isocitrate Dehydrogenase Mutations
Source: Cancers (Basel). 2021 Oct 19;13(20):5243. doi: 10.3390/cancers13205243 (PMC8533831; doi:10.3390/cancers13205243)
Supplement: Supplementary file 1 [file cancers-13-05243-s001.zip › Table S3.pdf]

Table S3 : ATRA- and VD-responsive genes enriched in IDHMUT-AML and HL60WT +2HG

ATRA and VD responsive genes	ATRA responsive genes				VD responsive genes
ITGAM	CD180	HOXD8	BTBD11	IRF8	SLC2A3
ICAM1	NPDC1	NCF2	HOXB2	HOXA9	KLF10
CEBPE	PPARD	BCL6	CSF3	GPRC5A	TNF
THEMIS2	OTUD7B	NME3	IL6ST	HOXA3	P2RX7
MYCN	IFIT5	ZSCAN4	CD33	BST1	MPO
RXRA	ANXA2	KLF5	DHRS3	SMAD5	VIM
ITGAX	ADIG	MEIS1	SCPEP1	IL5RA	CD86
	ITGA5	HNF1B	POU4F1	BCL2A1	TNFAIP3
	IFIT1	FGR	RBP4	CD38	CD80
	MIR223	TNFAIP2	IL6R	CEBPA	TXNIP
	CLTCL1	CEACAM6	HOXB6	HK3	SELL
	TAGLN	CCR6	FUT4	CYP4F2	CXCL8
	CCDC26	PALD1	RAB39A	DHRS9	NFATC2
	HOXB1	JUN	MYCL	ASB2	CD68
	BLNK	CD1D	CHD7	CNTN4	CCR7
	CCNA1	NOG	SOX14	CCND1	MNDA
	MGAT5	KRT80	HSD17B6	IGFBP2	ITGA6
	SLC26A3	SOX3	HIC1	GALM	
	HBZ	RBP1	GRN	ZRSR1	
	COL8A2	APBB1IP	HOXA4	S100A9	
	LMNA	CXCL16	HES1	CCRL2	
	HOXC6	DUOX2	CFD	EXOC6B	
	SPZ1	FGF8	CBX2	IL23R	
	HPR	ZBTB16	RET	KLF4	
	RBP7	NEDD9	PBX3	THBD	
	IER3	TGFBI	ENO2	MECOM	
	PRICKLE1	SMPD3	TLR3	FIGF	
	WNT5B	CSF3R	ANPEP	ZSCAN10	
	ZNF536	SERPINF1	CCL24	CYP2C18	
	PRAME	CD300LB	HOXA5	CD14	
		PTGDS			
