# Supplementary material for: Activation of Vitamin D Receptor Pathway Enhances Differentiating Capacity in Acute Myeloid Leukemia with Isocitrate Dehydrogenase Mutations
Source: Cancers (Basel). 2021 Oct 19;13(20):5243. doi: 10.3390/cancers13205243 (PMC8533831; doi:10.3390/cancers13205243)
Supplement: Supplementary file 1 [file cancers-13-05243-s001.zip › Table S4.pdf]

Table S4 : Gene signatures used for GSEA analysis

LATE_VD/VDR activation pathway (Warwick et al., 2021)						PID_RXR_VDR_PATHWAY (RXR and RAR heterodimerization with other nuclear receptor)
MMP1	NWD1	KIAA1211L	CCR1	RP11-4L24.4	TGFB2	ABCA1
LYPLA1P2	TMEM37	RP11-133K1.12	C5AR2	PRLR	HSH2D	BCL2
AC011290.4	HBEGF	GJD3	OCEL1	SLC31A1	CIB3	FAM120B
FAM182A	NCF1	FAM179A	LGALS9	RP4-737E23.6	MFAP4	MED1
CYP24A1	AGAP13P	RAPGEF3	RET	SCARB1	ABCA1	NCOA1
CAMP	RP11-120M18.2	LRRC3B	PLEKHO1	TMC6	SORD2P	NCOR2
RFPL2	AGAP7P	LPGAT1	LRP1	FAM134B	EPHB6	NR1H2
MSI1	RP11-69J7.1	PPARGC1B	B3GNT8	PPARG	RP11-849F2.9	NR1H3
RP11-605F22.2	DAPK2	TMEM52B	GLIPR1	IPCEF1	RGMA	NR1H4
LGALS9C	CARD11	NINJ1	AC064834.1	HTT	SHANK1	NR4A1
NOVA2	ASAP2	RP11-517P14.2	FGD3	RP5-862P8.2	TMEM255A	PPARA
SULT1C2	SPATA13	C1orf132	LNX1	ANKRD10	CSPG4	PPARD
SHE	ELL2	QPCT	SLC7A2	ZNF608	ADGRG5	PPARG
C3orf52	RP5-1007H16.1	LINC00659	CTSZ	TMEM176B	MLPH	RARA
ADAMDEC1	PDLIM4	USP2	SYT3	TFE3	MERTK	RARB
LGALS9B	GNLY	EFL1	FANCE	PKD2	RP11-757G1.6	RARG
TDRD10	AC011515.2	LILRB4	SKOR1	GPT2	C3AR1	RPS6KB1
CD14	PDGFA	AC016735.2	ADORA2BP1	STS	RP11-358L22.3	RXRA
ADAM28	LRRC25	MATN2	ZCCHC2	NMNAT2	SERPINB2	RXRB
CLMN	CXCL8	USP2-AS1	RAB37	ZNF44	CDKN1C	RXRG
RP1-229K20.5	PDZD7	EFCAB12	LBX2	C20orf197	CCR7	SREBF1
RP11-365P13.6	CTNNA2	CYP26B1	MLXIPL	PDZD2	F3	TGFB1
TREM1	MARCKS	RP11-356M20.1	TFEB	KIAA1683	TRPC5OS	THRA
CD7	MIR635	SMPDL3A	SPATA9	NCOR2	MORF4L2-AS1	THRB
GPER1	OSM	RP3-525N10.2	GAS7	RBM47	OLFML3	TNF
KRT35	CRISPLD2	SIGLEC17P	TMEM243	SH2D3C	DPYSL3	VDR
CDH15	ALOX5	ICAM1	CYP19A1	FGR	AGMO	
SLC12A1	RP11-321E2.3	CD300LB	INTS6L	HK1	NTNG1	
ABCB4	ANKRD22	DLGAP1-AS1	IRF5	WWC2	F13A1	
CD274	AC109826.1	MREG	SCPEP1	ANKRD10-IT1	IL1R2	
RN7SL834P	BCAS4	CTD-2521M24.8	IGFLR1	WIPI1	UNC79	
RP11-598F7.3	CDR2L	RP1-95L4.4	ITGB2	LRIG1	TCN1	
FBP1	ARHGEF16	SIRT4	NFE2	TRAF3IP3	TMEM52	
SLC37A2	KCTD12	PTGS1	TNRC6C-AS1	GADD45A	RP5-1028K7.2	
HRAT92	THEMIS2	RP11-432J24.5	CCDC170	SEPT3	TRNP1	
DENND6B	PNOC	DYSF	KCTD17	APBB2	COL2A1	
ABCD2	F12	FUCA1	C8orf31	ROS1	CH507-9B2.1	
NEGR1	RP11-23J18.1	LAMC3	MIR570	CCDC102A	AF064858.8	

CNR2	PEX5L	LILRA1	WLS	AC093642.3	CCL3L3
PDCD1LG2	CXCL1	CAV1	SAT1	RP11-960L18.1	RP11-121A14.2
FAM53B-AS1	SEMA6B	NCOA4	LRRC8A	ST3GAL4	LINC01202
TMEM151A	EPB41L1	IP6K3	P2RX7	TSPAN13	HES1
NTNG2	ANOS2P	ARSG	NR1I3	RTN4R	SMIM1
RP11-384O8.1	ORM1	AIG1	RP11-43F13.3	FBLN1	KIRREL3
BTNL9	TESPA1	SLC30A1	CHCHD10	FAM46C	GGT5
CXCL6	TFAP2C	SEL1L3	CTSD	RP11-1263C18.2	RP11-403A3.3
RHOH	PDGFRL	TRIO	BCO2	AC004987.9	SLC9C2
TRAF5	APCDD1	DNASE2	RP11-301G19.1	CTC-378H22.2	NAP1L4P1
SULF2	ITGAM	SYNJ2	FRMD3	PALD1	LINC01046
RP11-552D4.1	NRG2	MYO9B	ZFP92	TNFAIP3	RPSAP9
KCNF1	RP11-23F23.2	SIGLEC16	RNF165	FGFR3	CCND1
CD248	TLE6	SP100	CCRL2	CD6	TNFRSF19
ROPN1B	CTD-2342N23.3	LMF1-AS1	BCL6	EMID1	CCL3
SLC16A6	TRIM38	CDA	ACSL1	IL17RE	RP11-467J12.4
ARL5C	CD52	HIVEP2	CDK18	BEX1	SIM1
NBEAP1	NAPSB	IGFBP7	TBC1D2	SIX3	FAP
RP11-982M15.7	RP11-728F11.4	CHST2	C1orf101	MEGF6	BMP2
RP5-1116H23.3	SPOCK1	THBD	LGI2	LINC00884	BTBD11
GRPR	AC000403.4	CD79B	KCNK5	UGT3A2	DEPTOR
AC093609.1	GMPR	G0S2	MGAT4A	FOLR1	CXCR3
ORM2	RP11-309L24.10	MIR646HG	ADGRB3	NXT2	PLEKHA1
KCNK13	ITSN1	ZNF589	GM2A	RP11-96C21.2	RP11-55K13.1
TUBB4A	GABRG2	CECR1	TMEM169	PLCB4	RP11-445F6.2
RP11-429J17.7	CD38	KIAA1324L	OTOF	MTSS1L	LOH12CR2
AC021218.2	RP11-259K5.2	PTAFR	SLC24A4	GSN	MGAT4C
CPB2	RAVER2	PLXDC1	PPARGC1A	CHST4	RP11-572C15.5
NCF1C	NCF1B	TMEM156	FBXO41	PRR5L	RP5-1185I7.1
SPP1	CASC9	SFXN3	TMEM38A	NPTX1	CTD-3116E22.8
ACVRL1	FXYD6	TMEM176A	RP11-124O11.1	HES2	KANK4
HP	TRIM2	RASSF2	RP5-892K4.1	TRGC2	RNU1-22P
LINC00304	PLA2G16	NLRC4	CACNB1	SEMA6A	OCSTAMP
SLC17A7	ITGB5	PNPLA1	LPO	KHDRBS3	RP1-136B1.1
	SLC6A12	OXER1	SERPINB1	GPR176	CRYM
	RP11-321E2.4			WI2-87327B8.2	RP11-651L5.3
