# Supplementary material for: Activation of Vitamin D Receptor Pathway Enhances Differentiating Capacity in Acute Myeloid Leukemia with Isocitrate Dehydrogenase Mutations
Source: Cancers (Basel). 2021 Oct 19;13(20):5243. doi: 10.3390/cancers13205243 (PMC8533831; doi:10.3390/cancers13205243)
Supplement: Supplementary file 1 [file cancers-13-05243-s001.zip › Table S5.pdf]

Table S5. Clinical characteristics of AML patients (related to Figure 4).

Ps	1	2	3	4	5	6	7	8	9	10	11	12
ELN 2017	Adverse	Intermediate	Intermediate	Adverse	Intermediate	Favorable	Favorable	Intermediate	Adverse	Intermediate	Favorable	Favorable
F/M	F	M	M	M	M	F	M	M	M	M	M	F
Bone marrow	yes	yes	yes	yes	yes		yes		yes	yes		yes
Blood	no	no	no	no	no	yes	yes		no	yes	yes	
FAB	1	4	1	2	2	4	4	2	2	1	2	2
Caryotype	46,XX<20>	46,XY<22>	46,XY [20]	46,XY<20>	46,XY [20]	46,XY<20>	46,XY<20>	47,XY,+1,der(1;7)(q10;p10),+21<11>/46,XY<1>	46,XY [20]	46,XY<20>	46,XY,inv(16)(p13q22)<4>/46,sl,del(3)(p23p13),t(12;22)(p13;q12)<8>/45,sdl,-7<4>	
IDH1	R132H	R132H	no	no	no	no	R132H	R132G	no	no	no	no
IDH2	no	no	R140Q	R140Q	R140Q	R140Q	R140Q	no	R140Q	R140Q	yes	R140Q
NPM1	no	no	yes	no	no	yes	yes	no	no	no	no	yes
FLT3	yes ITD	yes ITD	yes (ITD)	no	no	TKD D835Y	es (TKD D835H	no	no	ITD	no	no (TKD nd)
CEBPA	no	no	no	no	no	no	no	no	no	nd	no	nd
DNMT3A	no	no	no	no	no	yes	nd	no	no	nd	no	nd
NRAS/KRAS	nd	nd	no	nd	no	nd	Nras	nd	no	nd	no	nd
Diag/relapse/refrac	diag	diag	diag	relapse	diag	relapse	diag	relapse	diag	diag	rechute	diag

Ps	13	14	15	16	17	18	19	20	21	22	23	24
ELN 2017	Favorable	Favorable	Adverse	Intermediate	Favorable	Adverse	Favorable	Adverse	Adverse	Favorable	Intermediate	Favorable
F/M	M	F	M	M	F	M	M		M	M	M	F
Bone marrow	yes	yes	yes	yes	no	no	no	no	no	no	no	no
Blood	no	no	yes	no	yes	yes	yes	yes	yes	yes	yes	yes
FAB	5	1	2	2	2	?	1	4	2	4	2	2
Caryotype	46,XY<20>	46,XX<20>	Complex	47,XY,+11<20>	46,XX,der(8)t(8;21)(q21;q22),der(14;21)(q10;q10)ct(8;21),+21c<7>/46,sl,der(16)t(13;16)(q14;q24)<12>/46,XX,rob(14;21)(q10;q10)c,+21c<2>	46,XY<21>	46,XY,t(8;21)(q22;q22)<20>	47,XX,+8 [20]	46,XY<20>	46,XY,del(7)(q32q36),inv(16)(p13q22)<18>/46,XY<3>	46,XY<20>	46,XX<20>
IDH1	R132H	no	no	no	no	no	no	no	no	no	no	no
IDH2	no	R140L	R140Q	R140Q	R140Q	no	no	no	no	no	no	no
NPM1	yes	yes	nd	no	no	no	no	no	no	no	no	yes
FLT3	no	TKD (D835Y)	nd	no	no	ITD	ITD	no	no	no	yes ITD	no
CEBPA	no	nd	nd	nd	no	no	no	no	no	no	no	yes
DNMT3A	no	no	nd	nd	no	no	no	no	no	no	no	yes
NRAS/KRAS	nd	no	nd	nd	nd	ND	ND	no	no	yes	ND	ND
Diag/relapse/refrac	diag	diag	Relapse	diag	relapse	relapse	diag	diag	diag	diag	diag	diag

Ps	25	26	27
ELN 2017	Intermediate	Itermediate	Intermediate
F/M	26/04/1946	M	M
Bone marrow	no	no	no
Blood	yes	yes	yes
FAB	2	5	2
Caryotype	46,XY<20>	46,XY<20>	46,XY<20>
IDH1	no	no	no
IDH2	no	no	no
NPM1	yes	yes	no
FLT3	yes ITD	ITD	no
CEBPA	no	no	no
DNMT3A	no	yes	no
NRAS/KRAS	nd	nd	yes
Diag/relapse/refrac	diag	diag	diag
